# Whole-Transcriptome Profiling of Canine and Human in Vitro Models Exposed to a G-Quadruplex Binding Small Molecule

**DOI:** 10.1038/s41598-018-35516-y

**Published:** 2018-11-20

**Authors:** Eleonora Zorzan, Ramy Elgendy, Mery Giantin, Mauro Dacasto, Claudia Sissi

**Affiliations:** 1 Department of Comparative Biomedicine and Food Science, University of Padua, Legnaro, Padua, Italy; 2 Department of Immunology, Genetics and Pathology, Uppsala University, Uppsala, Sweden; 3 Department of Pharmaceutical and Pharmacological Sciences, University of Padua, Padua, Italy

## Abstract

G-quadruplexes (G4) are secondary nucleic acid structures that have been associated with genomic instability and cancer progression. When present in the promoter of some oncogenes, G4 structures can affect gene regulation and, hence, represent a possible therapeutic target. In this study, RNA-Seq was used to explore the effect of a G4-binding anthraquinone derivative, named AQ1, on the whole-transcriptome profiles of two common cell models for the study of *KIT* pathways; the human mast cell leukemia (HMC1.2) and the canine mast cell tumor (C2). The highest non-cytotoxic dose of AQ1 (2 µM) resulted in 5441 and 1201 differentially expressed genes in the HMC1.2 and C2 cells, respectively. In both cell lines, major pathways such as cell cycle progression, KIT- and MYC-related pathways were negatively enriched in the AQ1-treated group, while other pathways such as p53, apoptosis and hypoxia-related were positively enriched. These findings suggest that AQ1 treatment induces a similar functional response in the human and canine cell models, and provide news insights into using dogs as a reliable translational model for studying G4-binding compounds.

## Introduction

Targeted therapy is a novel strategy for cancer treatment that aims to destroy cancer cells with more precision and, potentially, fewer side effects^1^. For the discovery of clinical agents targeting oncogenic signaling pathways, it is important to define the compound’s specificity towards the target molecular pathway. In this respect, the field of cancer genetics and genomics is rapidly evolving, particularly focusing on targeted pathways that are critically linked to a malignant behavior^2^. Currently, multiple compounds are being used to treat cancer and many more are being tested in clinical trials^3–5^.

Canine/human comparative oncology is recently playing an increasingly important role in advancing cancer research from basic science, preclinical and clinical perspectives. The acknowledgment of spontaneous cancer development in companion animals and the potential of using these animals in drug development studies and preclinical trials is harnessed by the morphologic, histologic and biological similarities between canine and human spontaneously-occurring malignancies. Dogs’ physical size, accessibility to serial biological sample collections, comparable tumor biology, intact immunity and short survival compared to humans led to their inclusion as a complementary animal model^2^. Inter-species cancer studies have contributed to gaining new insights into the biology of breast cancer, osteosarcoma, lung and bladder cancer^6–10^. Further, recent studies on canine B-cell lymphoma demonstrated that recurrent aberrations were correlated with cancer subtype, and the corresponding cytogenetic lesions were also noticed in human patients^11,12^. Meantime, another study showed that canine melanoma possesses rare mutations in the B-Raf (*BRAF)* and N-ras (*NRAS)* proto-oncogenes; however, they present similar gene expression changes to human melanoma within the same downstream activation pathways^13^. Thus, although the oncogenic driving somatic mutations between the two species may differ, similar upregulation and signaling pathways underscore the translational value of studying tumor biology from a comparative point of view. This concept might support the beginning of the so-called ‘oncological basket’ trials, wherein the response of tumors with shared credentialed biology to a specific targeted therapy are assessed beyond histologic diagnosis^2^.

In the past decade, several studies have been focusing on guanine-rich stretches found in important genomic regions such as telomeres, centromeres, immunoglobulin switch regions, mutational hot spots, and promoter elements^14^. These guanine-rich sequences may have the potential to form four-stranded secondary structures called G-quadruplexes (G4) that are stabilized by non-Watson-Crick interactions (Hoogsteen bond^15^). Several studies clarified that G4 structures are often linked to important DNA regions involved in the regulation of gene transcription and translation^16,17^. Moreover, studies have shown that several proto-oncogenes like v-myc avian myelocytomatosis viral oncogene homolog (*MYC*), v-kit Hardy-Zuckerman 4 feline sarcoma viral oncogene homolog (*KIT*), B-cell lymphoma 2 (*BCL2*), vascular endothelial growth factor (*VEGF*), platelet-derived growth factor (*PDGF*) and hypoxia-inducible factor (*HIF*) bear G4 motifs in their promoters^16,18–24^. Specific proteins, which selectively bind G4 structures, have been discovered impinging on the biological roles of G4^25^. These motifs could be efficiently targeted by G4-selective ligands, thus altering their molecular recognition and, consequently, affect their downstream expression^25^.

More than 55% of the genes in the human genome bear at least one potential G4 motif 1 kb up- or downstream of the transcription starting site (TSS); hence, it is important to carefully assess whether the treatment with G4 binding compounds could affect the transcriptional activity of these genes^26^. Using the G4-stabilizing molecule TMPyP4, previous studies showed genome-wide effects on transcription in human cells; moreover, relatively high doses of TMPyP4 promoted cell migration and apoptosis in human cell lines^27–29^. Likewise, Halder and colleagues demonstrated that bisquilinium compounds were able to induce quadruplex-specific transcriptomic changes in HeLa S3 cells^26^. Marchetti *et al*., in 2018, used the RNA-Seq technology to study the effects of a new G4 binding compound in pancreatic ductal adenocarcinoma^30^.

Recently, we demonstrated that an anthraquinone derivative (named AQ1, Supplementary Fig. S1) significantly downregulated *KIT* mRNA and protein levels in several human cancer cell lines, thereby preventing their proliferation^31^. Meantime, two *KIT* G4-forming sequences (d_kit1 and d_kit2) were discovered in the canine genome (Supplementary Fig. S1)^32^. They showed a high degree of homology with the corresponding human sequences, and preliminary studies were conducted to assess their interaction with AQ1^33^. These studies on G4 structures were executed using a targeted-gene approach; nevertheless, it is of interest to extend the study of AQ1 transcriptional effects to a wider scenario, like the whole-transcriptome. Such an approach is expected to: (1) clarify the AQ1 influence on *KIT*-related pathways and (2) unveil specific off-targets of AQ1 in species-specific cells. Therefore, in the present study, we investigated - using RNA-Seq- the AQ1-induced effects on the global gene expression in two species-specific *KIT*-dependent cancer cell lines, in order to identify which cellular pathways are likely to be affected by treatment. For this purpose, we used the HMC1.2 human mast cell leukemia and the C2 canine mast cell tumor cell line, which are two common models for the study of tyrosine kinase inhibitors and, in particular, the proto-oncogene *KIT*.

## Results

### Effects of AQ1 on cell cytotoxicity and *KIT* and *BCL2* mRNA levels

We previously demonstrated that AQ1 significantly downregulated *KIT* and *BCL2* oncogenes in human mast cell leukemia HMC1.2, where the major effect of the treatment was observed 12 hours (T_12_) post-exposure^31^. Thus, we selected this incubation time to follow the activity of AQ1 on the two cell lines used in this study. Proliferation curves (Supplementary Fig. S2) demonstrated that, after 12 hours of incubation, AQ1 was moderately cytotoxic, with 70–90% cell survival in both the two cell lines at the tested concentration range (0–10 μM). Additionally, quantitative real-time PCR (qPCR) showed a significant dose-dependent reduction of *KIT* and *BCL2* mRNA levels in both HMC1.2 (*p* < 0.01) and C2 (*p* < 0.001) cells (Supplementary Fig. S3).

### Whole-transcriptome profiling

To account for any possible vehicle- (dimethyl sulfoxide, DMSO) related effects on cell gene expression, we also profiled the transcriptome of untreated cells (treatment- and vehicle-free medium). No differentially expressed genes (DEGs) were detected in the DMSO-treated cells compared with the untreated ones (results not shown); consequently, the DMSO-treated cells dataset was used as control.

The RNA-Seq analysis of the AQ1-treated HMC1.2 cells, compared with DMSO-treated cells, evidenced 3644 and 5441 DEGs (false discovery rate, FDR <0.05 and fold-change, FC >2) at 1 µM and 2 µM AQ1 concentrations, respectively. For both concentrations, more than 60% of the total DEGs were downregulated. In the AQ1-treated C2 cells, the whole-transcriptome analysis identified 397 and 1201 DEGs at 1 µM and 2 µM AQ1 concentration, respectively. Likewise to HMC1.2, more than 70% of these DEGs were downregulated. The whole list of the DEGs for both cell lines is reported in Supplementary Dataset S1 and a list of the common downregulated genes between HMC1.2 and C2 is reported in Supplementary Dataset S2.

**Figure 1:**
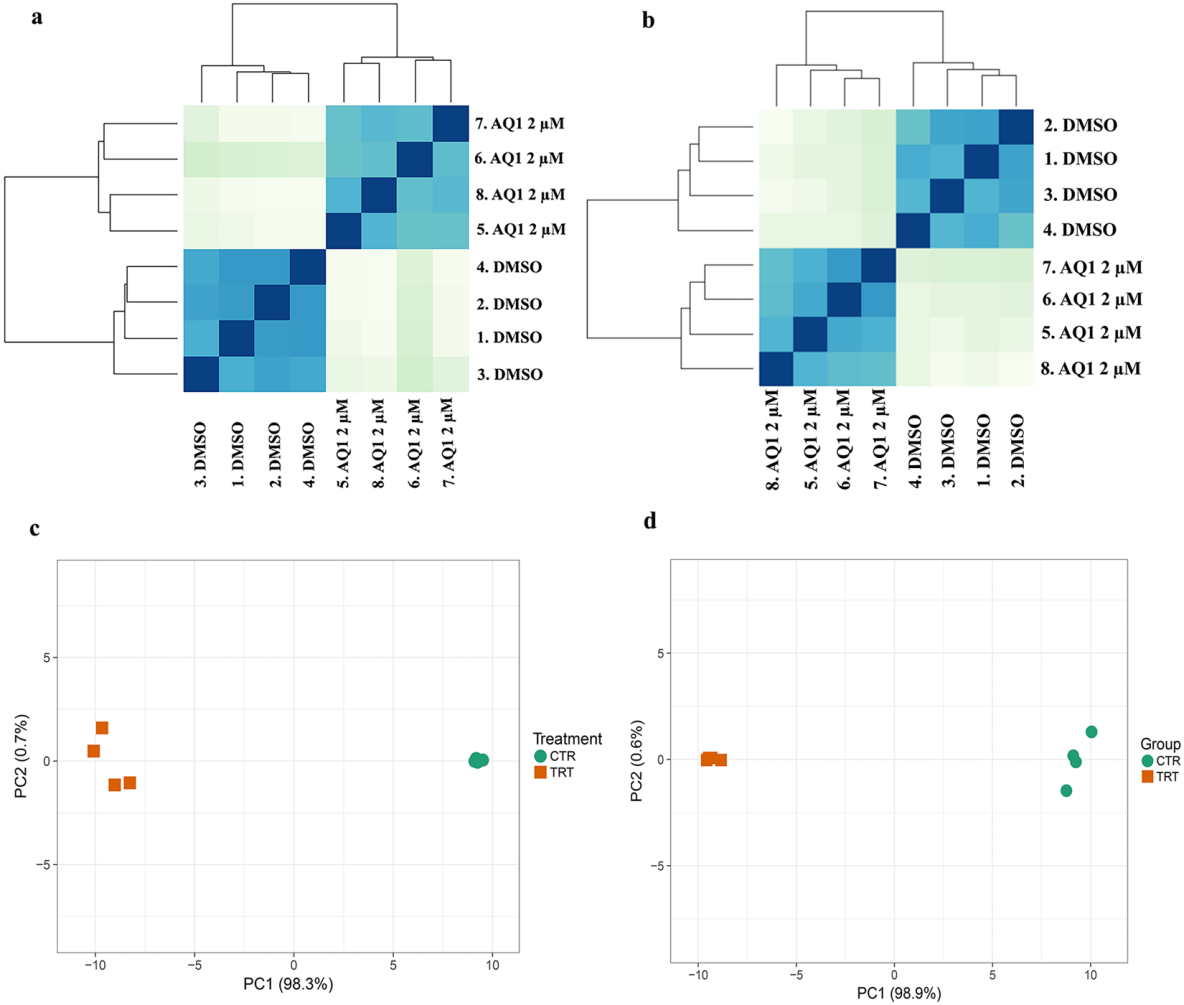
Analysis of intra-population consistency: hierarchical clustering and PCA. (**a**,**b**) Hierarchical clustering analyses performed using DESeq2 rlog-normalized RNA-Seq data. (**a**) HMC1.2 cells, AQ1 (2 µM) vs DMSO. (**b**) C2 cells, AQ1 (2 µM) vs DMSO. Color code (from white to dark blue) refers to the distance metric used for clustering (dark blue corresponds to the maximum of correlation values). (**c**,**d**) Principal Component Analysis (PCA) performed on top 100 differentially expressed genes using ClustVis (Metsalu & Vilo, 2015) tool. Loadings PC1 and PC2 are reported in graph (on x and y-axes). (**c**) HMC1.2 cells, AQ1 (2 µM) vs DMSO. (**d**) C2 cells, AQ1 (2 µM) vs DMSO.

Both experimental groups clustered uniquely on a hierarchical clustering plot (Fig. 1a,b), and a Principal Component Analysis (PCA) plot, using the top 100 DEGs, resulted into two distinct clusters of samples with the principal component 1 (PC1) accounting for the major variation between groups (PC1 >98%; Fig. 1c,d). This variation between AQ1-treated and control groups is further visualized using two heatmaps that are shown in Supplementary Fig. S4.

### Gene set enrichment analysis (GSEA) and protein-protein interactions

**Figure 2:**
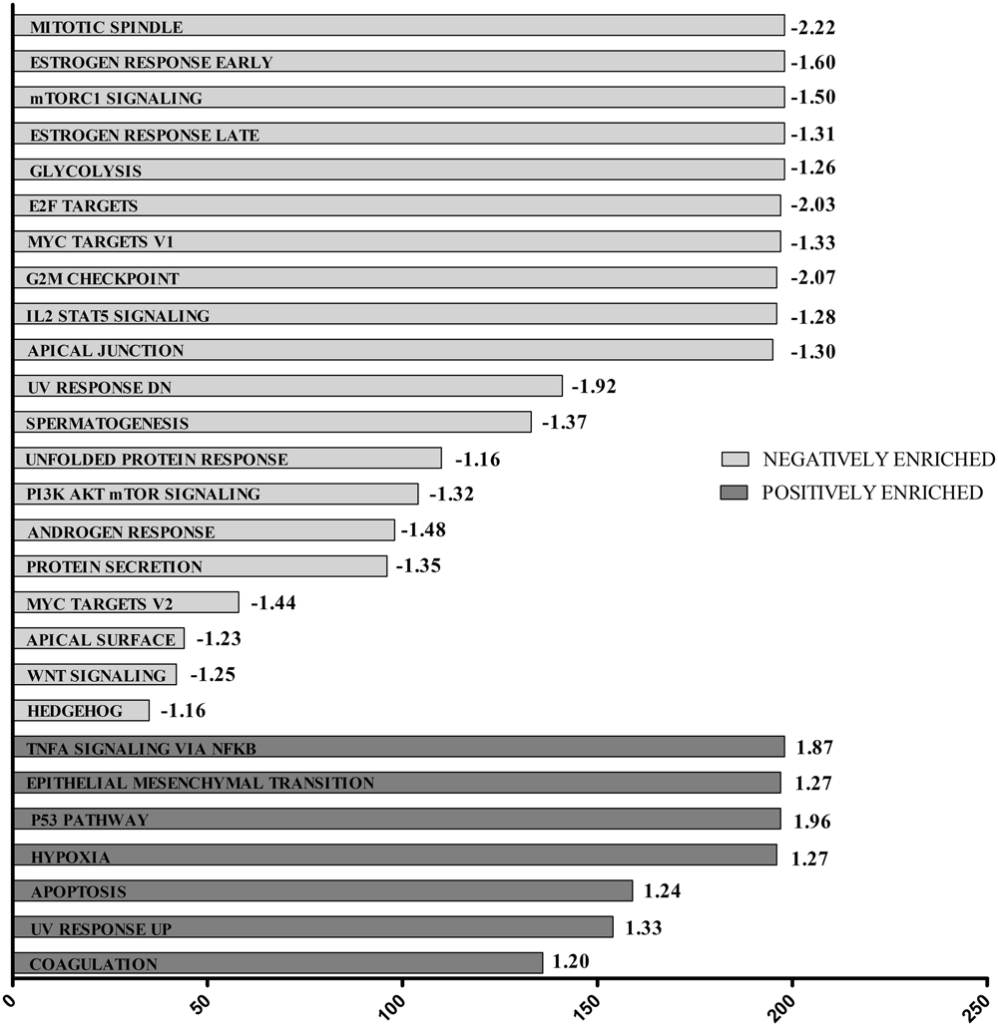
GSEA cancer hallmarks positively and negatively enriched after treatment with 2 μM AQ1 in HMC1.2 cells. The x-axis reported the gene group size and numbers at the end of the bars represented NES value.

**Figure 3:**
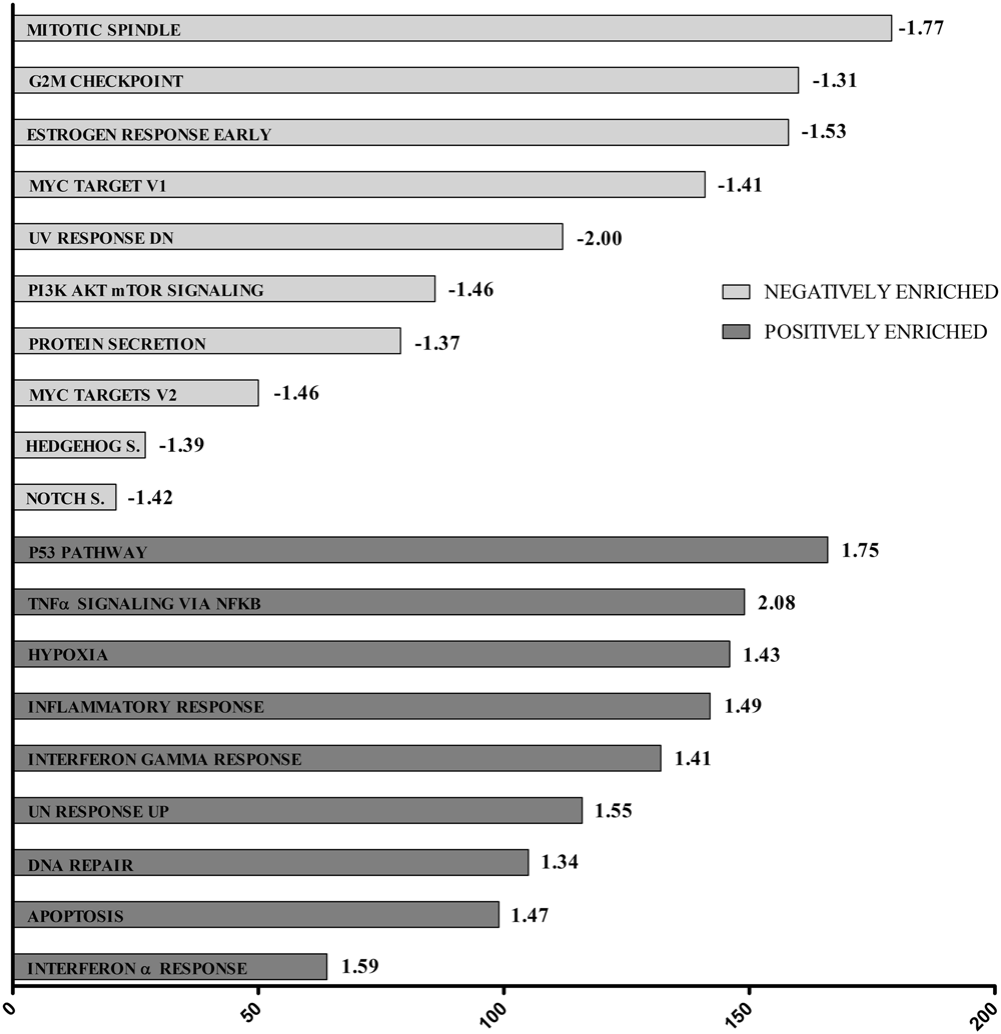
GSEA cancer hallmarks positively and negatively enriched after treatment with 2 μM AQ1 in C2 cell line. The x-axis reported the gene group size and numbers at the end of the bars represented NES value.

**Figure 4:**
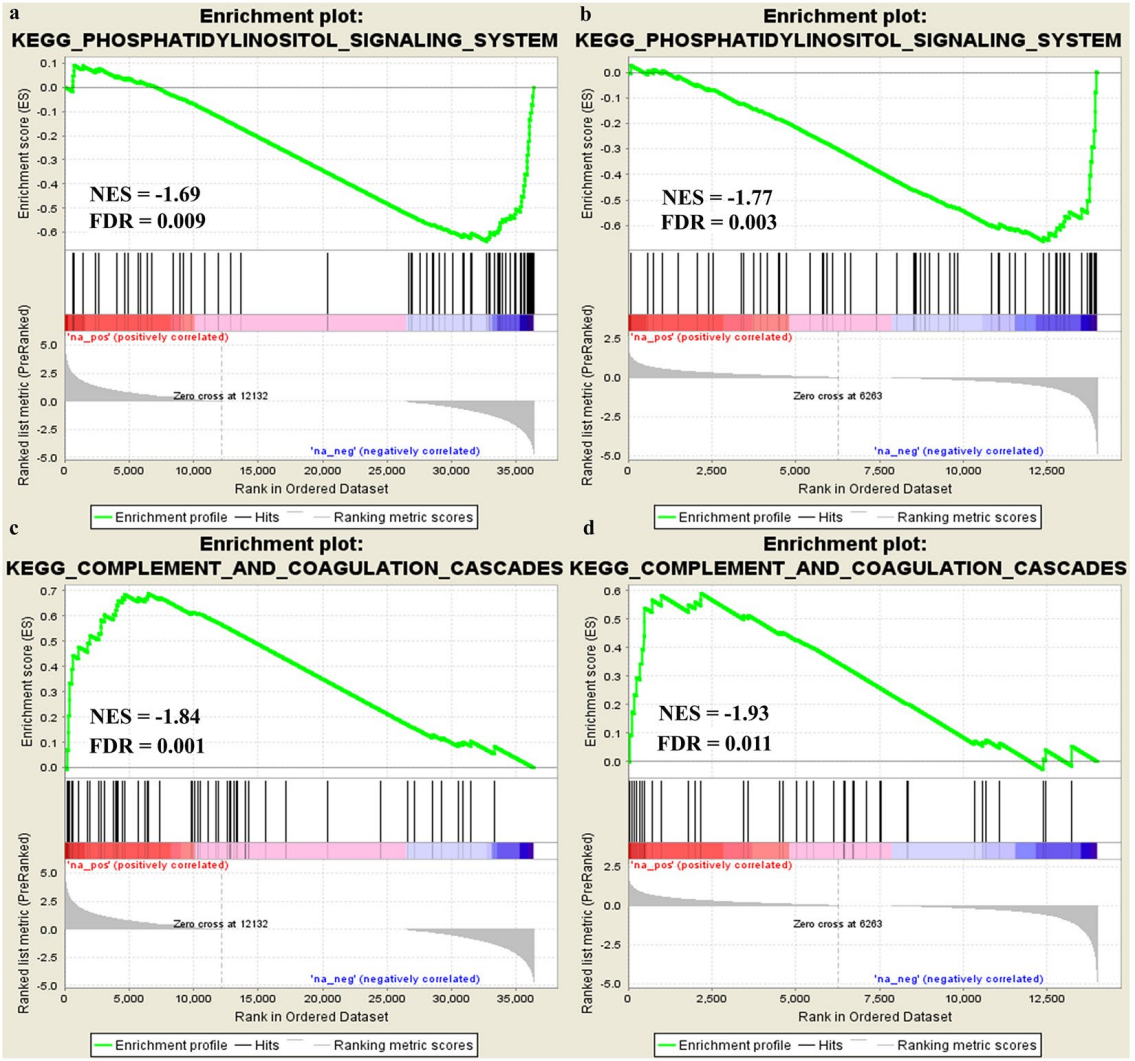
GSEA Enrichment plot (KEGG pathways) negatively and positively enriched in HMC1.2 and C2 cell lines exposed to AQ1 2 μM. The phosphatidylinositol signaling system was negatively enriched by AQ1 in HMC1.2 (panel a) and C2 (panel b). The complement and coagulation cascades pathways was positively enriched by AQ1 in HMC1.2 (panel c) and C2 (panel d). The green curve corresponds to the ES (enrichment score) curve, which is the running sum of the weighted enrichment score obtained from GSEA software, while the normalized enrichment score (NES) and the corresponding FDR are reported within each graph. A permission for the use of the images was obtained by KEGG (Kanehisa Laboratories rif. 180271)^34–36^.

Canine C2 and human HMC1.2 cell lines showed similar functional response to AQ1 treatment. Figures 2 and 3 show the most positively- or negatively-enriched cancer hallmarks in the AQ1-treated (2 µM) HMC1.2 (Fig. 2) and C2 cells (Fig. 3). In HMC1.2 cells (Fig. 2), the most negatively-enriched hallmarks were the mTORC1 signaling pathway (FDR = 0.017), the targets of *MYC* (FDR = 0.026), the G2M cell cycle checkpoint (FDR = 0), and the mitotic spindle (FDR = 0). As shown in Fig. 3, AQ1-treated canine C2 cells show a similar functional response to the human HMC1.2 cells. Further, KEGG pathways analysis (Supplementary Tables S1–S4) identified many pathways that were negatively-enriched in the AQ1-treated samples, in both cell lines^34–36^. For instance, cell-cell adherent junctions (HMC1.2, FDR = 0.009; C2, FDR = 0.043), mismatch repair (HMC1.2, FDR = 0.015; C2, FDR = 0.041), the regulation of actin cytoskeleton (HMC1.2, FDR = 0.012; C2, FDR = 0.036), the DNA replication machinery (FDR = 0.008), the tRNA biosynthesis (FDR = 0.012) and the cell cycle pathway (FDR = 0.014). Figure 4 (panels a,b) shows the downregulation of phosphatidylinositol signaling system (*p* ≤ 0.001) KEGG pathway in HMC1.2 and C2, respectively. Moreover, the complement and coagulation cascade were the most important KEGG pathway positively enriched in both cells (Fig. 4c,d). Supplementary File 1 report, more in details, the most important pathways identified by KEGG GSEA analysis in HMC1.2 and C2, respectively. This functional similarity was also confirmed by the The Database for Annotation, Visualization and Integrated Discovery (DAVID) tool (data not shown). Also, several important cancer hallmarks, such as P53- and apoptosis-related pathways as well as hypoxia and tumor necrosis factor α (TNFα) pathways related to NF-κβ, were positively enriched (FDR < 0.05) in both cell lines (Figs 2 and 3).

**Figure 5:**
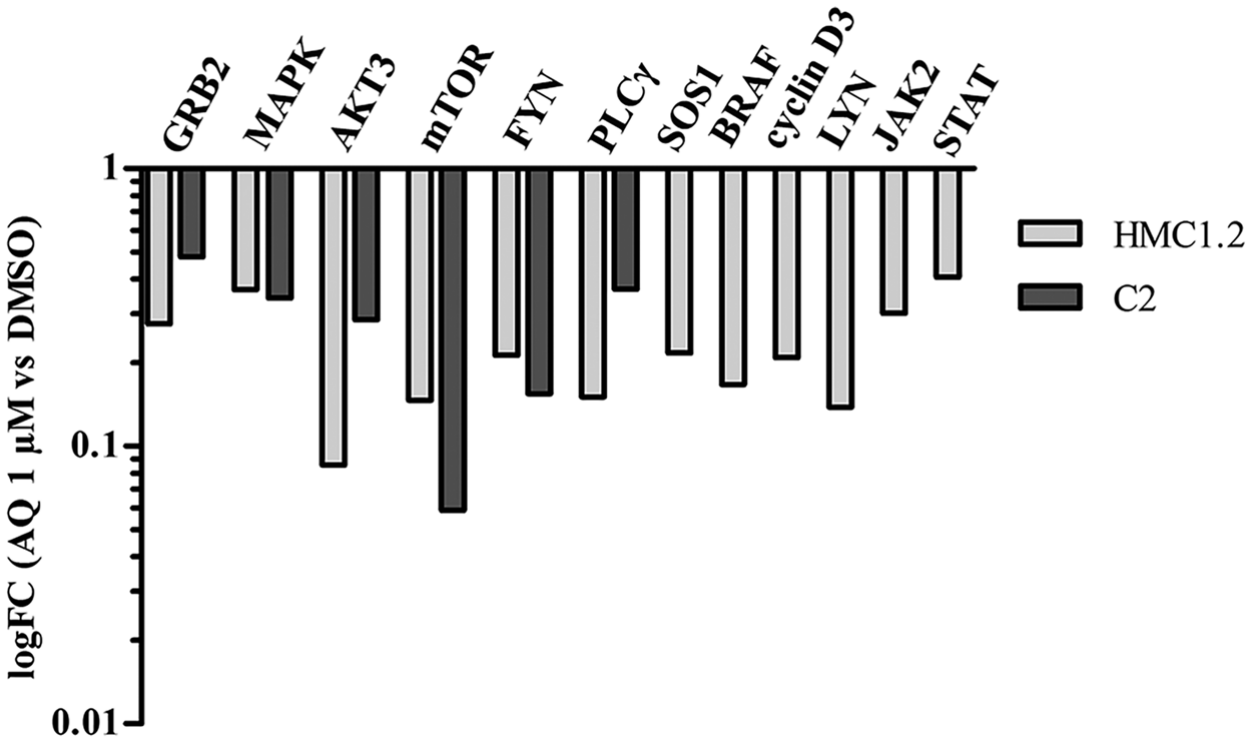
RNA-Seq fold change (FC) of genes involved in *KIT* downstream pathways after treatment with AQ1 2 µM. *GRB2*: Growth Factor Receptor Bound Protein 2. *MAPK*: Mitogen-Activated Protein Kinase 1. *AKT3*: AKT Serine/Threonine Kinase 3. *mTOR:* Mechanistic Target Of Rapamycin Kinase. *FYN*: FYN Proto-Oncogene, Src Family Tyrosine Kinase. *PLCγ2*: Phospholipase C Gamma 2. *SOS1*: SOS Ras/Rac Guanine Nucleotide Exchange Factor 1. *BRAF*: B-Raf Proto-Oncogene, Serine/Threonine Kinase. *LYN*: LYN Proto-Oncogene, Src Family Tyrosine Kinase. *JAK2*: Janus Kinase 2. *STAT*: Signal Transducer And Activator Of Transcription 1.

With regard to *KIT* regulation, the RNA-Seq data evidenced a significant inhibition of multiple downstream processes activated by this proto-oncogene. Figure 5 shows the expression profile (mRNA) of some genes that are part of several *KIT* downstream pathways such as the Ras/Erk signal transduction, the PI3K signal transduction, the PLC-γ signaling transduction, the Src kinase signal transduction and the JAK/STAT signaling pathways^37^. In both cell lines, the whole set of target genes were negatively enriched following the AQ1 treatment, except for the canine JAK-STAT pathway.

**Figure 6:**
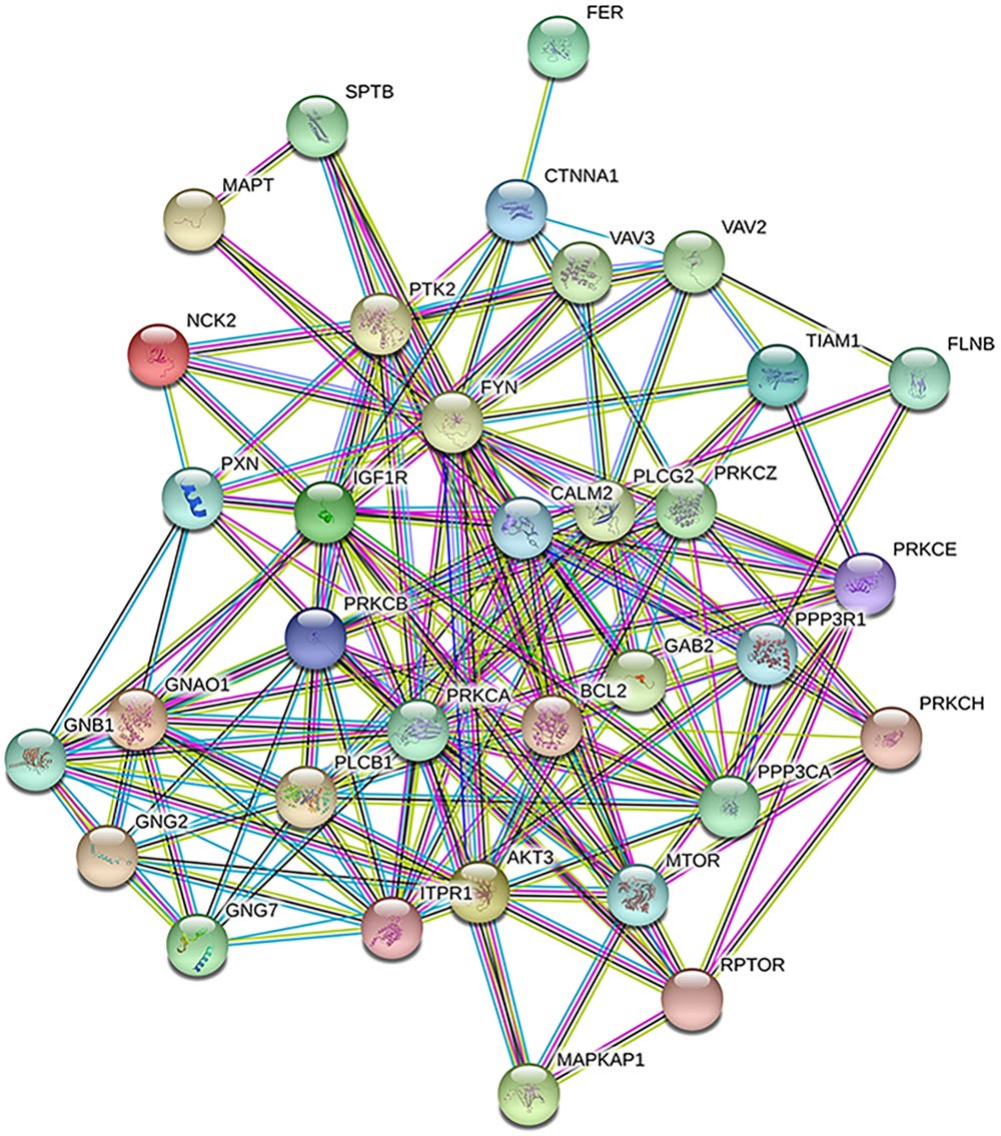
STRING protein network map of thirty proteins commonly downregulated by AQ1 2 µM in HMC1.2. Colored nodes indicate the individual protein identified. Lines between nodes represent direct and indirect association of proteins. Line colors indicate the type of interaction evidence (light blue: known interaction from curated database; purple: known interaction experimentally determined; green: predicted interaction gene neighborhood; red: predicted interaction gene fusion; blue: predicted interaction gene co-occurrence; yellow: text mining; black: co-expression; lilac: protein homology.

To uncover some of the biological consequences the DEGs might have, the top downregulated protein-coding genes were collapsed to their corresponding protein symbols and their interactions were visualized by a protein-protein interaction (PPI) network, using the Search Tool for the Retrieval of Interacting Genes/Proteins (STRING, https://string-db.org/) database. Figure 6 shows the PPI network (*p* < 0.001) observed in HMC 1.2 cells exposed to 2 µM AQ1. The most negatively-enriched pathways were the transmembrane receptor protein tyrosine kinase signaling pathway (GO.0007169; FDR <0.001), the vascular endothelial growth factor receptor signaling pathway (GO.0048010; FDR <0.001), and the cell surface receptor signaling pathway (GO.0007166; FDR <0.001). Because the STRING database relies largely on curated experimental data, a meaningful PPI for the DEGs of C2 cells was not possibly – likely because little information is available on the function of proteins in dogs.

### G4 prediction

**Figure 7:**
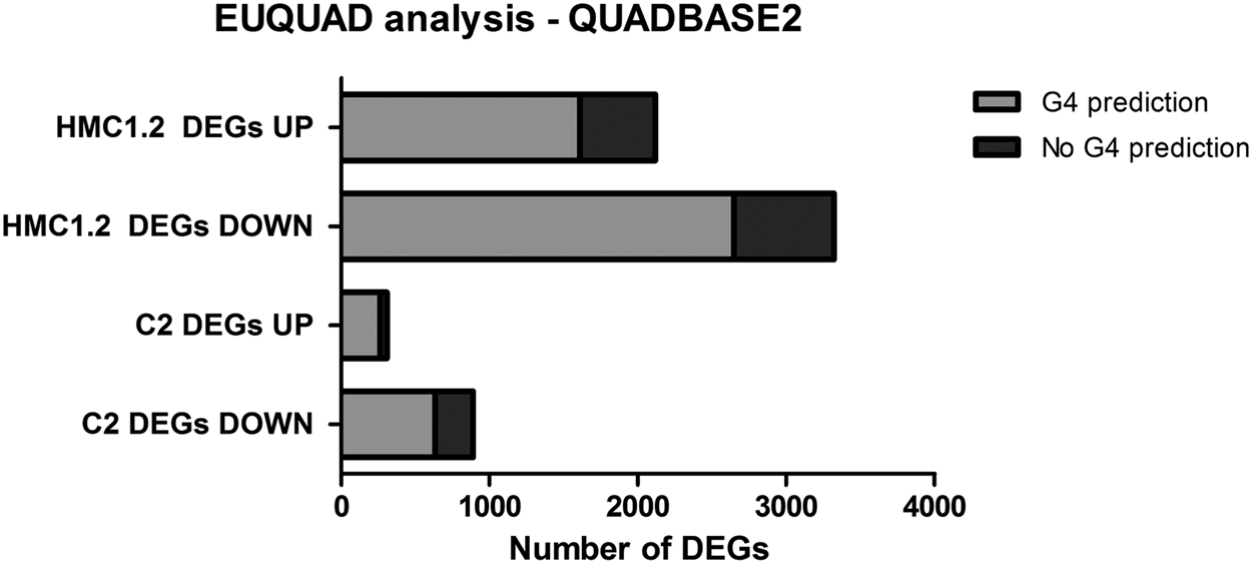
Number of DEGs that are predicted by EuQuad tool to have at least one putative G-quad sequence in the region ±2 kb TSS (G3 L1-7).

The Quadbase2/EuQuad tool^38,39^ was used to mine G4 from RNA-Seq results using the corresponding gene’s Ensembl ID. Figure 7 reports the results obtained searching for the G3 L1–7 motif in ±2 kb TSS of each record. In both species, EuQuad predicted the presence of at least one G4 domain near the TSS for the majority of DEGs (overall >75%). The percentage of DEGs with a predicted G4 domain did not differ between the down- and up-regulated genes (71% vs 85.8% in canine DEGs and 80% vs 76% in human DEGs, respectively).

### qPCR confirmation

To cross-validate the RNA-Seq data, twelve genes were tested for their relative gene expression by qPCR. The selected genes belonged to different pathways: *KIT* activation pathways (*RPTOR)*, epigenetic regulation (*HDAC4)*, apoptosis (*BMF*), cell cycle (*CDKN1A*, *CDK6*), metabolism (*CYP1A1*, *FAXDC2*), cell signaling (*KRS2*, *TNFRSF11β*), transcription regulation (*EGR2*), cell morphology (*MID1*), and tumor oncogenes (*EGFR)*. The qPCR results (Supplementary Figures S5–S7) corroborated the RNA-Seq data analysis. In specific, the downregulation of *RPTOR* and *HDAC4* by AQ1, as well as the upregulation of the pro-apoptotic factor *BMF* and cell cycle controller *CDKN1A* were significantly correlated between the qPCR and RNA-Seq data, in both cell lines.

The EuQuad output for each gene selected for qPCR confirmation is shown in Supplementary Table S5. For every gene, the software analyzed around 2 kb before and after the TSS to search for G3 L1-7 motif. *HDAC4* was the gene with the highest number of putative G4-forming sequences near the TSS in both species, followed by *CDK6* in human and *RPTOR* in dog. On the contrary, for other genes like canine *EGFR*, human *CDKN1a* and human *EGR2* the tool did not find G3 L1-7 motifs.

## Discussion

DNA is one of the main targets of most anticancer drugs. However, it is rather difficult to achieve a selective effect on particular DNA regions because of the high homogeneity of cellular DNA, mostly occurring in the B form. Interestingly, the possible interactions with specific non-canonical DNA structures may improve the selectivity of low molecular weight compounds towards certain cell processes or signaling pathways. In this scenario, an increasing interest has recently gained targeting the G4 secondary structure of nucleic acids that might affect pathways that are critical for tumor cells growth and proliferation^40^.

We previously used a targeted approach to assess the effect of AQ1 both in human and canine *KIT*-dependent cell lines (from two to almost forty-fold inhibition)^31–33^. Results demonstrated a significant inhibition of cell proliferation and downregulation of *KIT* and *BCL2* induced by AQ1 whereas other prototypical oncogenes as *hTERT*, *MYC*, and *PDGF* were not highly modulated by this compound. Moreover, we confirmed the presence of two conserved G4 forming domain in canine *cKIT* promoter^32^.

Taking into consideration these evidence, in the present study, we profiled, using RNA-Seq, the effects of AQ1 treatment on the transcriptome of two *KIT*-dependent cell lines of human and canine origin (HMC1.2 and C2, respectively). The choice of time and doses derived from a preliminary validation step; in particular, the two doses we used were poorly cytotoxic for both cell lines, while the time of exposure (T_12_) was sufficiently short to attribute the major transcriptional modulation to DNA binding by AQ1 and not to secondary effects that could occur over a longer treatment time. Indeed, the occurrence of such a drawback emerged in previous transcriptional works. For instance, Halder and colleagues (2012) performed a transcriptional profiling with microarray after 48 h of treatment; because of the long-time of treatment, it was not possible to rule out whether or not some or many of the observed effects were attributed to a direct G4 formation or to secondary effects^26^. Furthermore, HeLa S3 and K562 cells exposed to the G4-stabilizing molecule TMPyP4, showed genome-wide transcriptional effects. However, a smaller set of affected genes (69 and 87 genes for HeLa S3 and K562 cells, respectively) were identified at prolonged (48 h) time of exposure to relatively high TMPyP4 concentration^27,28^ (100 μM).

In this study, upon cell treatment with AQ1, we noticed a dose-dependent increasing number of DEGs; moreover, an overall major inhibitory transcriptional effect was observed in both cell lines. This is in line with several studies indicating G4 as a transcription suppressor element^41–43^. The ligand-induced stabilization of G4 motifs could lead to “jamming” of the transcription bubble, thus resulting in stalling of RNA polymerase and in an aborted transcription^27,44^. This is also in agreement with the suppression of gene expression that we observed in our experimental conditions. The use of EuQuad clarified that most of up- or downregulated DEGs possessed at least one G3 L1-7 motif near the TSS. Nevertheless, this is just an *in-silico* prediction and other approaches, e.g. the Chem-seq technique and G4-Seq or G4 ChIP-seq methods, could give more precise information about AQ1 and G4 interactions on the whole genome level^45–47^.

On a comparative basis, we observed a higher number of DEGs in the human *versus* the dog cell line. This result supports preliminary analysis on C2 cells where a slight reduction of *KIT* mRNA and protein levels was observed upon treatment with AQ1^33^. This can be reasonably connected to the different sequence and distribution of the G4 forming domains in the human *versus* canine promoters although the modulation of distinct cellular pathways cannot be ruled out. Indeed, a critical issue might be represented by the fact that the two cell lines we used in this study represent, in both species, suitable *in vitro* models for mast cell diseases. However, they derive from different neoplasms, i.e. human mastocytosis (HMC 1.2) and cutaneous mast cell tumor (C2). Hence, this study does not represent a mere transcriptome comparison between the two species since apparent contradictions might result from gene and/or pathways differential expression in the two cell lines. However, despite the possible presence of substantial transcriptional differences between the two cell lines, several common cancer hallmarks and oncogene-related pathways were identified. In particular, the mTOR- and MYC-dependent pathways were downregulated in both species, whereas the apoptosis and p53-mediated programmed cell death were positively enriched. Other ligands have already been investigated for their ability to stabilize the G4 structure of *MYC* and *KIT* promoter, thereby affecting their downstream pathways. In Goh *et al*. (2016), a salicylaldehyde semicarbazone copper (II) complex induced apoptosis through the caspase-dependent pathway by reducing the expression of *RAS* and *MYC*, and by inhibiting Ras-ERK and PI3K-Akt pathways in MOLT-4 leukemia cells^48^. In 2013, Shen and colleagues proved that a G4 ligand named SYUIQ-FM05 suppressed *KIT* mRNA transcription and total kit protein amount in K562 cells, and this inhibition led to a downregulation of MEK activity, ERK phosphorylation, and the RAS/MEK/ERK signaling pathway^49^.

If we consider as a whole the GSEA hallmarks and KEGG pathways upregulated by AQ1, there is a predominance of immune system-related pathways. In this respect, no information is available on the stimulation of immune system by other G4 ligands; hence, this could represent an interesting and positive aspect to be considered in further studies.

Assuming a major effect of AQ1 at oncogenes promoters possessing G4 structures, we analyzed the most important pathways regulated by some of the most common oncogenes, such as *MYC*, *BCL2*, *PDGF*, *hTERT* and *VEGF*. Considering *MYC*-related pathway, AQ1 caused the downregulation of *E2F*, *MDM2*, *p53*, *p73* and *BCL6* in HMC1.2 cells. Interestingly, the latter two genes showed a similar behavior also in canine cells. *BCL2* was modulated by the AQ1 treatment in both cell lines with the consequent increase in apoptosis-related pathways confirmed by the increasing expression of pro-apoptotic factors as BIK, BMF and NOXA (the last one only in HMC1.2 cells). In general, it is well demonstrated as anthraquinone derivatives have been shown to induce apoptosis *in vitro*, and such a phenomenon usually implies a decrease of *BCL2* mRNA/protein^50–53^. The *PDGF* signaling pathway was affected only in HMC1.2 cells, with the downregulation of *TIAM2*, *VAV1*, *VAV2* and *NF-kβ*. No variation in mRNA levels of *TERT* gene were noticed in both cell lines, despite the important role of G4 sequences in its regulation and transcription^54^. Finally, VEGF pathway was only partially influenced by AQ1 in human cells, as shown by caspase 9, *GRB2* and *SOS* downregulation. In dog cell line, VEGF pathway was negatively enriched by GSEA, likewise to human.

Interestingly, in this study we identified new oncogenes affected by AQ1; for example, the gene *FYN* was downregulated in both cell lines. *FYN* is a tyrosine kinase proto-oncogene with a role in proliferation, invasion and migration in human thyroid carcinoma^55,56^. It also plays a critical role in the development, progression and resistance to anti-cancer drugs in breast cancer, prostate cancer and leukemia^56^. No information is available about the presence of G4 in its promoter sequence. On the other hand, two G4 forming sites are present in the human *EGFR* promoter^57^, a transmembrane protein activated by the epidermal growth factor and the transforming growth factor alpha (TGFα). This oncogene was downregulated by AQ1 only in dog, but other G4 ligands have been shown to affect its mRNA levels in a previous human transcriptomic study^26^.

The proto-oncogene *JUN* was upregulated in HMC1.2 (FC = 18) but not in canine cells. Consistent knowledge shows that c-jun contributes to tumor initiation and increased invasiveness^58^. However, few studies discovered some alternative activities of c-jun, suggesting that it may act as a double-edge sword in cancer^58^. As an example, c-jun seems to prevent methylation silencing of p16INK4a, a tumor suppressor and a cell cycle inhibitor gene^59^. Moreover, the anticancer mechanism of action of tylophorine, a plant-derived alkaloid, is mediated by c-jun, resulting in G1 cell cycle arrest in carcinoma cells, occurring through the downregulation of cyclin A2^60^.

Despite the innovative results obtained in this study, several limitations should be considered. Probably, the most important one is the absence of a validation step for the RNA-Seq platform at the protein level. The post-transcriptional mechanisms of regulation and the emerging evidence that 5′- and 3′-untranslated regions (5′- and 3′-UTRs) as well as open reading frames (ORFs) contain putative RNA G4, might play an important role in tumor biology, e.g., switching on/off some tumor-related pathways^61^. From previous data, the AQ1-mediated downregulation of *KIT* was confirmed also at the protein level in human and canine *in vitro* models^31–33^. Hence, the protein evaluation of the kit-downstream pathways could substantiate current transcriptional results. Secondly, in the present study we considered one G4 ligand compound and only tumor cell lines. In perspective, it could be of interest to extend the RNA-Seq analysis also to a non-tumor cell line, to distinguish any non-specific effects. From previous information obtained in MDCK cells, these are much more resistant to AQ1 in term of cytotoxicity and survival^33^, but a more in-depth study investigating also non tumor-specific pathways is still missing. Finally, in terms of pathways analysis (DAVID), a limit derived by the actual scarce annotation of dog genome in comparison with the human one, and this should be consistently taken into consideration in the approach to future studies. Nevertheless, the present work represents the first step to validate dog as suitable translational model with reference to the use of G4-binding compounds.

In conclusion, the present study showed that AQ1 G4-binding compound inhibited *KIT* expression and its downstream signaling molecules *GRB2*, *AKT* and *FYN* in two *in vitro* models of human and canine mast cell neoplasms (HMC1.2 and C2 cells). Interestingly, besides *KIT*, AQ1 negatively affected the *MYC*-related pathway whereas it induced both apoptosis and P53-related pathways. Overall, these results suggest a possible role of AQ1 in blocking mast cells proliferation via different pathways, thus representing a potential therapeutic target for comparative mast cell tumors.

## Methods

All methods were carried out in accordance with relevant guidelines and regulations generally used for handling human and animal cell lines in the Department of Comparative Biomedicine and Food Science and at the University of Padua.

### Ligand

AQ1 was synthesized by Prof. G. Zagotto (University of Padua, Italy), and its chemical structure and affinity studies with h_kit1 and h_kit2 G4 sequences were previously published^31^. Stock solutions (10 mM) of the ligand were prepared in DMSO (Sigma-Aldrich Co., St. Louis, USA).

### Cell culture

The human mast cell leukemia HMC1.2 expressing mutated *KIT* (D816V substitution) was kindly provided by Dr. Joseph Butterfield (Mayo Clinic, Rochester, MN, USA). The canine MCT cell line C2, expressing mutated *KIT* (48 bp internal tandem duplication in the juxtamembrane domain) was kindly provided by Dr. Patrice Dubreuil (Centre de Recherche en Cancérologie de Marseille, France). Cells were cultured in RPMI 1640 medium supplemented with 10% fetal bovine serum (FBS), 2 mM L-glutamine, 1% penicillin/streptomycin (Gibco, Thermo Scientific, Waltham, USA). The C2 culture medium was supplemented with 1 mM sodium pyruvate.

Cell viability was checked by using the Trypan Blue dye exclusion test (Sigma-Aldrich Co., St. Louis, USA). For all the experiments, cells were used from passage 5 to passage 30. Cell cultures were screened routinely for *Mycoplasma spp*. contamination through PCR Mycoplasma Test Kit (PromoKine, Heidelberg, Germany).

For the dose-response assay, 2 × 10^4^ cells were seeded in P96 well plates and treated with a range of doses comprised between 0.2 µM and 10 µM (final concentrations). Cell proliferation was assessed by CellTiter-Blue^®^ Cell Viability Assay (Alamar Blue, Promega, Madison, USA) at T_12_. Fluorescence was measured at 560 nm (excitation wavelength) and 590 nm (emission wavelength), by using a VICTOR^™^X4 Multilabel Plate Reader (Perkin Elmer, Waltham, USA). Three independent experiments were performed, and each concentration was tested six times.

### Cell treatment and qPCR

Cells were seeded in 6-well plates (9 × 10^5^ cells/well) and treated with two doses of AQ1 (1 µM and 2 µM) for 12 hours. For each experiment, we included also untreated and DMSO-treated cells. Five different experiments were conducted for each cell line.

Total RNA was extracted by RNeasy Mini Kit (Qiagen Italia, Milano, Italy). To reduce the possible presence of genomic DNA contamination, a 15-minutes on-column DNase digestion step was included in the RNA isolation protocol. Total RNA concentration was determined using the NanoDrop ND-1000 spectrophotometer (NanoDrop Technologies Inc., Wilmington, USA), and its quality was assessed using the 2100 Bioanalyzer and RNA 6000 Nano kit (Agilent Technologies, Santa Clara, CA, USA). The reverse-transcription of mRNA into cDNA were carried out as previously published^31^.

The list of primers used in the present study for qPCR analysis is reported in Supplementary Table S6.

Target genes to be tested in qPCR cross-validation assay were selected considering different expression trends in both cell lines. Specifically, we selected two genes that were downregulated (*RPTOR* and *HDAC4*) as well as two other ones that were shown to be upregulated (*BMF* and *CDKN1A*) in both cell lines. We also included two DEGs that were downregulated (*EGFR* and *KRS2*) and two genes upregulated (*TNFRSF11b* and *FAXDC2*) exclusively in C2 cell line; finally, two DEGs that were downregulated (CDK6 and MID1) and two genes upregulated (*EGR2* and *CYP1A1*) exclusively in HMC1.2 cell line. Oligonucleotide primers were designed using the software UPL Assay Design Centre web service (Roche Diagnostics, Mannheim, Germany), and each assay specificity was evaluated *in silico*, by using the BLAST tool.

Quantitative real-time PCR reactions (10 µL final volume) were performed as previously reported^28^, using 0.83 ng −1 ng of cDNA and 1–1.25 ng for C2 and HMC1.2, respectively. The analysis was conducted in a LightCycler 480 Instrument (Roche Applied Science, Indianapolis, IN) using standard qPCR conditions (95 °C for 10 min; 45 cycles at 95 °C for 10 s and at 60 °C for 30 s; 40 °C for 30 s). Calibration curves were performed using serial dilutions of a cDNA pool and corresponding values of slope, efficiency (E) and dynamic range are reported in Supplementary Table S7. The assays with an E (%) between 90% and 110% were considered efficient.

The obtained qPCR data were analyzed using the LightCycler480 software release 1.5.0 (Roche Applied Science, Indianapolis, USA) and the second derivative method; the relative quantification (RQ) was calculated with the ^ΔΔ^Ct method^62^. For the normalization step, four internal control genes (ICGs) were used: for C2, the transmembrane BAX inhibitor motif containing 4 (*CGI-119*) and the CCZ1 vacuolar protein trafficking and biogenesis associated homolog (*CCZ1*); for HMC1.2, the glyceraldehyde-3-phosphate dehydrogenase (GAPDH) and the beta-2-microglobulin (ß2M). A cDNA pool was used as calibrator.

### Library preparation and RNA-Seq analysis

Strand-specific RNA-Seq libraries were prepared using the SureSelect strand-specific mRNA library preparation kit (Agilent Technologies, Santa Clara, CA, USA) as per the manufacturer’s instructions. In brief, poly(A) RNA was purified from 1 µg of total RNA using two serial rounds of binding to oligo(dT) magnetic particles; then, fragmented RNA was reverse transcribed to generate cDNA. An Illumina-specific adaptor was sequentially ligated to the 3′ end of cDNA fragments, purified using the AMPure XP beads (Beckman Coulter, Brea, CA, USA), and finally PCR-amplified (13 cycles) using an appropriate indexing primer to allow further samples multiplexing. The PCR-amplified libraries were purified with the AMPure XP beads (Beckman Coulter, Brea, CA, USA) and, then, assessed for their quality and fragments distribution using the 2100 Bioanalyzer DNA 1000 assay (Agilent Technologies, Santa Clara, CA, USA). In the presence of adaptor-dimers (Electropherogram’s peak at 100 to 150-bp), another round of magnetic beads purification was performed. Libraries were quantified using both the Qubit® Fluorometer (Life Technologies, USA) and the qPCR-based NEBNext library quantification kit (New England BioLabs, UK). Finally, equimolar amounts of each ten index-tagged libraries were multiplexed together in one pool (total 4 pools) and then sequenced by an Illumina HiSeq4000 for 50 sequencing cycles. The raw 50 bp single-end sequences (Sanger/Illumina 1.9 encoding) were quality-controlled using FastQC v.0.11.4 (http://www.bioinformatics.babraham.ac.uk/projects/fastqc/), the low-quality bases (quality scores <30) and the adaptor contamination (if present) were removed by Trimmomatic v.0.36^63^ using the parameters ‘LEADING:3 SLIDINGWINDOW:4:20 MINLEN:25′. In the quality control step, we eliminated one biological replicate that showed a non-sufficient output. The high-quality reads were mapped by HISAT2 v.2.0.5^64^ against the Ensembl reference genomes of *Canis lupus familiaris* (CanFam3.1, ftp://ftp.ensembl.org/pub/release-90/fasta/canis_familiaris/dna) or *Homo sapiens* (GRCh38, ftp://ftp.ensembl.org/pub/release-90/fasta/homo_sapiens/dna/). The uniquely-mapped reads aligned to exons were counted with HTSeq v.0.6.1, then tested using the DESeq2 R package v.1.14.1 for the presence of DEGs^65,66^. Genes with a FDR less than 0.05 and a FC value more than 2 were considered as DEGs. The sequencing data associated with this project were deposited in the GenBank’s Gene Expression Omnibus (GEO) under the accession number GSE120272. The statistical analysis of RNA-Seq data was performed considering AQ1 (1 or 2 µM) *versus* DMSO in each cell line. In order to establish the congruency among biological replicates, a PCA and a hierarchical clustering were performed.

## Electronic supplementary material

Supplementary file 1

Supplementary file 2

Supplementary file 3
