# Supplementary material for: Whole-Transcriptome Profiling of Canine and Human in Vitro Models Exposed to a G-Quadruplex Binding Small Molecule
Source: Sci Rep. 2018 Nov 20;8:17107. doi: 10.1038/s41598-018-35516-y (PMC6244004; doi:10.1038/s41598-018-35516-y)
Supplement: Supplementary file 1 [file 41598_2018_35516_MOESM1_ESM.docx]

**WHOLE-TRANSCRIPTOME PROFILING OF CANINE AND HUMAN IN VITRO MODELS EXPOSED TO A G-QUADRUPLEX BINDING SMALL MOLECULE**

Eleonora Zorzan, Ramy Elgendy, Mery Giantin, Mauro Dacasto and Claudia Sissi

LIST OF SUPPLEMENTARY MATERIALS:

**Supplementary file:** Ensemble of figures (S1-S7) , tables (S1-S7), HMC1.2 KEGG analysis (pg. 16-27), C2 KEGG analysis (pg. 28-42).

**Supplementary Dataset S1:** Excel file reporting the whole list of DEGs for both cell lines.

**Supplementary Dataset S2:** Excel file reporting the whole list of common downregulated DEGs for both cell lines.

**Supplementary Figure S1:** **Molecular structure of the G4 ligand used in this study (A) and sequences of the human and canine *KIT* promoter where conserved G4-forming sites have been found (B).**

**
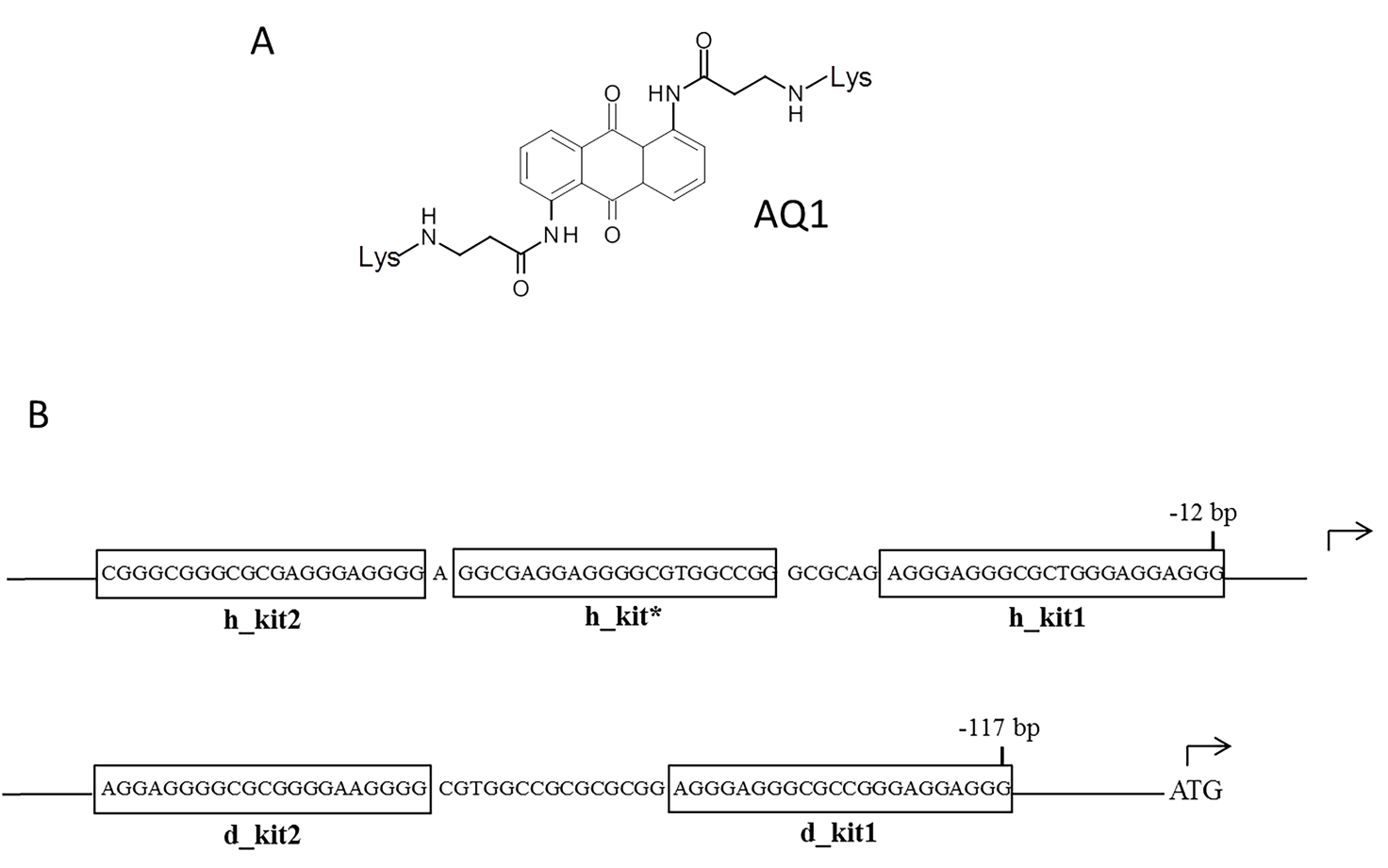
**

**Supplementary Figure S2:** **Effect of the exposure to increasing concentrations of AQ1 on HMC1.2 (A) and C2 (B) cell line proliferation measured by Alamar blue test.** Data referring to the effect of increasing concentrations of AQ1 upon HMC1.2 and C2 cell proliferation after 12 hours of incubation. Data are expressed as the percentage of survival cells (T/mean controls*100). Data points are represented as means ± S.D. of three independent experiments, each one performed in sextuplicate.


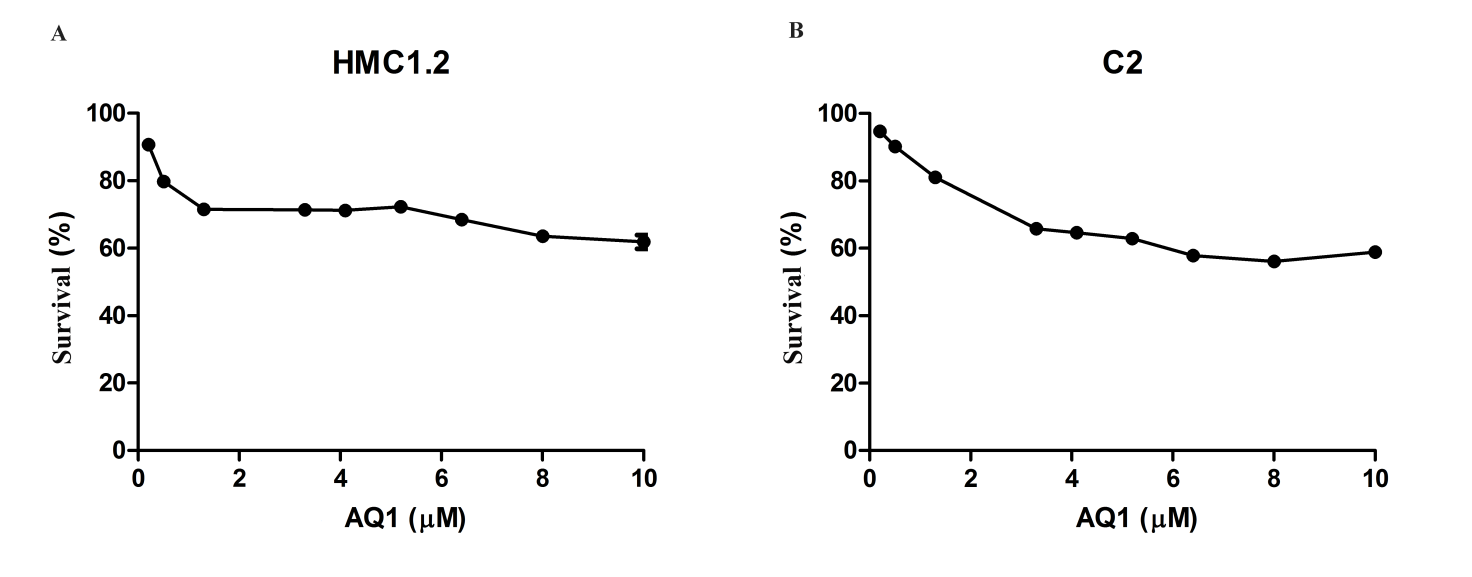


**Supplementary Figure S3:** **Effect of AQ1 on *KIT* and *BCL2*** **mRNA in HMC1.2 and C2 cell lines**. *KIT* and *BCL2* mRNA levels were measured by qPCR, and data (arithmetic means ± S.D.) are expressed as n-fold change (arbitrary units, a.u.) normalized to the RQ of DMSO-treated cells, to which an arbitrary value of 100 was assigned. The one-way ANOVA followed by Bonferroni post-test were used to check for statistical differences between doses and time of treatment.

^**, ***^: p<0.01; p<0.001.


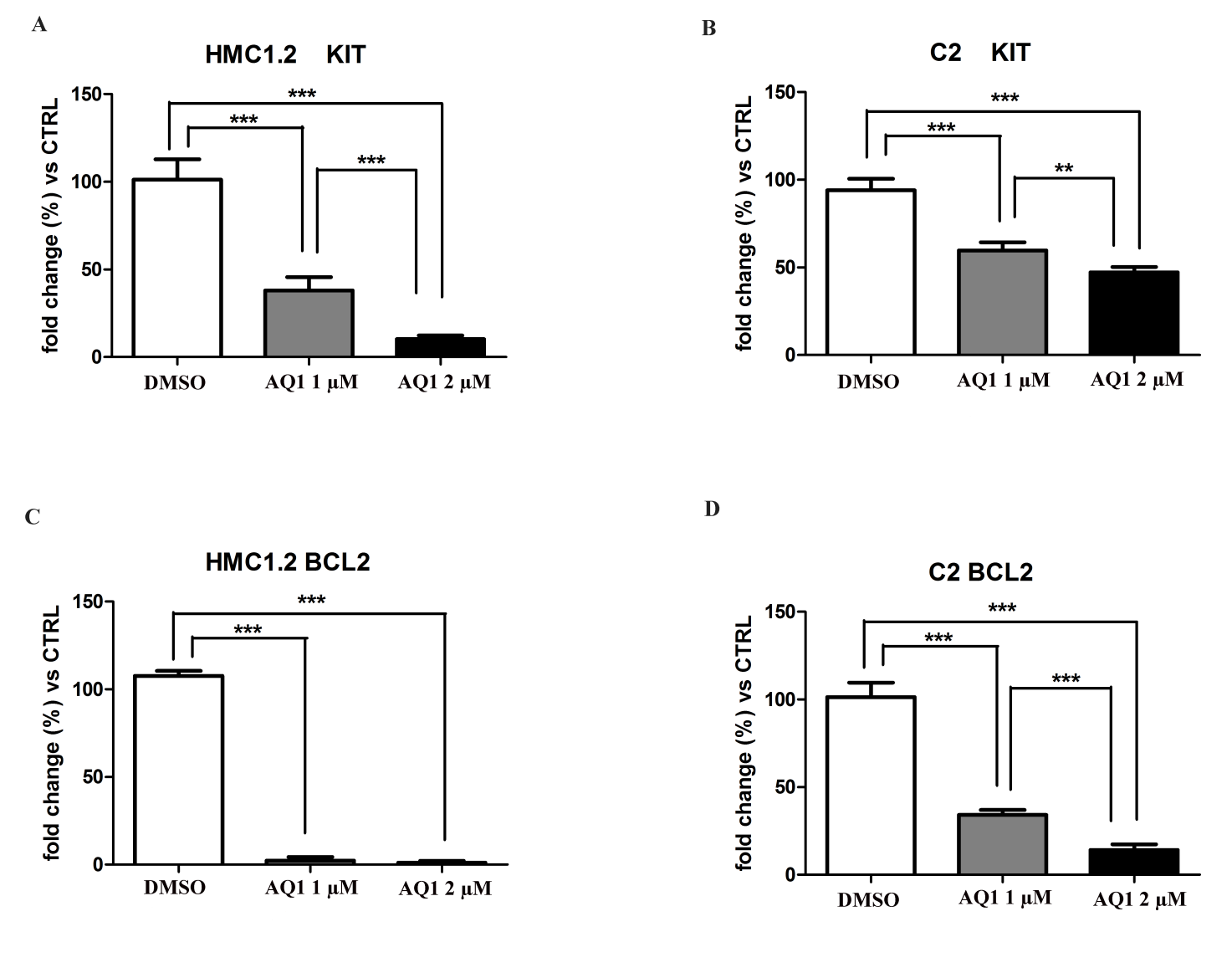


# Supplementary Figure S4. Heatmaps of the top 100 differentially expressed genes. The heatmaps were made using the ClustVis tool (<https://biit.cs.ut.ee/clustvis/>) . (A) HMC1.2 and (B) C2.

#####
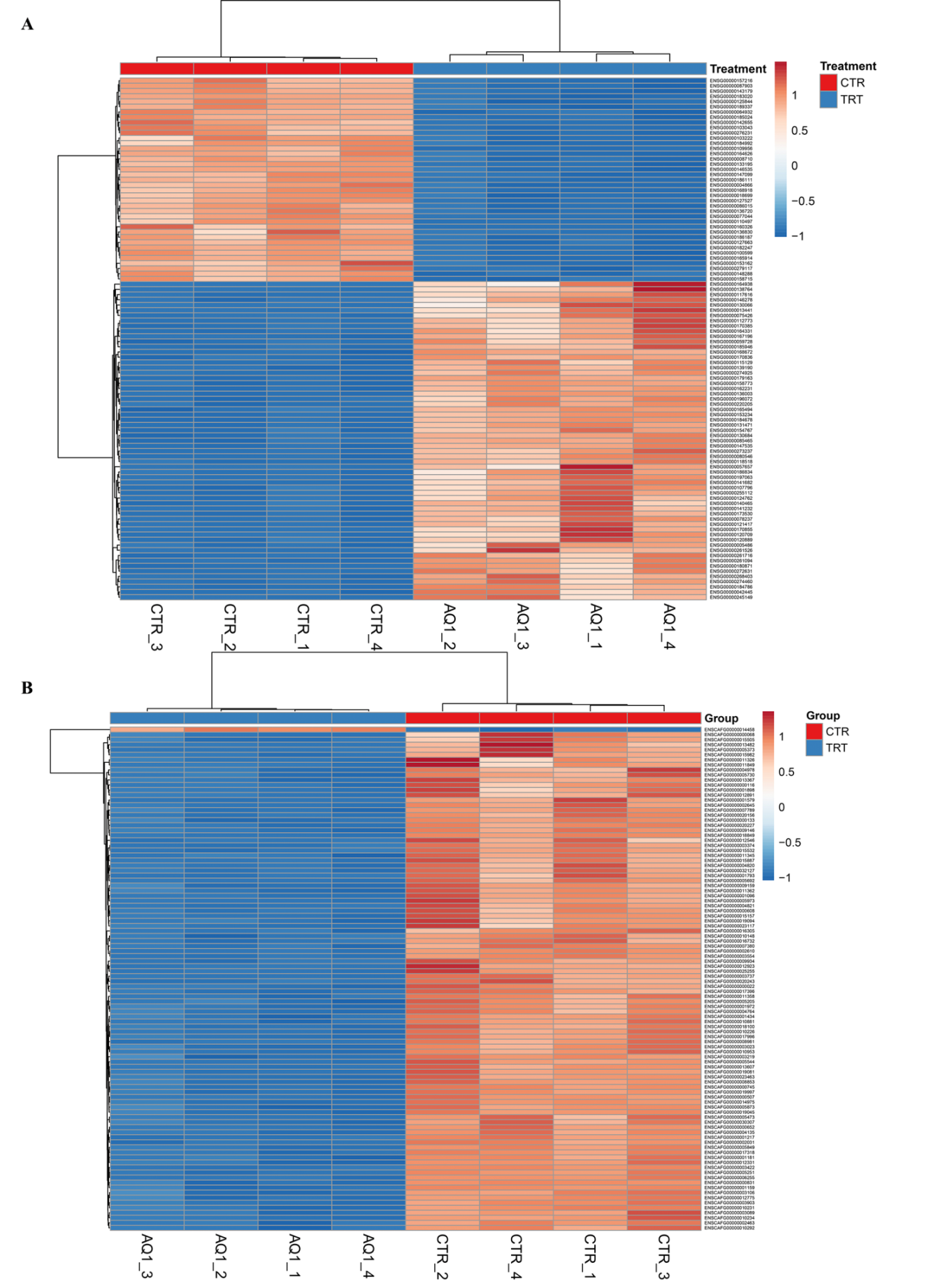


**Supplementary Figure S5:** Cross-validation of RNA-Seq data with qPCR of four genes modulated by AQ1 in HMC1.2 and C2. The mRNA relative quantification (RQ) was calculated by the ^ΔΔ^Ct method (Livak et al., 2001), and normalized using the average of the RQ values of two internal control (reference) genes (*GAPDH* and B2M for human cells, *CCZ1* and *CGI-119* for canine cells). Data (arithmetic means ± S.E.M.) are expressed as n-fold change (arbitrary units, a. u.) normalized to the RQ of vehicle (DMSO) treated cells, to which an arbitrary value of 1 was assigned.


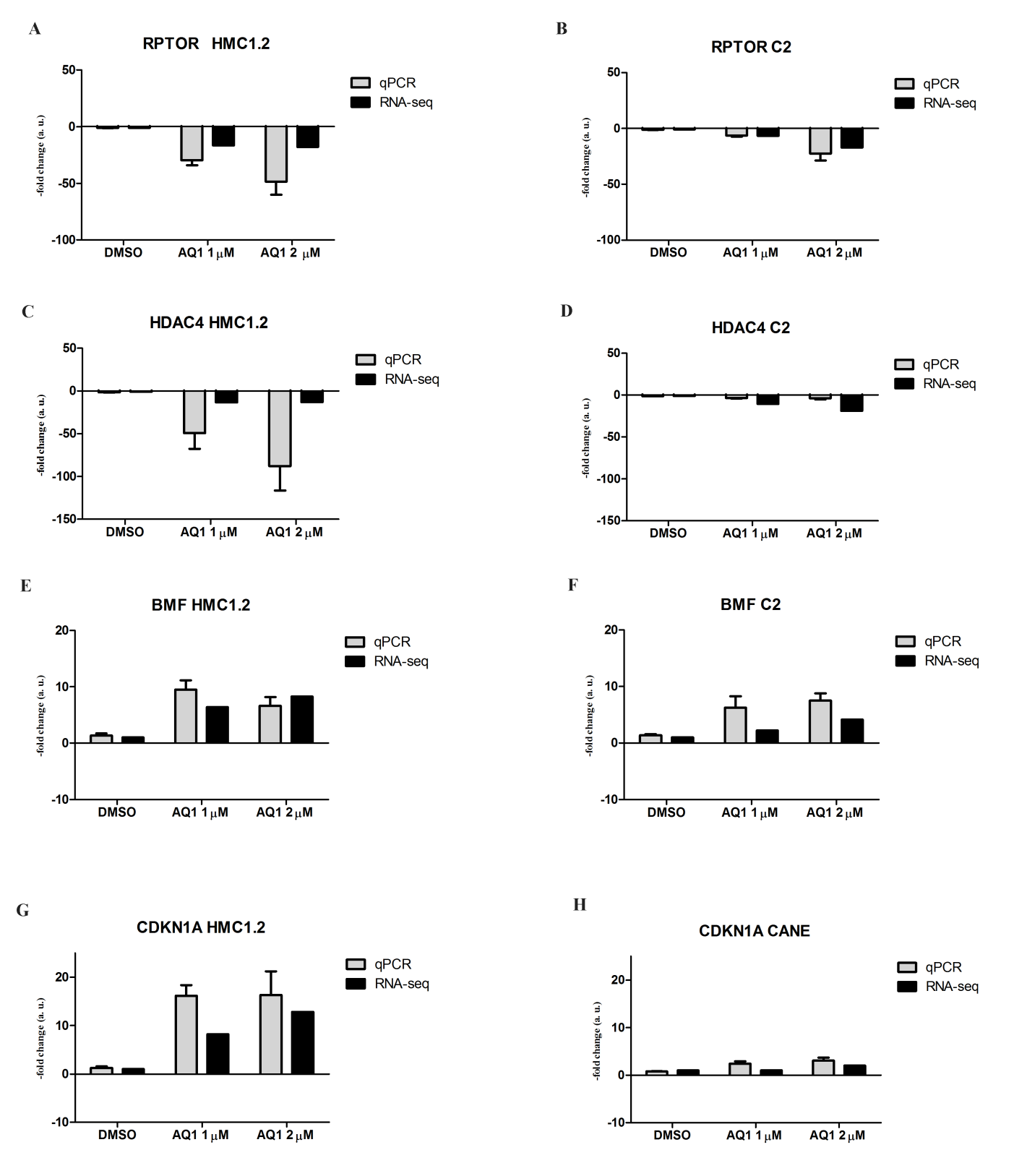


**Supplementary Figure S6:** Cross-validation of RNA-Seq data with qPCR of four genes differentially expressed in HMC1.2 treated with AQ1. The mRNA relative quantification (RQ) was calculated by the ^ΔΔ^Ct method (Livak et al., 2001) , and normalized using the average of the RQ values of two internal control (reference) genes (*GAPDH* and *B2M* for human cells). Data (arithmetic means ± S.E.M.) are expressed as n-fold change (arbitrary units, a. u.) normalized to the RQ of vehicle (DMSO) treated cells, to which an arbitrary value of 1 was assigned.


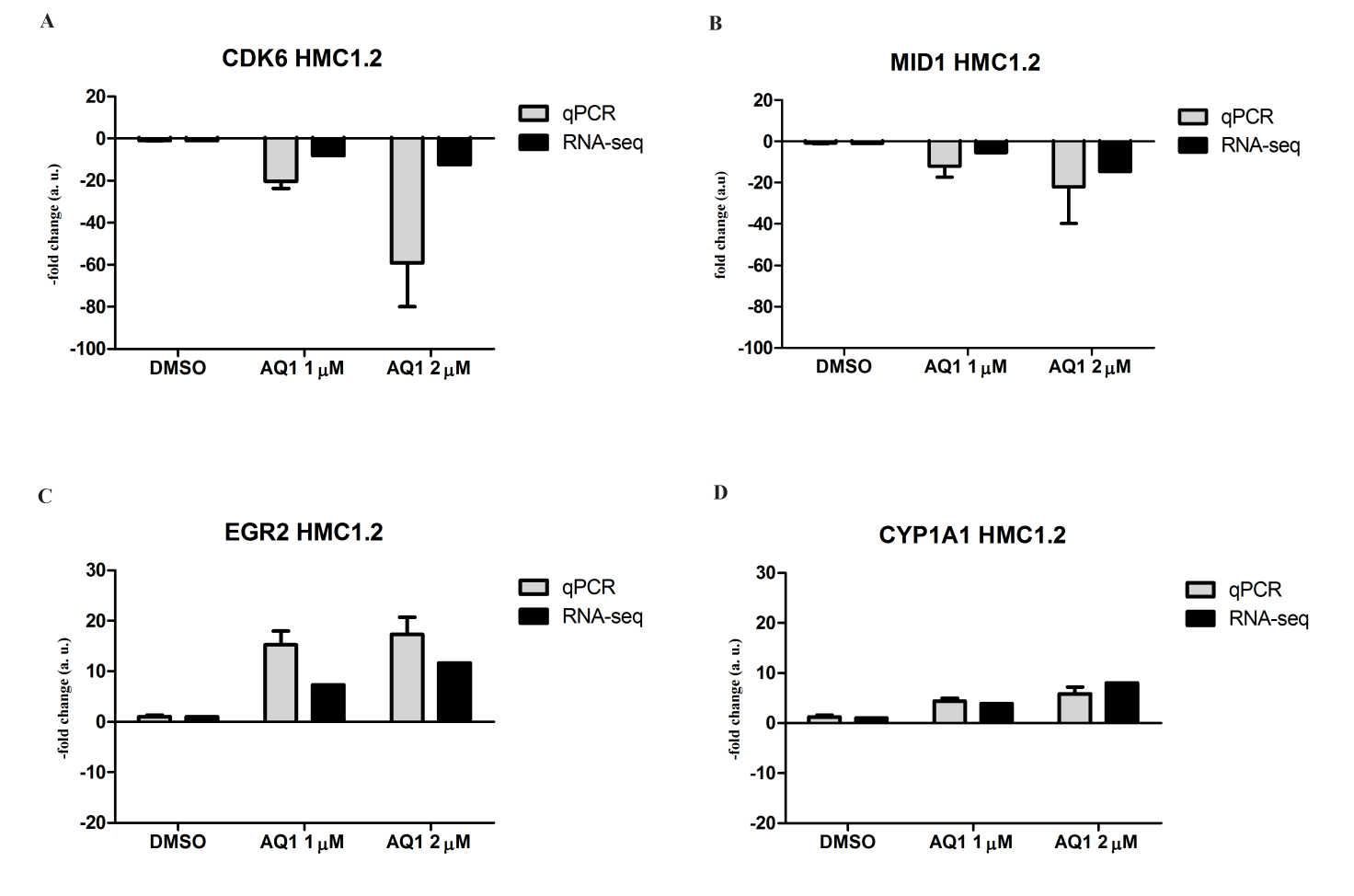


**Supplementary Figure S7:** Cross-validation of RNA-Seq data with qPCR in C2 of four genes differentially expressed in C2 treated with AQ1. The mRNA relative quantification (RQ) was calculated by the ^ΔΔ^Ct method (Livak et al., 2001), and normalized using the average of the RQ values of two internal control (reference) genes (*CCZ1* and *CGI-119*). Data (arithmetic means ± S.E.M.) are expressed as n-fold change (arbitrary units, a. u.) normalized to the RQ of vehicle (DMSO) treated cells, to which an arbitrary value of 1 was assigned.


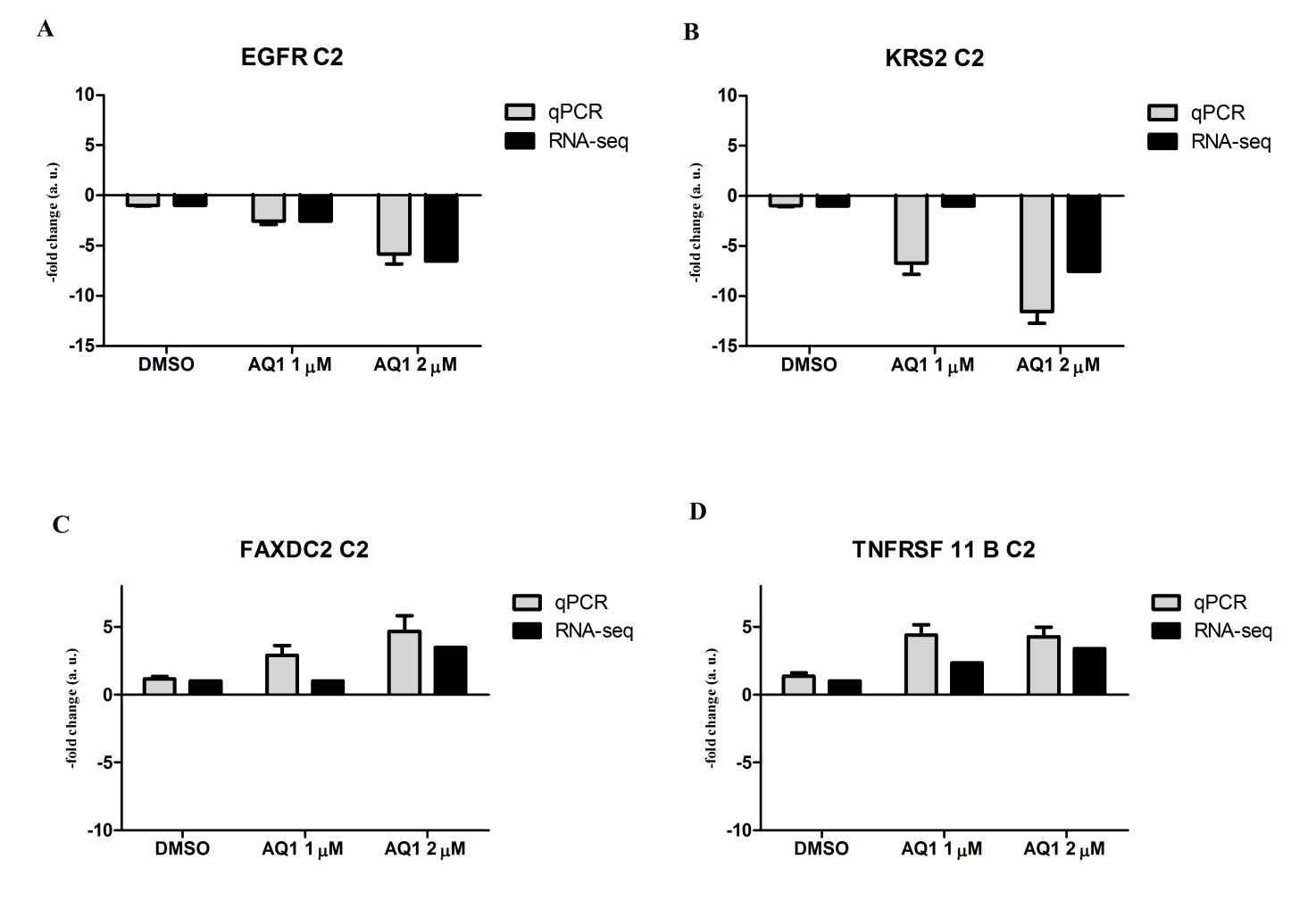


**Supplementary Table S1:** KEGG PATHWAY ANALYSIS - HMC1.2 AQ1 2 µM vs DMSO, FDR<0.05. PATHWAYS NEGATIVELY REGULATED

**Supplementary Table S2:** KEGG PATHWAY ANALYSIS - HMC1.2 AQ1 2 µM vs DMSO, FDR<0.05. PATHWAYS POSITIVELY REGULATED

Kanehisa, M. et al. KEGG: new perspectives on genomes, pathways, diseases and drugs. *Nucleic Acids Res*. **45**, D353-D361 (2017). Kanehisa, M., et al. KEGG as a reference resource for gene and protein annotation. *Nucleic Acids Res.* **44**, D457-D462 (2016). Kanehisa, M. & Goto, S. KEGG: Kyoto Encyclopedia of Genes and Genomes. *Nucleic Acids Res.* **28**, 27-30 (2000).

**Supplementary Table S3:** KEGG PATHWAY ANALYSIS - C2 AQ1 2 µM vs DMSO, FDR<0.05. PATHWAYS NEGATIVELY REGULATED

Kanehisa, M. et al. KEGG: new perspectives on genomes, pathways, diseases and drugs. *Nucleic Acids Res*. **45**, D353-D361 (2017). Kanehisa, M., et al. KEGG as a reference resource for gene and protein annotation. *Nucleic Acids Res.* **44**, D457-D462 (2016). Kanehisa, M. & Goto, S. KEGG: Kyoto Encyclopedia of Genes and Genomes. *Nucleic Acids Res.* **28**, 27-30 (2000).

**Supplementary Table S4:** KEGG PATHWAY ANALYSIS – C2 AQ1 2 µM vs DMSO, FDR<0.05. PATHWAYS POSITIVELY REGULATED

|  |  |  |  |  |  |  |  |  |  |
| --- | --- | --- | --- | --- | --- | --- | --- | --- | --- |

Kanehisa, M. et al. KEGG: new perspectives on genomes, pathways, diseases and drugs. *Nucleic Acids Res*. **45**, D353-D361 (2017). Kanehisa, M., et al. KEGG as a reference resource for gene and protein annotation. *Nucleic Acids Res.* **44**, D457-D462 (2016). Kanehisa, M. & Goto, S. KEGG: Kyoto Encyclopedia of Genes and Genomes. *Nucleic Acids Res.* **28**, 27-30 (2000).

**Supplementary Table S5:** EuQuad output (Quadbase2 tool) related to genes selected for the qPCR confirmation. DEG= differentially expressed gene. O= overlapping G4, NO = non overlapping G4.

| **HUMAN** | | | | | | **GENE SYMBOL** | **DOG** | | | | | |
| --- | --- | --- | --- | --- | --- | --- | --- | --- | --- | --- | --- | --- |
| **Seq. length** | **GC %** | **NO:G4:+** | **NO:G4:-** | **O:G4:+** | **O:G4:-** |  | **Seq. length** | **GC %** | **NO:G4:+** | **NO:G4:-** | **O:G4:+** | **O:G4:-** |
| 4001 | 48.49 | 0 | 2 | 0 | 2 | **RPTOR** | 4001 | 50.39 | 2 | 3 | 9 | 3 |
| 4001 | 59.99 | 2 | 7 | 6 | 20 | **HDAC4** | 4001 | 48.34 | 3 | 6 | 8 | 25 |
| no DEG | | | | | | **EGFR** | 4001 | 48.89 | 0 | 0 | 0 | 0 |
| no DEG | | | | | | **KSR2** | 4001 | 48.19 | 1 | 1 | 4 | 2 |
| 4001 | 50.04 | 1 | 3 | 6 | 14 | **CDK6** | no DEG | | | | | |
| 4001 | 35.94 | 0 | 0 | 0 | 0 | **MID1** | no DEG | | | | | |
| 4001 | 56.64 | 1 | 4 | 2 | 6 | **BMF** | 4001 | 54.84 | 1 | 4 | 2 | 9 |
| 4001 | 48.94 | 0 | 0 | 0 | 0 | **CDKN1A** | 4001 | 64.48 | 1 | 2 | 7 | 8 |
| no DEG | | | | | | **TNFRSF11B** | 4001 | 50.79 | 4 | 0 | 7 | 0 |
| no DEG | | | | | | **FAXDC2** | 4001 | 48.89 | 1 | 0 | 2 | 0 |
| 4001 | 37.69 | 0 | 0 | 0 | 0 | **EGR2** | no DEG | | | | | |
| 4001 | 60.33 | 2 | 2 | 8 | 2 | **CYP1A1** | no DEG | | | | | |

**Supplementary Table S6:** Primers and probes used for the qPCR analysis either obtained from previous publications or specifically designed for this study.

| GENE | HUMAN PRIMER | PROBE | CANINE PRIMER | REFERENCE | PROBE |
| --- | --- | --- | --- | --- | --- |
| B2M | F: AGGCTATCCAGCGTACTCCA  R: TGTCGGATGGATGAAACCCA | #42 |  | Zorzan et al., 2016* |  |
| BCL2 | F: ATGTGTGTGGAGAGCGTCAA  R: GCCGTACAGTTCCACAAAGG |  | F: ACAACGGAGGCTGGGAATG  R: CCTTCAGAGACAGCCAGGAGAA | designed *ex novo* | #110 |
| BMF | F: AGACAAAGCTACCCAGACTCTCA  R: AGCCGATAGCCAGCATTG | #26 | F: TCCCCAAGTCAGGGTGTCAT  R: GGTAGCCAGCGTTGCCATAA | designed *ex novo* | #45 |
| CCZ1 |  |  | F:TGAAGCACTGCATTTAATTGTTTAT  R:CTTCGGCAAAAATCCAATGT | Giantin et al., 2016* | #148 |
| CDK6 | F:CGATGAACTAGGCAAAGACCTACT  R: GGTGGGAATCCAGGTTTTCT | #85 | F: AACAAGGCAAAGACCTCCTTC  R: GATGGGAATCCAAGTTTTCCT | designed *ex novo* | #87 |
| CDKN1A | F: TCACTGTCTTGTACCCTTGTGC  R: CGGCGTTTGGAGTGGTAG | #85 | F: GGACCTGTCGCTGACCTG  R: CGGCGTTTGGAGTGATAGA | designed *ex novo* | #51 |
| CGI-119 |  |  | F:TCTACAATCTAAGAGAGATTTCAGCAA  R:TTCCTGACAAGCACAAAATCC | Aresu et al., 2011* | #15 |
| CYP1A1 | F: CTACAAAACCTTTGAGAAGGGC  R: TCATCTGACAGCTGGACATTG | #2 | F: GCTTCATGCAAAAGATGGTCAAG  R: GTTGTGACTGTGTCAAATCCAGC | designed *ex novo* | #42 |
| EGFR | F: AGCAACAGAGGTGAAAACAGC  R: ACATTCCGGCAAGAGACG | #57 | F: CCACCTGCGTGAAGAAATGC  R: CTTACACTTGCGGACACCATC | designed *ex novo* | #25 |
| EGR2 | F: TTGACCAGATGAACGGAGTG  R: TGGTTTCTAGGTGCAGAGACG | #3 | F: CATCTACCCGGTGGAGGAC  R: TCTTCTCTCCAGTCATGTCAATG | designed *ex novo* | #60 |
| FAXDC2 | F: ACTTCATCTCTCGCTACCGAAT  R: GGTCTCTCCACCATTTGAGG | #137 | F: ACTTCATCTCTCGCTACCGAAT  R: GGACGGGATAGAGGAGAACC | designed *ex novo* | #137 |
| HDAC4 | F: CAGGAGCCCATTGAGAGC  R: GACGCCTGGTAGTTCCTCAG | #67 | F: AGATCCTCATCGCCGAGTT  R: TCCTGCTGATGCTTCATGG | designed *ex novo* | #17 |
| KIT | F: GGCACGGTTGAATGTAAGGC  R: CAGGGTGTGGGGATGGATTT |  | F: CCTTGGAAGTAGTAGATAAAGGATTCA  R: CAGATCCACATTCTGTCCATCA | designed *ex novo* | #60 |
| KRS2 | F: GCTCCTTCTTTGTGGGACAC  R: TCTCTGCGAGGGATCTGC | #8 | F: TGCATCGAAGCAAGTCTCAC  R: TGAGGTTCAATGGTTTGCTTT | designed *ex novo* | #142 |
| GAPDH | F: CTCTGCTCCTCCTGTTCGAC  R: ACGACCAAATCCGTTGACTC | #60 |  | Zorzan et al., 2016* |  |
| MID1 | F: AGACTGCTAAGAATATCACCGAGAG  R: TGAGGTTGATTTCAGGAATTAGAA | #88 | F: TGTAGCTGGAAATGTGTTCATTG  R: TCACTACCCAGTTATTGTGACAGC | designed *ex novo* | #38 |
| RPTOR | F:TGAAGGCTCCAAATCCTTAGC  R:AGAGCTTTCTGAGGACCCATC | #65 | F:TGGGGTCACACTGGATTTG  R:CTGTGAAGATCCAGTTCAGTTCTC | designed *ex novo* | #72 |
| TNFRSF11B | F: GAAGGGCGCTACCTTGAGAT  R: GCAAACTGTATTTCGCTCTGG | #17 | F: TGTGTGCGAGTGTGAGGAA  R: TGCAAACTGTATTTCGTTCTGG | designed *ex novo* | #17 |

* Giantin, M., et al. Transcriptomic analysis identified up-regulation of a solute carrier transporter and UDP glucuronosyltransferases in dogs with aggressive cutaneous mast cell tumours. *Vet J*. **212,** 36-43 (2016).

Aresu, L., et al. Matrix metalloproteinases and their inhibitors in canine mammary tumors. *BMC Vet Res* **7**, 33-43 (2011).

Zorzan, E., et al. Screening of candidate G-quadruplex ligands for the human c-KIT promotorial region and their effects in multiple in-vitro models. Oncotarget, 7, 21658-21675 (2016).

**Supplementary Table S7:** qPCR assay standard curve parameters obtained in HMC1.2 and C2 cell lines.

| **Gene** | **HMC1.2** | | | **C2** | | |
| --- | --- | --- | --- | --- | --- | --- |
|  | **Slope** | **Efficiency (%)** | **Dynamic range (Ct)** | **Slope** | **Efficiency (%)** | **Dynamic range (Ct)** |
| c-KIT | Zorzan et al., 2016 | | | Zorzan et al., submitted manuscript | | |
| BCL2 | Zorzan et al., 2016 | | | Zorzan et al., submitted manuscript | | |
| B2M | Zorzan et al., 2016 | | |  | | |
| GAPDH | Zorzan et al., 2016 | | |  | | |
| CCZ1 |  | | | Zorzan et al., submitted manuscript | | |
| CGI-119 |  | | | Zorzan et al., submitted manuscript | | |
| RPTOR | -3.28 | 101.7 | 23.65-33.33 | -3.49 | 93.3 | 23.60-34.94 |
| HDAC4 | -3.14 | 108 | 30.01-38.04 | -3.26 | 102.9 | 29.37-34.63 |
| EGFR | not expressed in HMC1.2 | | | -3.21 | 104.8 | 26.33-36.00 |
| KRS2 | not expressed in HMC1.2 | | | -3.49 | 93 | 27.66-36.16 |
| CDK6 | -3.12 | 109.1 | 28.66-37.34 | not expressed in C2 | | |
| MID1 | -3.12 | 109 | 29.31-36.87 | not expressed in C2 | | |
| BMF | -3.13 | 108.7 | 25.27-36.32 | -3.26 | 102.6 | 28.65-37.89 |
| TNFRSF11B | not expressed in HMC1.2 | | | -3.36 | 98.6 | 27.41-36.57 |
| EGR2 | -3.21 | 104.7 | 25.77-35.76 | -3.11 | 109.5 | 26.56-34.12 |
| CDKN1A | -3.11 | 109.5 | 26.01-36.52 | -3.51 | 92.7 | 26.71-36.11 |
| FAXDC2 | -3.43 | 95.9 | 31.54-35-72 | -3.09 | 110 | 26.77-32.34 |
| CYP1A1 | -3.14 | 108.3 | 25.63-35.49 | -3.43 | 95.8 | 26.4-38.15 |

# HMC1.2 KEGG ANALYSIS

# A permission for the use of the images was obtained by KEGG (Kanehisa Laboratories rif. 180271).

Kanehisa, M. et al. KEGG: new perspectives on genomes, pathways, diseases and drugs. *Nucleic Acids Res*. **45**, D353-D361 (2017). Kanehisa, M., et al. KEGG as a reference resource for gene and protein annotation. *Nucleic Acids Res.* **44**, D457-D462 (2016). Kanehisa, M. & Goto, S. KEGG: Kyoto Encyclopedia of Genes and Genomes. *Nucleic Acids Res.* **28**, 27-30 (2000).

ENRICHED KEGG PATHWAYS IN HMC1.2 AQ1 2 µM vs DMSO

1. KEGG_PHOSPHATIDYLINOSITOL_SIGNALING_SYSTEM

|  |  |
| --- | --- |
| Dataset | GSEA_Dog_AQ1 2 µMvsDMSO |
| Phenotype | NoPhenotypeAvailable |
| Upregulated in class | na_neg |
| GeneSet | KEGG_PHOSPHATIDYLINOSITOL_SIGNALING_SYSTEM |
| Enrichment Score (ES) | -0.66214985 |
| Normalized Enrichment Score (NES) | -1.7667592 |
| Nominal p-value | 0.0 |
| FDR q-value | 0.0032498706 |
| FWER p-Value | 0.027 |


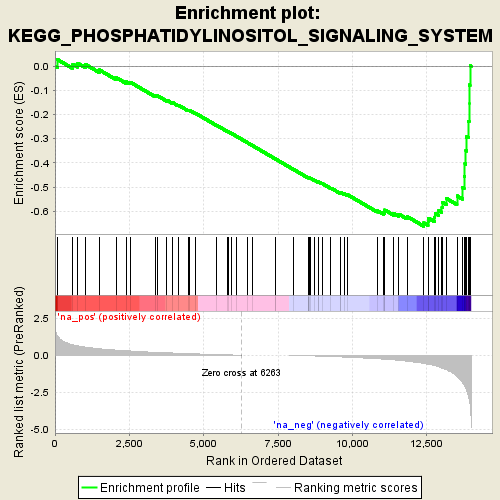


|  | PROBE | RANK IN GENE LIST | RANK METRIC SCORE | RUNNING ES | CORE ENRICHMENT |
| --- | --- | --- | --- | --- | --- |
| 1 | [INPP5J](https://www.affymetrix.com/LinkServlet?probeset=INPP5J) | 83 | 1.310 | 0.0270 | No |
| 2 | [PLCD1](https://www.affymetrix.com/LinkServlet?probeset=PLCD1) | 595 | 0.704 | 0.0080 | No |
| 3 | [INPP1](https://www.affymetrix.com/LinkServlet?probeset=INPP1) | 762 | 0.632 | 0.0119 | No |
| 4 | [PIK3CG](https://www.affymetrix.com/LinkServlet?probeset=PIK3CG) | 1020 | 0.543 | 0.0071 | No |
| 5 | [ITPKA](https://www.affymetrix.com/LinkServlet?probeset=ITPKA) | 1488 | 0.436 | -0.0154 | No |
| 6 | [PIP4K2C](https://www.affymetrix.com/LinkServlet?probeset=PIP4K2C) | 2051 | 0.347 | -0.0471 | No |
| 7 | [PIK3R3](https://www.affymetrix.com/LinkServlet?probeset=PIK3R3) | 2387 | 0.301 | -0.0636 | No |
| 8 | [INPP5E](https://www.affymetrix.com/LinkServlet?probeset=INPP5E) | 2530 | 0.286 | -0.0666 | No |
| 9 | [INPP5K](https://www.affymetrix.com/LinkServlet?probeset=INPP5K) | 3365 | 0.195 | -0.1216 | No |
| 10 | [DGKE](https://www.affymetrix.com/LinkServlet?probeset=DGKE) | 3447 | 0.189 | -0.1226 | No |
| 11 | [IMPA1](https://www.affymetrix.com/LinkServlet?probeset=IMPA1) | 3760 | 0.164 | -0.1409 | No |
| 12 | [PI4KB](https://www.affymetrix.com/LinkServlet?probeset=PI4KB) | 3950 | 0.151 | -0.1507 | No |
| 13 | [SYNJ1](https://www.affymetrix.com/LinkServlet?probeset=SYNJ1) | 4138 | 0.136 | -0.1607 | No |
| 14 | [INPPL1](https://www.affymetrix.com/LinkServlet?probeset=INPPL1) | 4470 | 0.109 | -0.1818 | No |
| 15 | [CALML6](https://www.affymetrix.com/LinkServlet?probeset=CALML6) | 4507 | 0.106 | -0.1817 | No |
| 16 | [DGKZ](https://www.affymetrix.com/LinkServlet?probeset=DGKZ) | 4726 | 0.091 | -0.1950 | No |
| 17 | [PIP5K1C](https://www.affymetrix.com/LinkServlet?probeset=PIP5K1C) | 5423 | 0.045 | -0.2439 | No |
| 18 | [PLCB2](https://www.affymetrix.com/LinkServlet?probeset=PLCB2) | 5802 | 0.030 | -0.2703 | No |
| 19 | [ITPKB](https://www.affymetrix.com/LinkServlet?probeset=ITPKB) | 5821 | 0.028 | -0.2709 | No |
| 20 | [PLCG1](https://www.affymetrix.com/LinkServlet?probeset=PLCG1) | 5917 | 0.020 | -0.2772 | No |
| 21 | [PIK3C2A](https://www.affymetrix.com/LinkServlet?probeset=PIK3C2A) | 6108 | 0.006 | -0.2907 | No |
| 22 | [CALML5](https://www.affymetrix.com/LinkServlet?probeset=CALML5) | 6472 | 0.000 | -0.3167 | No |
| 23 | [DGKB](https://www.affymetrix.com/LinkServlet?probeset=DGKB) | 6649 | 0.000 | -0.3294 | No |
| 24 | [PLCE1](https://www.affymetrix.com/LinkServlet?probeset=PLCE1) | 7404 | 0.000 | -0.3835 | No |
| 25 | [PLCZ1](https://www.affymetrix.com/LinkServlet?probeset=PLCZ1) | 7405 | 0.000 | -0.3835 | No |
| 26 | [ITPR3](https://www.affymetrix.com/LinkServlet?probeset=ITPR3) | 8030 | -0.013 | -0.4280 | No |
| 27 | [PLCB3](https://www.affymetrix.com/LinkServlet?probeset=PLCB3) | 8516 | -0.035 | -0.4620 | No |
| 28 | [OCRL](https://www.affymetrix.com/LinkServlet?probeset=OCRL) | 8519 | -0.035 | -0.4612 | No |
| 29 | [INPP4A](https://www.affymetrix.com/LinkServlet?probeset=INPP4A) | 8534 | -0.036 | -0.4613 | No |
| 30 | [PIP4K2B](https://www.affymetrix.com/LinkServlet?probeset=PIP4K2B) | 8566 | -0.038 | -0.4626 | No |
| 31 | [PIK3R1](https://www.affymetrix.com/LinkServlet?probeset=PIK3R1) | 8597 | -0.040 | -0.4638 | No |
| 32 | [DGKA](https://www.affymetrix.com/LinkServlet?probeset=DGKA) | 8719 | -0.049 | -0.4712 | No |
| 33 | [PLCB4](https://www.affymetrix.com/LinkServlet?probeset=PLCB4) | 8853 | -0.058 | -0.4793 | No |
| 34 | [DGKI](https://www.affymetrix.com/LinkServlet?probeset=DGKI) | 8858 | -0.058 | -0.4781 | No |
| 35 | [PIKFYVE](https://www.affymetrix.com/LinkServlet?probeset=PIKFYVE) | 8980 | -0.067 | -0.4851 | No |
| 36 | [PIK3CA](https://www.affymetrix.com/LinkServlet?probeset=PIK3CA) | 9277 | -0.088 | -0.5042 | No |
| 37 | [INPP5B](https://www.affymetrix.com/LinkServlet?probeset=INPP5B) | 9593 | -0.114 | -0.5239 | No |
| 38 | [DGKQ](https://www.affymetrix.com/LinkServlet?probeset=DGKQ) | 9612 | -0.116 | -0.5223 | No |
| 39 | [PIP5K1A](https://www.affymetrix.com/LinkServlet?probeset=PIP5K1A) | 9715 | -0.125 | -0.5265 | No |
| 40 | [CDS2](https://www.affymetrix.com/LinkServlet?probeset=CDS2) | 9834 | -0.134 | -0.5316 | No |
| 41 | [PLCD3](https://www.affymetrix.com/LinkServlet?probeset=PLCD3) | 10833 | -0.238 | -0.5972 | No |
| 42 | [PI4KA](https://www.affymetrix.com/LinkServlet?probeset=PI4KA) | 11042 | -0.266 | -0.6055 | No |
| 43 | [IPPK](https://www.affymetrix.com/LinkServlet?probeset=IPPK) | 11082 | -0.272 | -0.6015 | No |
| 44 | [PLCD4](https://www.affymetrix.com/LinkServlet?probeset=PLCD4) | 11084 | -0.272 | -0.5947 | No |
| 45 | [PIK3C2B](https://www.affymetrix.com/LinkServlet?probeset=PIK3C2B) | 11391 | -0.317 | -0.6087 | No |
| 46 | [PIK3C2G](https://www.affymetrix.com/LinkServlet?probeset=PIK3C2G) | 11556 | -0.349 | -0.6117 | No |
| 47 | [CDS1](https://www.affymetrix.com/LinkServlet?probeset=CDS1) | 11838 | -0.402 | -0.6218 | No |
| 48 | [SYNJ2](https://www.affymetrix.com/LinkServlet?probeset=SYNJ2) | 12401 | -0.555 | -0.6482 | Yes |
| 49 | [DGKG](https://www.affymetrix.com/LinkServlet?probeset=DGKG) | 12541 | -0.610 | -0.6428 | Yes |
| 50 | [IMPA2](https://www.affymetrix.com/LinkServlet?probeset=IMPA2) | 12551 | -0.615 | -0.6280 | Yes |
| 51 | [PIK3CD](https://www.affymetrix.com/LinkServlet?probeset=PIK3CD) | 12758 | -0.703 | -0.6252 | Yes |
| 52 | [DGKD](https://www.affymetrix.com/LinkServlet?probeset=DGKD) | 12774 | -0.712 | -0.6084 | Yes |
| 53 | [PIK3R5](https://www.affymetrix.com/LinkServlet?probeset=PIK3R5) | 12901 | -0.786 | -0.5976 | Yes |
| 54 | [DGKH](https://www.affymetrix.com/LinkServlet?probeset=DGKH) | 13008 | -0.867 | -0.5835 | Yes |
| 55 | [INPP5D](https://www.affymetrix.com/LinkServlet?probeset=INPP5D) | 13029 | -0.883 | -0.5627 | Yes |
| 56 | [PIK3CB](https://www.affymetrix.com/LinkServlet?probeset=PIK3CB) | 13157 | -0.984 | -0.5471 | Yes |
| 57 | [PLCG2](https://www.affymetrix.com/LinkServlet?probeset=PLCG2) | 13515 | -1.443 | -0.5365 | Yes |
| 58 | [PRKCB](https://www.affymetrix.com/LinkServlet?probeset=PRKCB) | 13702 | -1.865 | -0.5029 | Yes |
| 59 | [ITPK1](https://www.affymetrix.com/LinkServlet?probeset=ITPK1) | 13755 | -2.018 | -0.4559 | Yes |
| 60 | [INPP4B](https://www.affymetrix.com/LinkServlet?probeset=INPP4B) | 13780 | -2.140 | -0.4039 | Yes |
| 61 | [ITPR2](https://www.affymetrix.com/LinkServlet?probeset=ITPR2) | 13812 | -2.237 | -0.3499 | Yes |
| 62 | [ITPR1](https://www.affymetrix.com/LinkServlet?probeset=ITPR1) | 13832 | -2.341 | -0.2924 | Yes |
| 63 | [INPP5A](https://www.affymetrix.com/LinkServlet?probeset=INPP5A) | 13897 | -2.780 | -0.2271 | Yes |
| 64 | [PIP5K1B](https://www.affymetrix.com/LinkServlet?probeset=PIP5K1B) | 13924 | -3.004 | -0.1535 | Yes |
| 65 | [PRKCA](https://www.affymetrix.com/LinkServlet?probeset=PRKCA) | 13927 | -3.026 | -0.0776 | Yes |
| 66 | [PLCB1](https://www.affymetrix.com/LinkServlet?probeset=PLCB1) | 13954 | -3.267 | 0.0027 | Yes |

2. KEGG_ADHERENS_JUNCTION

|  |  |
| --- | --- |
| Dataset | GSEA_Dog_AQ1 2 µMvsDMSO |
| Phenotype | NoPhenotypeAvailable |
| Upregulated in class | na_neg |
| GeneSet | KEGG_ADHERENS_JUNCTION |
| Enrichment Score (ES) | -0.74831843 |
| Normalized Enrichment Score (NES) | -1.9646155 |
| Nominal p-value | 0.0 |
| FDR q-value | 0.0 |
| FWER p-Value | 0.0 |


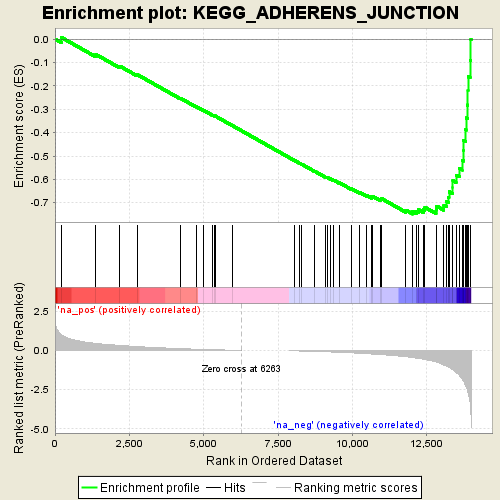


|  | PROBE | RANK IN GENE LIST | RANK METRIC SCORE | RUNNING ES | CORE ENRICHMENT |
| --- | --- | --- | --- | --- | --- |
| 1 | [SNAI2](https://www.affymetrix.com/LinkServlet?probeset=SNAI2) | 214 | 1.007 | 0.0075 | No |
| 2 | [PTPN6](https://www.affymetrix.com/LinkServlet?probeset=PTPN6) | 1369 | 0.459 | -0.0648 | No |
| 3 | [SMAD4](https://www.affymetrix.com/LinkServlet?probeset=SMAD4) | 2181 | 0.329 | -0.1155 | No |
| 4 | [ACTN3](https://www.affymetrix.com/LinkServlet?probeset=ACTN3) | 2764 | 0.259 | -0.1514 | No |
| 5 | [PTPRB](https://www.affymetrix.com/LinkServlet?probeset=PTPRB) | 4216 | 0.130 | -0.2525 | No |
| 6 | [WASL](https://www.affymetrix.com/LinkServlet?probeset=WASL) | 4745 | 0.089 | -0.2884 | No |
| 7 | [BAIAP2](https://www.affymetrix.com/LinkServlet?probeset=BAIAP2) | 5001 | 0.076 | -0.3050 | No |
| 8 | [FGFR1](https://www.affymetrix.com/LinkServlet?probeset=FGFR1) | 5299 | 0.054 | -0.3250 | No |
| 9 | [WASF1](https://www.affymetrix.com/LinkServlet?probeset=WASF1) | 5346 | 0.051 | -0.3272 | No |
| 10 | [WASF2](https://www.affymetrix.com/LinkServlet?probeset=WASF2) | 5378 | 0.049 | -0.3283 | No |
| 11 | [SMAD2](https://www.affymetrix.com/LinkServlet?probeset=SMAD2) | 5972 | 0.015 | -0.3705 | No |
| 12 | [TCF7](https://www.affymetrix.com/LinkServlet?probeset=TCF7) | 8056 | -0.015 | -0.5196 | No |
| 13 | [CTNND1](https://www.affymetrix.com/LinkServlet?probeset=CTNND1) | 8213 | -0.027 | -0.5302 | No |
| 14 | [PTPN1](https://www.affymetrix.com/LinkServlet?probeset=PTPN1) | 8298 | -0.033 | -0.5354 | No |
| 15 | [RAC1](https://www.affymetrix.com/LinkServlet?probeset=RAC1) | 8725 | -0.049 | -0.5649 | No |
| 16 | [MAP3K7](https://www.affymetrix.com/LinkServlet?probeset=MAP3K7) | 9106 | -0.075 | -0.5904 | No |
| 17 | [CSNK2B](https://www.affymetrix.com/LinkServlet?probeset=CSNK2B) | 9146 | -0.077 | -0.5915 | No |
| 18 | [WAS](https://www.affymetrix.com/LinkServlet?probeset=WAS) | 9251 | -0.086 | -0.5970 | No |
| 19 | [EP300](https://www.affymetrix.com/LinkServlet?probeset=EP300) | 9377 | -0.097 | -0.6038 | No |
| 20 | [CREBBP](https://www.affymetrix.com/LinkServlet?probeset=CREBBP) | 9564 | -0.111 | -0.6146 | No |
| 21 | [IQGAP1](https://www.affymetrix.com/LinkServlet?probeset=IQGAP1) | 9955 | -0.145 | -0.6393 | No |
| 22 | [SNAI1](https://www.affymetrix.com/LinkServlet?probeset=SNAI1) | 10248 | -0.173 | -0.6563 | No |
| 23 | [ACTN2](https://www.affymetrix.com/LinkServlet?probeset=ACTN2) | 10480 | -0.198 | -0.6684 | No |
| 24 | [INSR](https://www.affymetrix.com/LinkServlet?probeset=INSR) | 10652 | -0.219 | -0.6756 | No |
| 25 | [TGFBR1](https://www.affymetrix.com/LinkServlet?probeset=TGFBR1) | 10674 | -0.221 | -0.6721 | No |
| 26 | [CSNK2A2](https://www.affymetrix.com/LinkServlet?probeset=CSNK2A2) | 10936 | -0.251 | -0.6852 | No |
| 27 | [RHOA](https://www.affymetrix.com/LinkServlet?probeset=RHOA) | 10980 | -0.257 | -0.6824 | No |
| 28 | [TGFBR2](https://www.affymetrix.com/LinkServlet?probeset=TGFBR2) | 11793 | -0.392 | -0.7317 | No |
| 29 | [RAC2](https://www.affymetrix.com/LinkServlet?probeset=RAC2) | 12025 | -0.448 | -0.7381 | Yes |
| 30 | [ACTN4](https://www.affymetrix.com/LinkServlet?probeset=ACTN4) | 12155 | -0.482 | -0.7364 | Yes |
| 31 | [VCL](https://www.affymetrix.com/LinkServlet?probeset=VCL) | 12218 | -0.498 | -0.7296 | Yes |
| 32 | [SRC](https://www.affymetrix.com/LinkServlet?probeset=SRC) | 12385 | -0.551 | -0.7289 | Yes |
| 33 | [TJP1](https://www.affymetrix.com/LinkServlet?probeset=TJP1) | 12433 | -0.566 | -0.7194 | Yes |
| 34 | [MET](https://www.affymetrix.com/LinkServlet?probeset=MET) | 12810 | -0.728 | -0.7299 | Yes |
| 35 | [LEF1](https://www.affymetrix.com/LinkServlet?probeset=LEF1) | 12831 | -0.742 | -0.7145 | Yes |
| 36 | [FARP2](https://www.affymetrix.com/LinkServlet?probeset=FARP2) | 13072 | -0.917 | -0.7108 | Yes |
| 37 | [ACTN1](https://www.affymetrix.com/LinkServlet?probeset=ACTN1) | 13165 | -0.988 | -0.6950 | Yes |
| 38 | [SMAD3](https://www.affymetrix.com/LinkServlet?probeset=SMAD3) | 13230 | -1.051 | -0.6757 | Yes |
| 39 | [PTPRM](https://www.affymetrix.com/LinkServlet?probeset=PTPRM) | 13246 | -1.070 | -0.6524 | Yes |
| 40 | [PTPRJ](https://www.affymetrix.com/LinkServlet?probeset=PTPRJ) | 13374 | -1.226 | -0.6337 | Yes |
| 41 | [CTNNA1](https://www.affymetrix.com/LinkServlet?probeset=CTNNA1) | 13376 | -1.231 | -0.6058 | Yes |
| 42 | [WASF3](https://www.affymetrix.com/LinkServlet?probeset=WASF3) | 13485 | -1.392 | -0.5819 | Yes |
| 43 | [LMO7](https://www.affymetrix.com/LinkServlet?probeset=LMO7) | 13596 | -1.586 | -0.5537 | Yes |
| 44 | [SSX2IP](https://www.affymetrix.com/LinkServlet?probeset=SSX2IP) | 13686 | -1.830 | -0.5185 | Yes |
| 45 | [TCF7L1](https://www.affymetrix.com/LinkServlet?probeset=TCF7L1) | 13731 | -1.938 | -0.4776 | Yes |
| 46 | [PTPRF](https://www.affymetrix.com/LinkServlet?probeset=PTPRF) | 13737 | -1.952 | -0.4336 | Yes |
| 47 | [SORBS1](https://www.affymetrix.com/LinkServlet?probeset=SORBS1) | 13807 | -2.229 | -0.3879 | Yes |
| 48 | [TCF7L2](https://www.affymetrix.com/LinkServlet?probeset=TCF7L2) | 13821 | -2.291 | -0.3367 | Yes |
| 49 | [FER](https://www.affymetrix.com/LinkServlet?probeset=FER) | 13870 | -2.583 | -0.2815 | Yes |
| 50 | [FYN](https://www.affymetrix.com/LinkServlet?probeset=FYN) | 13883 | -2.691 | -0.2211 | Yes |
| 51 | [EGFR](https://www.affymetrix.com/LinkServlet?probeset=EGFR) | 13886 | -2.708 | -0.1597 | Yes |
| 52 | [IGF1R](https://www.affymetrix.com/LinkServlet?probeset=IGF1R) | 13956 | -3.326 | -0.0891 | Yes |
| 53 | [PARD3](https://www.affymetrix.com/LinkServlet?probeset=PARD3) | 13981 | -4.026 | 0.0007 | Yes |

3. KEGG_FOCAL_ADHESION

|  |  |
| --- | --- |
| Dataset | GSEA_Dog_AQ1 2 µMvsDMSO |
| Phenotype | NoPhenotypeAvailable |
| Upregulated in class | na_neg |
| GeneSet | KEGG_FOCAL_ADHESION |
| Enrichment Score (ES) | -0.62239873 |
| Normalized Enrichment Score (NES) | -1.8083886 |
| Nominal p-value | 0.0 |
| FDR q-value | 9.4507134E-4 |
| FWER p-Value | 0.006 |


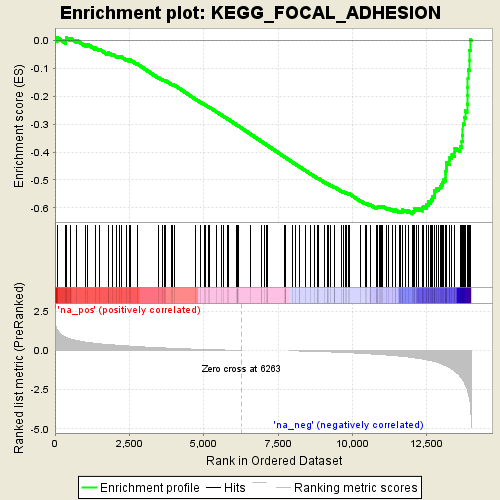


| PROBE | GENE SYMBOL | RANK METRIC SCORE | RUNNING ES | CORE ENRICHMENT |  |
| --- | --- | --- | --- | --- | --- |
| 1 | [ITGA10](https://www.affymetrix.com/LinkServlet?probeset=ITGA10) | 67 | 1.360 | 0.0112 | No |
| 2 | [JUN](https://www.affymetrix.com/LinkServlet?probeset=JUN) | 358 | 0.865 | 0.0005 | No |
| 3 | [COL2A1](https://www.affymetrix.com/LinkServlet?probeset=COL2A1) | 367 | 0.856 | 0.0101 | No |
| 4 | [COMP](https://www.affymetrix.com/LinkServlet?probeset=COMP) | 503 | 0.755 | 0.0092 | No |
| 5 | [FLNC](https://www.affymetrix.com/LinkServlet?probeset=FLNC) | 729 | 0.646 | 0.0006 | No |
| 6 | [PIK3CG](https://www.affymetrix.com/LinkServlet?probeset=PIK3CG) | 1020 | 0.543 | -0.0139 | No |
| 7 | [COL11A2](https://www.affymetrix.com/LinkServlet?probeset=COL11A2) | 1102 | 0.522 | -0.0136 | No |
| 8 | [ITGA3](https://www.affymetrix.com/LinkServlet?probeset=ITGA3) | 1348 | 0.464 | -0.0258 | No |
| 9 | [ARHGAP5](https://www.affymetrix.com/LinkServlet?probeset=ARHGAP5) | 1490 | 0.436 | -0.0309 | No |
| 10 | [SPP1](https://www.affymetrix.com/LinkServlet?probeset=SPP1) | 1779 | 0.385 | -0.0471 | No |
| 11 | [THBS1](https://www.affymetrix.com/LinkServlet?probeset=THBS1) | 1793 | 0.383 | -0.0435 | No |
| 12 | [MYLPF](https://www.affymetrix.com/LinkServlet?probeset=MYLPF) | 1934 | 0.363 | -0.0493 | No |
| 13 | [LAMB2](https://www.affymetrix.com/LinkServlet?probeset=LAMB2) | 2076 | 0.344 | -0.0555 | No |
| 14 | [MYLK](https://www.affymetrix.com/LinkServlet?probeset=MYLK) | 2149 | 0.333 | -0.0567 | No |
| 15 | [ITGA7](https://www.affymetrix.com/LinkServlet?probeset=ITGA7) | 2223 | 0.323 | -0.0582 | No |
| 16 | [PIK3R3](https://www.affymetrix.com/LinkServlet?probeset=PIK3R3) | 2387 | 0.301 | -0.0664 | No |
| 17 | [CRKL](https://www.affymetrix.com/LinkServlet?probeset=CRKL) | 2491 | 0.290 | -0.0704 | No |
| 18 | [CRK](https://www.affymetrix.com/LinkServlet?probeset=CRK) | 2521 | 0.287 | -0.0691 | No |
| 19 | [ACTN3](https://www.affymetrix.com/LinkServlet?probeset=ACTN3) | 2764 | 0.259 | -0.0835 | No |
| 20 | [ITGB7](https://www.affymetrix.com/LinkServlet?probeset=ITGB7) | 3488 | 0.185 | -0.1336 | No |
| 21 | [BIRC2](https://www.affymetrix.com/LinkServlet?probeset=BIRC2) | 3619 | 0.173 | -0.1409 | No |
| 22 | [ZYX](https://www.affymetrix.com/LinkServlet?probeset=ZYX) | 3665 | 0.170 | -0.1422 | No |
| 23 | [ILK](https://www.affymetrix.com/LinkServlet?probeset=ILK) | 3719 | 0.167 | -0.1440 | No |
| 24 | [COL4A1](https://www.affymetrix.com/LinkServlet?probeset=COL4A1) | 3930 | 0.152 | -0.1574 | No |
| 25 | [ITGA1](https://www.affymetrix.com/LinkServlet?probeset=ITGA1) | 3963 | 0.150 | -0.1580 | No |
| 26 | [CCND3](https://www.affymetrix.com/LinkServlet?probeset=CCND3) | 4008 | 0.146 | -0.1594 | No |
| 27 | [MYLK3](https://www.affymetrix.com/LinkServlet?probeset=MYLK3) | 4723 | 0.091 | -0.2099 | No |
| 28 | [LAMC3](https://www.affymetrix.com/LinkServlet?probeset=LAMC3) | 4905 | 0.080 | -0.2221 | No |
| 29 | [SHC3](https://www.affymetrix.com/LinkServlet?probeset=SHC3) | 5022 | 0.074 | -0.2296 | No |
| 30 | [PGF](https://www.affymetrix.com/LinkServlet?probeset=PGF) | 5065 | 0.072 | -0.2317 | No |
| 31 | [ITGA6](https://www.affymetrix.com/LinkServlet?probeset=ITGA6) | 5156 | 0.065 | -0.2375 | No |
| 32 | [BAD](https://www.affymetrix.com/LinkServlet?probeset=BAD) | 5181 | 0.063 | -0.2385 | No |
| 33 | [PIP5K1C](https://www.affymetrix.com/LinkServlet?probeset=PIP5K1C) | 5423 | 0.045 | -0.2553 | No |
| 34 | [PDGFRB](https://www.affymetrix.com/LinkServlet?probeset=PDGFRB) | 5581 | 0.042 | -0.2662 | No |
| 35 | [FN1](https://www.affymetrix.com/LinkServlet?probeset=FN1) | 5659 | 0.042 | -0.2713 | No |
| 36 | [MYL12B](https://www.affymetrix.com/LinkServlet?probeset=MYL12B) | 5797 | 0.030 | -0.2808 | No |
| 37 | [FLNA](https://www.affymetrix.com/LinkServlet?probeset=FLNA) | 5829 | 0.028 | -0.2827 | No |
| 38 | [VTN](https://www.affymetrix.com/LinkServlet?probeset=VTN) | 6089 | 0.007 | -0.3013 | No |
| 39 | [COL3A1](https://www.affymetrix.com/LinkServlet?probeset=COL3A1) | 6125 | 0.005 | -0.3038 | No |
| 40 | [LAMA4](https://www.affymetrix.com/LinkServlet?probeset=LAMA4) | 6128 | 0.005 | -0.3039 | No |
| 41 | [CAPN2](https://www.affymetrix.com/LinkServlet?probeset=CAPN2) | 6134 | 0.005 | -0.3042 | No |
| 42 | [LAMA3](https://www.affymetrix.com/LinkServlet?probeset=LAMA3) | 6152 | 0.005 | -0.3054 | No |
| 43 | [COL11A1](https://www.affymetrix.com/LinkServlet?probeset=COL11A1) | 6582 | 0.000 | -0.3364 | No |
| 44 | [ITGB6](https://www.affymetrix.com/LinkServlet?probeset=ITGB6) | 6952 | 0.000 | -0.3630 | No |
| 45 | [ITGB8](https://www.affymetrix.com/LinkServlet?probeset=ITGB8) | 6953 | 0.000 | -0.3630 | No |
| 46 | [LAMB4](https://www.affymetrix.com/LinkServlet?probeset=LAMB4) | 7025 | 0.000 | -0.3682 | No |
| 47 | [MAPK10](https://www.affymetrix.com/LinkServlet?probeset=MAPK10) | 7092 | 0.000 | -0.3729 | No |
| 48 | [MYL10](https://www.affymetrix.com/LinkServlet?probeset=MYL10) | 7140 | 0.000 | -0.3763 | No |
| 49 | [MYL9](https://www.affymetrix.com/LinkServlet?probeset=MYL9) | 7141 | 0.000 | -0.3763 | No |
| 50 | [THBS2](https://www.affymetrix.com/LinkServlet?probeset=THBS2) | 7714 | 0.000 | -0.4177 | No |
| 51 | [TNC](https://www.affymetrix.com/LinkServlet?probeset=TNC) | 7753 | 0.000 | -0.4204 | No |
| 52 | [TNN](https://www.affymetrix.com/LinkServlet?probeset=TNN) | 7757 | 0.000 | -0.4206 | No |
| 53 | [TNR](https://www.affymetrix.com/LinkServlet?probeset=TNR) | 7760 | 0.000 | -0.4208 | No |
| 54 | [THBS3](https://www.affymetrix.com/LinkServlet?probeset=THBS3) | 7995 | -0.009 | -0.4376 | No |
| 55 | [MYL5](https://www.affymetrix.com/LinkServlet?probeset=MYL5) | 8085 | -0.017 | -0.4438 | No |
| 56 | [MYLK2](https://www.affymetrix.com/LinkServlet?probeset=MYLK2) | 8211 | -0.026 | -0.4525 | No |
| 57 | [SHC1](https://www.affymetrix.com/LinkServlet?probeset=SHC1) | 8217 | -0.027 | -0.4526 | No |
| 58 | [PARVA](https://www.affymetrix.com/LinkServlet?probeset=PARVA) | 8424 | -0.033 | -0.4671 | No |
| 59 | [PIK3R1](https://www.affymetrix.com/LinkServlet?probeset=PIK3R1) | 8597 | -0.040 | -0.4790 | No |
| 60 | [RAC1](https://www.affymetrix.com/LinkServlet?probeset=RAC1) | 8725 | -0.049 | -0.4876 | No |
| 61 | [IBSP](https://www.affymetrix.com/LinkServlet?probeset=IBSP) | 8828 | -0.056 | -0.4943 | No |
| 62 | [ITGA8](https://www.affymetrix.com/LinkServlet?probeset=ITGA8) | 8839 | -0.057 | -0.4944 | No |
| 63 | [COL6A1](https://www.affymetrix.com/LinkServlet?probeset=COL6A1) | 8873 | -0.059 | -0.4961 | No |
| 64 | [RAF1](https://www.affymetrix.com/LinkServlet?probeset=RAF1) | 9056 | -0.072 | -0.5084 | No |
| 65 | [SOS2](https://www.affymetrix.com/LinkServlet?probeset=SOS2) | 9158 | -0.078 | -0.5147 | No |
| 66 | [PARVG](https://www.affymetrix.com/LinkServlet?probeset=PARVG) | 9161 | -0.078 | -0.5140 | No |
| 67 | [SHC4](https://www.affymetrix.com/LinkServlet?probeset=SHC4) | 9198 | -0.081 | -0.5156 | No |
| 68 | [PIK3CA](https://www.affymetrix.com/LinkServlet?probeset=PIK3CA) | 9277 | -0.088 | -0.5202 | No |
| 69 | [FLT4](https://www.affymetrix.com/LinkServlet?probeset=FLT4) | 9380 | -0.097 | -0.5264 | No |
| 70 | [LAMB3](https://www.affymetrix.com/LinkServlet?probeset=LAMB3) | 9392 | -0.098 | -0.5261 | No |
| 71 | [ELK1](https://www.affymetrix.com/LinkServlet?probeset=ELK1) | 9402 | -0.098 | -0.5256 | No |
| 72 | [AKT1](https://www.affymetrix.com/LinkServlet?probeset=AKT1) | 9644 | -0.119 | -0.5416 | No |
| 73 | [PAK1](https://www.affymetrix.com/LinkServlet?probeset=PAK1) | 9689 | -0.123 | -0.5433 | No |
| 74 | [ITGB1](https://www.affymetrix.com/LinkServlet?probeset=ITGB1) | 9690 | -0.123 | -0.5418 | No |
| 75 | [RELN](https://www.affymetrix.com/LinkServlet?probeset=RELN) | 9770 | -0.130 | -0.5460 | No |
| 76 | [COL5A3](https://www.affymetrix.com/LinkServlet?probeset=COL5A3) | 9788 | -0.131 | -0.5457 | No |
| 77 | [RASGRF1](https://www.affymetrix.com/LinkServlet?probeset=RASGRF1) | 9875 | -0.138 | -0.5503 | No |
| 78 | [TLN1](https://www.affymetrix.com/LinkServlet?probeset=TLN1) | 9887 | -0.139 | -0.5494 | No |
| 79 | [ITGA11](https://www.affymetrix.com/LinkServlet?probeset=ITGA11) | 10277 | -0.177 | -0.5755 | No |
| 80 | [SOS1](https://www.affymetrix.com/LinkServlet?probeset=SOS1) | 10425 | -0.192 | -0.5838 | No |
| 81 | [ACTN2](https://www.affymetrix.com/LinkServlet?probeset=ACTN2) | 10480 | -0.198 | -0.5854 | No |
| 82 | [AKT2](https://www.affymetrix.com/LinkServlet?probeset=AKT2) | 10592 | -0.212 | -0.5909 | No |
| 83 | [CHAD](https://www.affymetrix.com/LinkServlet?probeset=CHAD) | 10604 | -0.213 | -0.5891 | No |
| 84 | [HRAS](https://www.affymetrix.com/LinkServlet?probeset=HRAS) | 10793 | -0.233 | -0.6000 | No |
| 85 | [PPP1R12A](https://www.affymetrix.com/LinkServlet?probeset=PPP1R12A) | 10817 | -0.236 | -0.5988 | No |
| 86 | [ITGAV](https://www.affymetrix.com/LinkServlet?probeset=ITGAV) | 10840 | -0.239 | -0.5976 | No |
| 87 | [CCND1](https://www.affymetrix.com/LinkServlet?probeset=CCND1) | 10857 | -0.242 | -0.5959 | No |
| 88 | [ITGB5](https://www.affymetrix.com/LinkServlet?probeset=ITGB5) | 10919 | -0.249 | -0.5974 | No |
| 89 | [COL4A2](https://www.affymetrix.com/LinkServlet?probeset=COL4A2) | 10941 | -0.251 | -0.5959 | No |
| 90 | [RHOA](https://www.affymetrix.com/LinkServlet?probeset=RHOA) | 10980 | -0.257 | -0.5956 | No |
| 91 | [PDPK1](https://www.affymetrix.com/LinkServlet?probeset=PDPK1) | 11021 | -0.263 | -0.5954 | No |
| 92 | [ROCK2](https://www.affymetrix.com/LinkServlet?probeset=ROCK2) | 11148 | -0.280 | -0.6012 | No |
| 93 | [THBS4](https://www.affymetrix.com/LinkServlet?probeset=THBS4) | 11200 | -0.290 | -0.6015 | No |
| 94 | [CCND2](https://www.affymetrix.com/LinkServlet?probeset=CCND2) | 11337 | -0.310 | -0.6076 | No |
| 95 | [MAPK8](https://www.affymetrix.com/LinkServlet?probeset=MAPK8) | 11431 | -0.323 | -0.6105 | No |
| 96 | [RAP1A](https://www.affymetrix.com/LinkServlet?probeset=RAP1A) | 11445 | -0.326 | -0.6076 | No |
| 97 | [EGF](https://www.affymetrix.com/LinkServlet?probeset=EGF) | 11568 | -0.350 | -0.6123 | No |
| 98 | [MAPK9](https://www.affymetrix.com/LinkServlet?probeset=MAPK9) | 11624 | -0.360 | -0.6120 | No |
| 99 | [ROCK1](https://www.affymetrix.com/LinkServlet?probeset=ROCK1) | 11676 | -0.370 | -0.6113 | No |
| 100 | [PPP1CC](https://www.affymetrix.com/LinkServlet?probeset=PPP1CC) | 11681 | -0.371 | -0.6072 | No |
| 101 | [VEGFB](https://www.affymetrix.com/LinkServlet?probeset=VEGFB) | 11783 | -0.390 | -0.6099 | No |
| 102 | [BCAR1](https://www.affymetrix.com/LinkServlet?probeset=BCAR1) | 11870 | -0.409 | -0.6113 | No |
| 103 | [RAC2](https://www.affymetrix.com/LinkServlet?probeset=RAC2) | 12025 | -0.448 | -0.6171 | Yes |
| 104 | [PAK2](https://www.affymetrix.com/LinkServlet?probeset=PAK2) | 12045 | -0.452 | -0.6131 | Yes |
| 105 | [PDGFB](https://www.affymetrix.com/LinkServlet?probeset=PDGFB) | 12072 | -0.462 | -0.6095 | Yes |
| 106 | [MAP2K1](https://www.affymetrix.com/LinkServlet?probeset=MAP2K1) | 12073 | -0.462 | -0.6041 | Yes |
| 107 | [ACTN4](https://www.affymetrix.com/LinkServlet?probeset=ACTN4) | 12155 | -0.482 | -0.6042 | Yes |
| 108 | [VCL](https://www.affymetrix.com/LinkServlet?probeset=VCL) | 12218 | -0.498 | -0.6028 | Yes |
| 109 | [VAV1](https://www.affymetrix.com/LinkServlet?probeset=VAV1) | 12342 | -0.537 | -0.6053 | Yes |
| 110 | [ITGA5](https://www.affymetrix.com/LinkServlet?probeset=ITGA5) | 12355 | -0.542 | -0.5998 | Yes |
| 111 | [SRC](https://www.affymetrix.com/LinkServlet?probeset=SRC) | 12385 | -0.551 | -0.5954 | Yes |
| 112 | [PXN](https://www.affymetrix.com/LinkServlet?probeset=PXN) | 12488 | -0.588 | -0.5958 | Yes |
| 113 | [LAMC1](https://www.affymetrix.com/LinkServlet?probeset=LAMC1) | 12491 | -0.591 | -0.5890 | Yes |
| 114 | [ITGA4](https://www.affymetrix.com/LinkServlet?probeset=ITGA4) | 12545 | -0.611 | -0.5856 | Yes |
| 115 | [GRB2](https://www.affymetrix.com/LinkServlet?probeset=GRB2) | 12547 | -0.612 | -0.5784 | Yes |
| 116 | [COL5A2](https://www.affymetrix.com/LinkServlet?probeset=COL5A2) | 12619 | -0.638 | -0.5760 | Yes |
| 117 | [PAK4](https://www.affymetrix.com/LinkServlet?probeset=PAK4) | 12657 | -0.656 | -0.5709 | Yes |
| 118 | [GSK3B](https://www.affymetrix.com/LinkServlet?probeset=GSK3B) | 12697 | -0.673 | -0.5658 | Yes |
| 119 | [COL4A4](https://www.affymetrix.com/LinkServlet?probeset=COL4A4) | 12699 | -0.674 | -0.5579 | Yes |
| 120 | [PIK3CD](https://www.affymetrix.com/LinkServlet?probeset=PIK3CD) | 12758 | -0.703 | -0.5537 | Yes |
| 121 | [LAMA2](https://www.affymetrix.com/LinkServlet?probeset=LAMA2) | 12762 | -0.704 | -0.5456 | Yes |
| 122 | [LAMB1](https://www.affymetrix.com/LinkServlet?probeset=LAMB1) | 12765 | -0.706 | -0.5374 | Yes |
| 123 | [MET](https://www.affymetrix.com/LinkServlet?probeset=MET) | 12810 | -0.728 | -0.5320 | Yes |
| 124 | [PIK3R5](https://www.affymetrix.com/LinkServlet?probeset=PIK3R5) | 12901 | -0.786 | -0.5292 | Yes |
| 125 | [COL6A6](https://www.affymetrix.com/LinkServlet?probeset=COL6A6) | 12947 | -0.824 | -0.5227 | Yes |
| 126 | [DIAPH1](https://www.affymetrix.com/LinkServlet?probeset=DIAPH1) | 12991 | -0.856 | -0.5157 | Yes |
| 127 | [COL6A3](https://www.affymetrix.com/LinkServlet?probeset=COL6A3) | 13037 | -0.889 | -0.5084 | Yes |
| 128 | [COL4A6](https://www.affymetrix.com/LinkServlet?probeset=COL4A6) | 13071 | -0.917 | -0.5000 | Yes |
| 129 | [FLT1](https://www.affymetrix.com/LinkServlet?probeset=FLT1) | 13128 | -0.961 | -0.4927 | Yes |
| 130 | [ITGA2](https://www.affymetrix.com/LinkServlet?probeset=ITGA2) | 13131 | -0.963 | -0.4814 | Yes |
| 131 | [LAMA1](https://www.affymetrix.com/LinkServlet?probeset=LAMA1) | 13135 | -0.967 | -0.4702 | Yes |
| 132 | [BRAF](https://www.affymetrix.com/LinkServlet?probeset=BRAF) | 13148 | -0.977 | -0.4595 | Yes |
| 133 | [PIK3CB](https://www.affymetrix.com/LinkServlet?probeset=PIK3CB) | 13157 | -0.984 | -0.4485 | Yes |
| 134 | [ACTN1](https://www.affymetrix.com/LinkServlet?probeset=ACTN1) | 13165 | -0.988 | -0.4373 | Yes |
| 135 | [PAK3](https://www.affymetrix.com/LinkServlet?probeset=PAK3) | 13268 | -1.104 | -0.4316 | Yes |
| 136 | [COL5A1](https://www.affymetrix.com/LinkServlet?probeset=COL5A1) | 13274 | -1.109 | -0.4188 | Yes |
| 137 | [VEGFC](https://www.affymetrix.com/LinkServlet?probeset=VEGFC) | 13344 | -1.186 | -0.4098 | Yes |
| 138 | [PDGFD](https://www.affymetrix.com/LinkServlet?probeset=PDGFD) | 13436 | -1.296 | -0.4010 | Yes |
| 139 | [FLNB](https://www.affymetrix.com/LinkServlet?probeset=FLNB) | 13444 | -1.319 | -0.3859 | Yes |
| 140 | [VAV2](https://www.affymetrix.com/LinkServlet?probeset=VAV2) | 13616 | -1.644 | -0.3789 | Yes |
| 141 | [AKT3](https://www.affymetrix.com/LinkServlet?probeset=AKT3) | 13677 | -1.806 | -0.3618 | Yes |
| 142 | [PRKCB](https://www.affymetrix.com/LinkServlet?probeset=PRKCB) | 13702 | -1.865 | -0.3415 | Yes |
| 143 | [RAPGEF1](https://www.affymetrix.com/LinkServlet?probeset=RAPGEF1) | 13715 | -1.895 | -0.3199 | Yes |
| 144 | [PDGFC](https://www.affymetrix.com/LinkServlet?probeset=PDGFC) | 13716 | -1.896 | -0.2975 | Yes |
| 145 | [TLN2](https://www.affymetrix.com/LinkServlet?probeset=TLN2) | 13770 | -2.092 | -0.2766 | Yes |
| 146 | [PTK2](https://www.affymetrix.com/LinkServlet?probeset=PTK2) | 13793 | -2.184 | -0.2524 | Yes |
| 147 | [BCL2](https://www.affymetrix.com/LinkServlet?probeset=BCL2) | 13851 | -2.453 | -0.2275 | Yes |
| 148 | [ITGA9](https://www.affymetrix.com/LinkServlet?probeset=ITGA9) | 13873 | -2.605 | -0.1982 | Yes |
| 149 | [VAV3](https://www.affymetrix.com/LinkServlet?probeset=VAV3) | 13878 | -2.651 | -0.1671 | Yes |
| 150 | [FYN](https://www.affymetrix.com/LinkServlet?probeset=FYN) | 13883 | -2.691 | -0.1356 | Yes |
| 151 | [EGFR](https://www.affymetrix.com/LinkServlet?probeset=EGFR) | 13886 | -2.708 | -0.1037 | Yes |
| 152 | [PRKCA](https://www.affymetrix.com/LinkServlet?probeset=PRKCA) | 13927 | -3.026 | -0.0708 | Yes |
| 153 | [DOCK1](https://www.affymetrix.com/LinkServlet?probeset=DOCK1) | 13931 | -3.041 | -0.0351 | Yes |
| 154 | [IGF1R](https://www.affymetrix.com/LinkServlet?probeset=IGF1R) | 13956 | -3.326 | 0.0025 | Yes |

4. KEGG_COMPLEMENT_AND_COAGULATION_CASCADES

|  |  |
| --- | --- |
| Dataset | GSEA_Dog_AQ1 2 µMvsDMSO |
| Phenotype | NoPhenotypeAvailable |
| Upregulated in class | na_pos |
| GeneSet | KEGG_COMPLEMENT_AND_COAGULATION_CASCADES |
| Enrichment Score (ES) | 0.5901233 |
| Normalized Enrichment Score (NES) | 1.9275452 |
| Nominal p-value | 0.0 |
| FDR q-value | 0.010780026 |
| FWER p-Value | 0.009 |


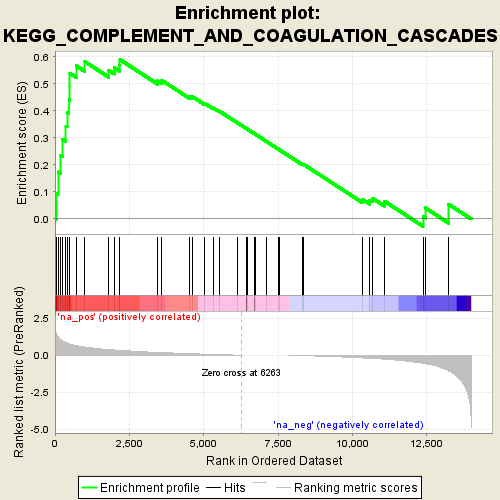


|  | PROBE | RANK IN GENE LIST | RANK METRIC SCORE | RUNNING ES | CORE ENRICHMENT |
| --- | --- | --- | --- | --- | --- |
| 1 | [F11](https://www.affymetrix.com/LinkServlet?probeset=F11) | 38 | 1.495 | 0.0957 | Yes |
| 2 | [C5AR1](https://www.affymetrix.com/LinkServlet?probeset=C5AR1) | 99 | 1.257 | 0.1741 | Yes |
| 3 | [SERPINE1](https://www.affymetrix.com/LinkServlet?probeset=SERPINE1) | 194 | 1.041 | 0.2359 | Yes |
| 4 | [C4BPB](https://www.affymetrix.com/LinkServlet?probeset=C4BPB) | 243 | 0.964 | 0.2958 | Yes |
| 5 | [C3AR1](https://www.affymetrix.com/LinkServlet?probeset=C3AR1) | 366 | 0.857 | 0.3435 | Yes |
| 6 | [SERPINA1](https://www.affymetrix.com/LinkServlet?probeset=SERPINA1) | 403 | 0.829 | 0.3954 | Yes |
| 7 | [F2R](https://www.affymetrix.com/LinkServlet?probeset=F2R) | 468 | 0.778 | 0.4420 | Yes |
| 8 | [SERPINC1](https://www.affymetrix.com/LinkServlet?probeset=SERPINC1) | 499 | 0.756 | 0.4896 | Yes |
| 9 | [CD55](https://www.affymetrix.com/LinkServlet?probeset=CD55) | 500 | 0.756 | 0.5394 | Yes |
| 10 | [PLAUR](https://www.affymetrix.com/LinkServlet?probeset=PLAUR) | 708 | 0.652 | 0.5674 | Yes |
| 11 | [C8G](https://www.affymetrix.com/LinkServlet?probeset=C8G) | 1000 | 0.551 | 0.5828 | Yes |
| 12 | [F2](https://www.affymetrix.com/LinkServlet?probeset=F2) | 1804 | 0.382 | 0.5503 | Yes |
| 13 | [CFI](https://www.affymetrix.com/LinkServlet?probeset=CFI) | 1993 | 0.355 | 0.5602 | Yes |
| 14 | [PROS1](https://www.affymetrix.com/LinkServlet?probeset=PROS1) | 2172 | 0.329 | 0.5691 | Yes |
| 15 | [C1R](https://www.affymetrix.com/LinkServlet?probeset=C1R) | 2182 | 0.329 | 0.5901 | Yes |
| 16 | [CFD](https://www.affymetrix.com/LinkServlet?probeset=CFD) | 3453 | 0.188 | 0.5114 | No |
| 17 | [C3](https://www.affymetrix.com/LinkServlet?probeset=C3) | 3590 | 0.176 | 0.5132 | No |
| 18 | [C1S](https://www.affymetrix.com/LinkServlet?probeset=C1S) | 4529 | 0.105 | 0.4529 | No |
| 19 | [F5](https://www.affymetrix.com/LinkServlet?probeset=F5) | 4607 | 0.099 | 0.4538 | No |
| 20 | [CD59](https://www.affymetrix.com/LinkServlet?probeset=CD59) | 5033 | 0.074 | 0.4282 | No |
| 21 | [SERPING1](https://www.affymetrix.com/LinkServlet?probeset=SERPING1) | 5322 | 0.053 | 0.4111 | No |
| 22 | [FGB](https://www.affymetrix.com/LinkServlet?probeset=FGB) | 5518 | 0.042 | 0.3999 | No |
| 23 | [A2M](https://www.affymetrix.com/LinkServlet?probeset=A2M) | 6130 | 0.005 | 0.3564 | No |
| 24 | [C1QB](https://www.affymetrix.com/LinkServlet?probeset=C1QB) | 6424 | 0.000 | 0.3354 | No |
| 25 | [C1QC](https://www.affymetrix.com/LinkServlet?probeset=C1QC) | 6425 | 0.000 | 0.3354 | No |
| 26 | [C4BPA](https://www.affymetrix.com/LinkServlet?probeset=C4BPA) | 6436 | 0.000 | 0.3347 | No |
| 27 | [C5](https://www.affymetrix.com/LinkServlet?probeset=C5) | 6439 | 0.000 | 0.3345 | No |
| 28 | [C8A](https://www.affymetrix.com/LinkServlet?probeset=C8A) | 6448 | 0.000 | 0.3340 | No |
| 29 | [C8B](https://www.affymetrix.com/LinkServlet?probeset=C8B) | 6449 | 0.000 | 0.3340 | No |
| 30 | [C9](https://www.affymetrix.com/LinkServlet?probeset=C9) | 6453 | 0.000 | 0.3337 | No |
| 31 | [F10](https://www.affymetrix.com/LinkServlet?probeset=F10) | 6699 | 0.000 | 0.3162 | No |
| 32 | [F13A1](https://www.affymetrix.com/LinkServlet?probeset=F13A1) | 6700 | 0.000 | 0.3162 | No |
| 33 | [F13B](https://www.affymetrix.com/LinkServlet?probeset=F13B) | 6701 | 0.000 | 0.3162 | No |
| 34 | [F7](https://www.affymetrix.com/LinkServlet?probeset=F7) | 6703 | 0.000 | 0.3161 | No |
| 35 | [FGA](https://www.affymetrix.com/LinkServlet?probeset=FGA) | 6742 | 0.000 | 0.3134 | No |
| 36 | [MASP1](https://www.affymetrix.com/LinkServlet?probeset=MASP1) | 7095 | 0.000 | 0.2881 | No |
| 37 | [SERPINA5](https://www.affymetrix.com/LinkServlet?probeset=SERPINA5) | 7528 | 0.000 | 0.2572 | No |
| 38 | [SERPIND1](https://www.affymetrix.com/LinkServlet?probeset=SERPIND1) | 7536 | 0.000 | 0.2567 | No |
| 39 | [C1QA](https://www.affymetrix.com/LinkServlet?probeset=C1QA) | 8325 | -0.033 | 0.2023 | No |
| 40 | [FGG](https://www.affymetrix.com/LinkServlet?probeset=FGG) | 8357 | -0.033 | 0.2023 | No |
| 41 | [SERPINF2](https://www.affymetrix.com/LinkServlet?probeset=SERPINF2) | 10322 | -0.183 | 0.0735 | No |
| 42 | [MASP2](https://www.affymetrix.com/LinkServlet?probeset=MASP2) | 10578 | -0.210 | 0.0691 | No |
| 43 | [C7](https://www.affymetrix.com/LinkServlet?probeset=C7) | 10665 | -0.220 | 0.0774 | No |
| 44 | [C6](https://www.affymetrix.com/LinkServlet?probeset=C6) | 11078 | -0.271 | 0.0657 | No |
| 45 | [PLAU](https://www.affymetrix.com/LinkServlet?probeset=PLAU) | 12372 | -0.546 | 0.0089 | No |
| 46 | [CPB2](https://www.affymetrix.com/LinkServlet?probeset=CPB2) | 12447 | -0.571 | 0.0412 | No |
| 47 | [F8](https://www.affymetrix.com/LinkServlet?probeset=F8) | 13235 | -1.056 | 0.0542 | No |

5. KEGG_P53_SIGNALING_PATHWAY

|  |  |
| --- | --- |
| Dataset | GSEA_Dog_AQ1 2 µMvsDMSO |
| Phenotype | NoPhenotypeAvailable |
| Upregulated in class | na_pos |
| GeneSet | KEGG_P53_SIGNALING_PATHWAY |
| Enrichment Score (ES) | 0.37832382 |
| Normalized Enrichment Score (NES) | 1.219188 |
| Nominal p-value | 0.12571429 |
| FDR q-value | 0.48476326 |
| FWER p-Value | 0.996 |


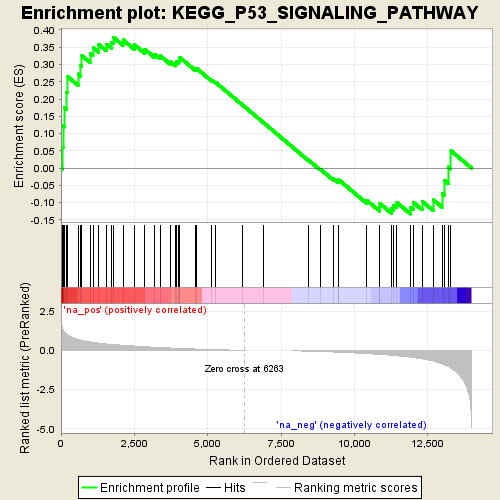


|  | PROBE | RANK IN GENE LIST | RANK METRIC SCORE | RUNNING ES | CORE ENRICHMENT |
| --- | --- | --- | --- | --- | --- |
| 1 | [CCNG2](https://www.affymetrix.com/LinkServlet?probeset=CCNG2) | 64 | 1.364 | 0.0596 | Yes |
| 2 | [SESN3](https://www.affymetrix.com/LinkServlet?probeset=SESN3) | 69 | 1.354 | 0.1230 | Yes |
| 3 | [DDB2](https://www.affymetrix.com/LinkServlet?probeset=DDB2) | 126 | 1.186 | 0.1748 | Yes |
| 4 | [SERPINE1](https://www.affymetrix.com/LinkServlet?probeset=SERPINE1) | 194 | 1.041 | 0.2190 | Yes |
| 5 | [CDKN1A](https://www.affymetrix.com/LinkServlet?probeset=CDKN1A) | 210 | 1.018 | 0.2658 | Yes |
| 6 | [CCNE2](https://www.affymetrix.com/LinkServlet?probeset=CCNE2) | 583 | 0.711 | 0.2726 | Yes |
| 7 | [GADD45B](https://www.affymetrix.com/LinkServlet?probeset=GADD45B) | 674 | 0.666 | 0.2975 | Yes |
| 8 | [GADD45A](https://www.affymetrix.com/LinkServlet?probeset=GADD45A) | 706 | 0.652 | 0.3260 | Yes |
| 9 | [ZMAT3](https://www.affymetrix.com/LinkServlet?probeset=ZMAT3) | 991 | 0.555 | 0.3317 | Yes |
| 10 | [SFN](https://www.affymetrix.com/LinkServlet?probeset=SFN) | 1089 | 0.525 | 0.3494 | Yes |
| 11 | [SESN2](https://www.affymetrix.com/LinkServlet?probeset=SESN2) | 1282 | 0.479 | 0.3582 | Yes |
| 12 | [TP53I3](https://www.affymetrix.com/LinkServlet?probeset=TP53I3) | 1548 | 0.425 | 0.3592 | Yes |
| 13 | [SIAH1](https://www.affymetrix.com/LinkServlet?probeset=SIAH1) | 1727 | 0.395 | 0.3650 | Yes |
| 14 | [THBS1](https://www.affymetrix.com/LinkServlet?probeset=THBS1) | 1793 | 0.383 | 0.3783 | Yes |
| 15 | [RRM2](https://www.affymetrix.com/LinkServlet?probeset=RRM2) | 2113 | 0.339 | 0.3714 | No |
| 16 | [SESN1](https://www.affymetrix.com/LinkServlet?probeset=SESN1) | 2502 | 0.289 | 0.3572 | No |
| 17 | [CDK2](https://www.affymetrix.com/LinkServlet?probeset=CDK2) | 2850 | 0.250 | 0.3440 | No |
| 18 | [RRM2B](https://www.affymetrix.com/LinkServlet?probeset=RRM2B) | 3196 | 0.211 | 0.3292 | No |
| 19 | [BAX](https://www.affymetrix.com/LinkServlet?probeset=BAX) | 3378 | 0.194 | 0.3254 | No |
| 20 | [PPM1D](https://www.affymetrix.com/LinkServlet?probeset=PPM1D) | 3725 | 0.166 | 0.3084 | No |
| 21 | [RCHY1](https://www.affymetrix.com/LinkServlet?probeset=RCHY1) | 3904 | 0.153 | 0.3028 | No |
| 22 | [CDK1](https://www.affymetrix.com/LinkServlet?probeset=CDK1) | 3933 | 0.152 | 0.3080 | No |
| 23 | [CCND3](https://www.affymetrix.com/LinkServlet?probeset=CCND3) | 4008 | 0.146 | 0.3096 | No |
| 24 | [CHEK2](https://www.affymetrix.com/LinkServlet?probeset=CHEK2) | 4041 | 0.144 | 0.3140 | No |
| 25 | [SERPINB5](https://www.affymetrix.com/LinkServlet?probeset=SERPINB5) | 4051 | 0.144 | 0.3202 | No |
| 26 | [CCNE1](https://www.affymetrix.com/LinkServlet?probeset=CCNE1) | 4569 | 0.102 | 0.2879 | No |
| 27 | [EI24](https://www.affymetrix.com/LinkServlet?probeset=EI24) | 4630 | 0.097 | 0.2881 | No |
| 28 | [CDK4](https://www.affymetrix.com/LinkServlet?probeset=CDK4) | 5140 | 0.066 | 0.2547 | No |
| 29 | [CCNG1](https://www.affymetrix.com/LinkServlet?probeset=CCNG1) | 5258 | 0.058 | 0.2491 | No |
| 30 | [FAS](https://www.affymetrix.com/LinkServlet?probeset=FAS) | 6195 | 0.004 | 0.1821 | No |
| 31 | [IGFBP3](https://www.affymetrix.com/LinkServlet?probeset=IGFBP3) | 6916 | 0.000 | 0.1305 | No |
| 32 | [PERP](https://www.affymetrix.com/LinkServlet?probeset=PERP) | 8428 | -0.033 | 0.0237 | No |
| 33 | [CD82](https://www.affymetrix.com/LinkServlet?probeset=CD82) | 8833 | -0.057 | -0.0026 | No |
| 34 | [GTSE1](https://www.affymetrix.com/LinkServlet?probeset=GTSE1) | 9290 | -0.089 | -0.0311 | No |
| 35 | [MDM4](https://www.affymetrix.com/LinkServlet?probeset=MDM4) | 9448 | -0.102 | -0.0376 | No |
| 36 | [TSC2](https://www.affymetrix.com/LinkServlet?probeset=TSC2) | 9469 | -0.104 | -0.0341 | No |
| 37 | [ATM](https://www.affymetrix.com/LinkServlet?probeset=ATM) | 10414 | -0.192 | -0.0928 | No |
| 38 | [CCND1](https://www.affymetrix.com/LinkServlet?probeset=CCND1) | 10857 | -0.242 | -0.1131 | No |
| 39 | [CHEK1](https://www.affymetrix.com/LinkServlet?probeset=CHEK1) | 10861 | -0.242 | -0.1019 | No |
| 40 | [MDM2](https://www.affymetrix.com/LinkServlet?probeset=MDM2) | 11261 | -0.299 | -0.1165 | No |
| 41 | [CCND2](https://www.affymetrix.com/LinkServlet?probeset=CCND2) | 11337 | -0.310 | -0.1073 | No |
| 42 | [CASP9](https://www.affymetrix.com/LinkServlet?probeset=CASP9) | 11448 | -0.326 | -0.0999 | No |
| 43 | [APAF1](https://www.affymetrix.com/LinkServlet?probeset=APAF1) | 11911 | -0.419 | -0.1133 | No |
| 44 | [ATR](https://www.affymetrix.com/LinkServlet?probeset=ATR) | 12009 | -0.443 | -0.0994 | No |
| 45 | [STEAP3](https://www.affymetrix.com/LinkServlet?probeset=STEAP3) | 12311 | -0.526 | -0.0962 | No |
| 46 | [GADD45G](https://www.affymetrix.com/LinkServlet?probeset=GADD45G) | 12690 | -0.671 | -0.0918 | No |
| 47 | [CDK6](https://www.affymetrix.com/LinkServlet?probeset=CDK6) | 12987 | -0.851 | -0.0730 | No |
| 48 | [CCNB3](https://www.affymetrix.com/LinkServlet?probeset=CCNB3) | 13080 | -0.926 | -0.0360 | No |
| 49 | [RFWD2](https://www.affymetrix.com/LinkServlet?probeset=RFWD2) | 13207 | -1.027 | 0.0033 | No |
| 50 | [TP73](https://www.affymetrix.com/LinkServlet?probeset=TP73) | 13292 | -1.124 | 0.0501 | No |

**C2 KEGG PATHWAYS ANALYSIS**

# A permission for the use of the images was obtained by KEGG (Kanehisa Laboratories rif. 180271).

Kanehisa, M. et al. KEGG: new perspectives on genomes, pathways, diseases and drugs. *Nucleic Acids Res*. **45**, D353-D361 (2017). Kanehisa, M., et al. KEGG as a reference resource for gene and protein annotation. *Nucleic Acids Res.* **44**, D457-D462 (2016). Kanehisa, M. & Goto, S. KEGG: Kyoto Encyclopedia of Genes and Genomes. *Nucleic Acids Res.* **28**, 27-30 (2000).

ENRICHED KEGG PATHWAYS IN C2 AQ1 2 µM vs DMSO

1. KEGG_DNA_REPLICATION

| Dataset | GSEA_Human_AQ1 2 µM vs DMSO |
| --- | --- |
| Phenotype | NoPhenotypeAvailable |
| Upregulated in class | na_neg |
| GeneSet | KEGG_DNA_REPLICATION |
| Enrichment Score (ES) | -0.77317685 |
| Normalized Enrichment Score (NES) | -1.7819222 |
| Nominal p-value | 0.0 |
| FDR q-value | 0.007581893 |
| FWER p-Value | 0.012 |


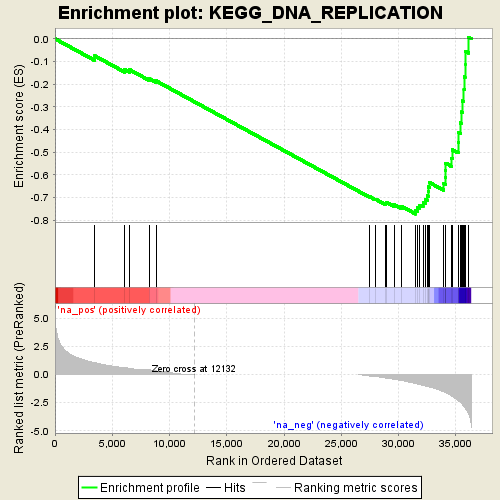


|  | PROBE | RANK IN GENE LIST | RANK METRIC SCORE | RUNNING ES | CORE ENRICHMENT |
| --- | --- | --- | --- | --- | --- |
| 1 | [PCNA](https://www.affymetrix.com/LinkServlet?probeset=PCNA) | 3477 | 1.044 | -0.0754 | No |
| 2 | [RNASEH1](https://www.affymetrix.com/LinkServlet?probeset=RNASEH1) | 6075 | 0.615 | -0.1349 | No |
| 3 | [POLE3](https://www.affymetrix.com/LinkServlet?probeset=POLE3) | 6524 | 0.542 | -0.1367 | No |
| 4 | [POLD4](https://www.affymetrix.com/LinkServlet?probeset=POLD4) | 8208 | 0.412 | -0.1750 | No |
| 5 | [POLE4](https://www.affymetrix.com/LinkServlet?probeset=POLE4) | 8816 | 0.319 | -0.1855 | No |
| 6 | [RFC4](https://www.affymetrix.com/LinkServlet?probeset=RFC4) | 27430 | -0.134 | -0.6956 | No |
| 7 | [POLD2](https://www.affymetrix.com/LinkServlet?probeset=POLD2) | 27971 | -0.167 | -0.7072 | No |
| 8 | [LIG1](https://www.affymetrix.com/LinkServlet?probeset=LIG1) | 28863 | -0.308 | -0.7258 | No |
| 9 | [RNASEH2C](https://www.affymetrix.com/LinkServlet?probeset=RNASEH2C) | 28959 | -0.324 | -0.7221 | No |
| 10 | [RPA3](https://www.affymetrix.com/LinkServlet?probeset=RPA3) | 29639 | -0.429 | -0.7324 | No |
| 11 | [FEN1](https://www.affymetrix.com/LinkServlet?probeset=FEN1) | 30243 | -0.527 | -0.7387 | No |
| 12 | [SSBP1](https://www.affymetrix.com/LinkServlet?probeset=SSBP1) | 31495 | -0.797 | -0.7576 | Yes |
| 13 | [RFC5](https://www.affymetrix.com/LinkServlet?probeset=RFC5) | 31626 | -0.833 | -0.7449 | Yes |
| 14 | [RPA2](https://www.affymetrix.com/LinkServlet?probeset=RPA2) | 31846 | -0.887 | -0.7337 | Yes |
| 15 | [MCM7](https://www.affymetrix.com/LinkServlet?probeset=MCM7) | 32135 | -0.959 | -0.7229 | Yes |
| 16 | [RNASEH2B](https://www.affymetrix.com/LinkServlet?probeset=RNASEH2B) | 32326 | -1.006 | -0.7085 | Yes |
| 17 | [RNASEH2A](https://www.affymetrix.com/LinkServlet?probeset=RNASEH2A) | 32508 | -1.055 | -0.6929 | Yes |
| 18 | [RPA1](https://www.affymetrix.com/LinkServlet?probeset=RPA1) | 32602 | -1.081 | -0.6743 | Yes |
| 19 | [MCM2](https://www.affymetrix.com/LinkServlet?probeset=MCM2) | 32623 | -1.087 | -0.6537 | Yes |
| 20 | [MCM5](https://www.affymetrix.com/LinkServlet?probeset=MCM5) | 32670 | -1.099 | -0.6335 | Yes |
| 21 | [POLD1](https://www.affymetrix.com/LinkServlet?probeset=POLD1) | 33953 | -1.545 | -0.6387 | Yes |
| 22 | [RPA4](https://www.affymetrix.com/LinkServlet?probeset=RPA4) | 34069 | -1.594 | -0.6107 | Yes |
| 23 | [POLD3](https://www.affymetrix.com/LinkServlet?probeset=POLD3) | 34110 | -1.614 | -0.5803 | Yes |
| 24 | [MCM4](https://www.affymetrix.com/LinkServlet?probeset=MCM4) | 34123 | -1.620 | -0.5490 | Yes |
| 25 | [RFC1](https://www.affymetrix.com/LinkServlet?probeset=RFC1) | 34642 | -1.906 | -0.5261 | Yes |
| 26 | [POLE](https://www.affymetrix.com/LinkServlet?probeset=POLE) | 34693 | -1.936 | -0.4897 | Yes |
| 27 | [DNA2](https://www.affymetrix.com/LinkServlet?probeset=DNA2) | 35198 | -2.298 | -0.4588 | Yes |
| 28 | [RFC2](https://www.affymetrix.com/LinkServlet?probeset=RFC2) | 35228 | -2.329 | -0.4141 | Yes |
| 29 | [MCM6](https://www.affymetrix.com/LinkServlet?probeset=MCM6) | 35398 | -2.467 | -0.3707 | Yes |
| 30 | [POLA2](https://www.affymetrix.com/LinkServlet?probeset=POLA2) | 35458 | -2.537 | -0.3228 | Yes |
| 31 | [RFC3](https://www.affymetrix.com/LinkServlet?probeset=RFC3) | 35585 | -2.669 | -0.2742 | Yes |
| 32 | [POLA1](https://www.affymetrix.com/LinkServlet?probeset=POLA1) | 35709 | -2.814 | -0.2226 | Yes |
| 33 | [PRIM1](https://www.affymetrix.com/LinkServlet?probeset=PRIM1) | 35744 | -2.864 | -0.1677 | Yes |
| 34 | [POLE2](https://www.affymetrix.com/LinkServlet?probeset=POLE2) | 35803 | -2.948 | -0.1118 | Yes |
| 35 | [MCM3](https://www.affymetrix.com/LinkServlet?probeset=MCM3) | 35809 | -2.959 | -0.0542 | Yes |
| 36 | [PRIM2](https://www.affymetrix.com/LinkServlet?probeset=PRIM2) | 36121 | -3.523 | 0.0060 | Yes |

| 2. KEGG_PHOSPHATIDYLINOSITOL_SIGNALING_SYSTEM | |  |  |
| --- | --- | --- | --- |
| Dataset | | GSEA_Human_AQ1 2 µMvsDMSO | |
| Phenotype | | NoPhenotypeAvailable | |
| Upregulated in class | | na_neg | |
| GeneSet | | KEGG_PHOSPHATIDYLINOSITOL_SIGNALING_SYSTEM | |
| Enrichment Score (ES) | | -0.63760126 | |
| Normalized Enrichment Score (NES) | | -1.6943592 | |
| Nominal p-value | | 0.0017271157 | |
| FDR q-value | | 0.008913939 | |
| FWER p-Value | | 0.076 | |


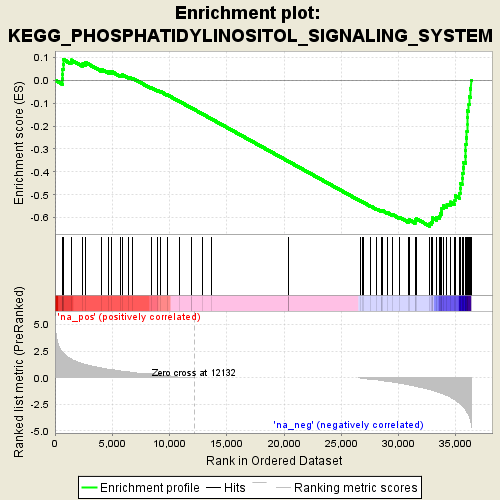


|  | PROBE | RANK IN GENE LIST | RANK METRIC SCORE | RUNNING ES | CORE ENRICHMENT |
| --- | --- | --- | --- | --- | --- |
| 1 | [PLCD4](https://www.affymetrix.com/LinkServlet?probeset=PLCD4) | 627 | 2.447 | 0.0054 | No |
| 2 | [PRKCG](https://www.affymetrix.com/LinkServlet?probeset=PRKCG) | 662 | 2.400 | 0.0266 | No |
| 3 | [INPP1](https://www.affymetrix.com/LinkServlet?probeset=INPP1) | 672 | 2.384 | 0.0485 | No |
| 4 | [CALML6](https://www.affymetrix.com/LinkServlet?probeset=CALML6) | 712 | 2.349 | 0.0691 | No |
| 5 | [CALML3](https://www.affymetrix.com/LinkServlet?probeset=CALML3) | 741 | 2.323 | 0.0899 | No |
| 6 | [PIP4K2C](https://www.affymetrix.com/LinkServlet?probeset=PIP4K2C) | 1395 | 1.770 | 0.0882 | No |
| 7 | [PLCD3](https://www.affymetrix.com/LinkServlet?probeset=PLCD3) | 2435 | 1.322 | 0.0718 | No |
| 8 | [INPP5E](https://www.affymetrix.com/LinkServlet?probeset=INPP5E) | 2684 | 1.243 | 0.0765 | No |
| 9 | [INPP5J](https://www.affymetrix.com/LinkServlet?probeset=INPP5J) | 4042 | 0.923 | 0.0476 | No |
| 10 | [CDIPT](https://www.affymetrix.com/LinkServlet?probeset=CDIPT) | 4694 | 0.800 | 0.0371 | No |
| 11 | [PLCD1](https://www.affymetrix.com/LinkServlet?probeset=PLCD1) | 4924 | 0.760 | 0.0378 | No |
| 12 | [CALM1](https://www.affymetrix.com/LinkServlet?probeset=CALM1) | 5702 | 0.642 | 0.0223 | No |
| 13 | [PIK3R3](https://www.affymetrix.com/LinkServlet?probeset=PIK3R3) | 5878 | 0.623 | 0.0232 | No |
| 14 | [PI4KB](https://www.affymetrix.com/LinkServlet?probeset=PI4KB) | 6442 | 0.555 | 0.0129 | No |
| 15 | [INPPL1](https://www.affymetrix.com/LinkServlet?probeset=INPPL1) | 6753 | 0.505 | 0.0090 | No |
| 16 | [INPP5K](https://www.affymetrix.com/LinkServlet?probeset=INPP5K) | 8378 | 0.388 | -0.0322 | No |
| 17 | [CALM3](https://www.affymetrix.com/LinkServlet?probeset=CALM3) | 8959 | 0.299 | -0.0454 | No |
| 18 | [PLCG1](https://www.affymetrix.com/LinkServlet?probeset=PLCG1) | 9185 | 0.277 | -0.0491 | No |
| 19 | [CALM2](https://www.affymetrix.com/LinkServlet?probeset=CALM2) | 9805 | 0.207 | -0.0642 | No |
| 20 | [DGKQ](https://www.affymetrix.com/LinkServlet?probeset=DGKQ) | 10821 | 0.060 | -0.0917 | No |
| 21 | [IMPA1](https://www.affymetrix.com/LinkServlet?probeset=IMPA1) | 11905 | 0.028 | -0.1213 | No |
| 22 | [DGKE](https://www.affymetrix.com/LinkServlet?probeset=DGKE) | 11913 | 0.027 | -0.1212 | No |
| 23 | [CALML5](https://www.affymetrix.com/LinkServlet?probeset=CALML5) | 12905 | 0.000 | -0.1485 | No |
| 24 | [DGKB](https://www.affymetrix.com/LinkServlet?probeset=DGKB) | 13681 | 0.000 | -0.1699 | No |
| 25 | [PIK3C2G](https://www.affymetrix.com/LinkServlet?probeset=PIK3C2G) | 20362 | 0.000 | -0.3541 | No |
| 26 | [PLCB4](https://www.affymetrix.com/LinkServlet?probeset=PLCB4) | 20387 | 0.000 | -0.3548 | No |
| 27 | [PLCZ1](https://www.affymetrix.com/LinkServlet?probeset=PLCZ1) | 20395 | 0.000 | -0.3550 | No |
| 28 | [DGKA](https://www.affymetrix.com/LinkServlet?probeset=DGKA) | 26703 | -0.029 | -0.5286 | No |
| 29 | [PLCE1](https://www.affymetrix.com/LinkServlet?probeset=PLCE1) | 26817 | -0.045 | -0.5313 | No |
| 30 | [PTEN](https://www.affymetrix.com/LinkServlet?probeset=PTEN) | 26947 | -0.062 | -0.5343 | No |
| 31 | [PIK3R2](https://www.affymetrix.com/LinkServlet?probeset=PIK3R2) | 27553 | -0.143 | -0.5497 | No |
| 32 | [PLCB3](https://www.affymetrix.com/LinkServlet?probeset=PLCB3) | 28070 | -0.181 | -0.5622 | No |
| 33 | [PIP5K1A](https://www.affymetrix.com/LinkServlet?probeset=PIP5K1A) | 28534 | -0.261 | -0.5726 | No |
| 34 | [DGKZ](https://www.affymetrix.com/LinkServlet?probeset=DGKZ) | 28549 | -0.263 | -0.5705 | No |
| 35 | [IPPK](https://www.affymetrix.com/LinkServlet?probeset=IPPK) | 28550 | -0.264 | -0.5681 | No |
| 36 | [PLCB2](https://www.affymetrix.com/LinkServlet?probeset=PLCB2) | 29015 | -0.332 | -0.5778 | No |
| 37 | [SYNJ1](https://www.affymetrix.com/LinkServlet?probeset=SYNJ1) | 29441 | -0.398 | -0.5858 | No |
| 38 | [PIK3CG](https://www.affymetrix.com/LinkServlet?probeset=PIK3CG) | 30075 | -0.498 | -0.5987 | No |
| 39 | [PIP4K2B](https://www.affymetrix.com/LinkServlet?probeset=PIP4K2B) | 30848 | -0.656 | -0.6139 | No |
| 40 | [PIKFYVE](https://www.affymetrix.com/LinkServlet?probeset=PIKFYVE) | 30908 | -0.669 | -0.6093 | No |
| 41 | [CDS2](https://www.affymetrix.com/LinkServlet?probeset=CDS2) | 31436 | -0.786 | -0.6166 | No |
| 42 | [PIK3CA](https://www.affymetrix.com/LinkServlet?probeset=PIK3CA) | 31455 | -0.789 | -0.6098 | No |
| 43 | [PIK3CD](https://www.affymetrix.com/LinkServlet?probeset=PIK3CD) | 31545 | -0.812 | -0.6047 | No |
| 44 | [PIK3R1](https://www.affymetrix.com/LinkServlet?probeset=PIK3R1) | 32739 | -1.120 | -0.6272 | Yes |
| 45 | [PIK3C2A](https://www.affymetrix.com/LinkServlet?probeset=PIK3C2A) | 32850 | -1.152 | -0.6196 | Yes |
| 46 | [INPP5B](https://www.affymetrix.com/LinkServlet?probeset=INPP5B) | 32943 | -1.176 | -0.6113 | Yes |
| 47 | [OCRL](https://www.affymetrix.com/LinkServlet?probeset=OCRL) | 32979 | -1.187 | -0.6012 | Yes |
| 48 | [SYNJ2](https://www.affymetrix.com/LinkServlet?probeset=SYNJ2) | 33329 | -1.300 | -0.5988 | Yes |
| 49 | [PIK3C3](https://www.affymetrix.com/LinkServlet?probeset=PIK3C3) | 33577 | -1.393 | -0.5927 | Yes |
| 50 | [ITPKA](https://www.affymetrix.com/LinkServlet?probeset=ITPKA) | 33618 | -1.407 | -0.5808 | Yes |
| 51 | [PI4KA](https://www.affymetrix.com/LinkServlet?probeset=PI4KA) | 33773 | -1.465 | -0.5715 | Yes |
| 52 | [CDS1](https://www.affymetrix.com/LinkServlet?probeset=CDS1) | 33780 | -1.466 | -0.5581 | Yes |
| 53 | [PIK3C2B](https://www.affymetrix.com/LinkServlet?probeset=PIK3C2B) | 33889 | -1.515 | -0.5471 | Yes |
| 54 | [DGKH](https://www.affymetrix.com/LinkServlet?probeset=DGKH) | 34225 | -1.672 | -0.5408 | Yes |
| 55 | [PIK3R5](https://www.affymetrix.com/LinkServlet?probeset=PIK3R5) | 34501 | -1.819 | -0.5316 | Yes |
| 56 | [ITPKB](https://www.affymetrix.com/LinkServlet?probeset=ITPKB) | 34920 | -2.085 | -0.5238 | Yes |
| 57 | [DGKI](https://www.affymetrix.com/LinkServlet?probeset=DGKI) | 34930 | -2.092 | -0.5047 | Yes |
| 58 | [INPP5D](https://www.affymetrix.com/LinkServlet?probeset=INPP5D) | 35308 | -2.390 | -0.4930 | Yes |
| 59 | [ITPR3](https://www.affymetrix.com/LinkServlet?probeset=ITPR3) | 35369 | -2.441 | -0.4720 | Yes |
| 60 | [PIP5K1C](https://www.affymetrix.com/LinkServlet?probeset=PIP5K1C) | 35400 | -2.470 | -0.4500 | Yes |
| 61 | [PRKCB](https://www.affymetrix.com/LinkServlet?probeset=PRKCB) | 35542 | -2.624 | -0.4296 | Yes |
| 62 | [DGKD](https://www.affymetrix.com/LinkServlet?probeset=DGKD) | 35581 | -2.665 | -0.4060 | Yes |
| 63 | [PLCG2](https://www.affymetrix.com/LinkServlet?probeset=PLCG2) | 35631 | -2.732 | -0.3820 | Yes |
| 64 | [INPP4A](https://www.affymetrix.com/LinkServlet?probeset=INPP4A) | 35687 | -2.787 | -0.3577 | Yes |
| 65 | [PIP4K2A](https://www.affymetrix.com/LinkServlet?probeset=PIP4K2A) | 35837 | -2.993 | -0.3341 | Yes |
| 66 | [ITPR1](https://www.affymetrix.com/LinkServlet?probeset=ITPR1) | 35852 | -3.013 | -0.3066 | Yes |
| 67 | [ITPR2](https://www.affymetrix.com/LinkServlet?probeset=ITPR2) | 35875 | -3.055 | -0.2790 | Yes |
| 68 | [IMPA2](https://www.affymetrix.com/LinkServlet?probeset=IMPA2) | 35896 | -3.086 | -0.2510 | Yes |
| 69 | [PIK3CB](https://www.affymetrix.com/LinkServlet?probeset=PIK3CB) | 35969 | -3.215 | -0.2232 | Yes |
| 70 | [DGKG](https://www.affymetrix.com/LinkServlet?probeset=DGKG) | 35982 | -3.242 | -0.1935 | Yes |
| 71 | [PLCB1](https://www.affymetrix.com/LinkServlet?probeset=PLCB1) | 35986 | -3.246 | -0.1635 | Yes |
| 72 | [ITPK1](https://www.affymetrix.com/LinkServlet?probeset=ITPK1) | 35997 | -3.265 | -0.1336 | Yes |
| 73 | [PRKCA](https://www.affymetrix.com/LinkServlet?probeset=PRKCA) | 36143 | -3.575 | -0.1045 | Yes |
| 74 | [PIP5K1B](https://www.affymetrix.com/LinkServlet?probeset=PIP5K1B) | 36161 | -3.626 | -0.0714 | Yes |
| 75 | [INPP5A](https://www.affymetrix.com/LinkServlet?probeset=INPP5A) | 36283 | -4.000 | -0.0377 | Yes |
| 76 | [INPP4B](https://www.affymetrix.com/LinkServlet?probeset=INPP4B) | 36322 | -4.238 | 0.0005 | Yes |

3. KEGG_HOMOLOGOUS_RECOMBINATION

| Dataset | GSEA_Human_AQ1 2 µMvsDMSO |
| --- | --- |
| Phenotype | NoPhenotypeAvailable |
| Upregulated in class | na_neg |
| GeneSet | KEGG_HOMOLOGOUS_RECOMBINATION |
| Enrichment Score (ES) | -0.7878233 |
| Normalized Enrichment Score (NES) | -1.7285544 |
| Nominal p-value | 0.0018552876 |
| FDR q-value | 0.009233484 |
| FWER p-Value | 0.043 |


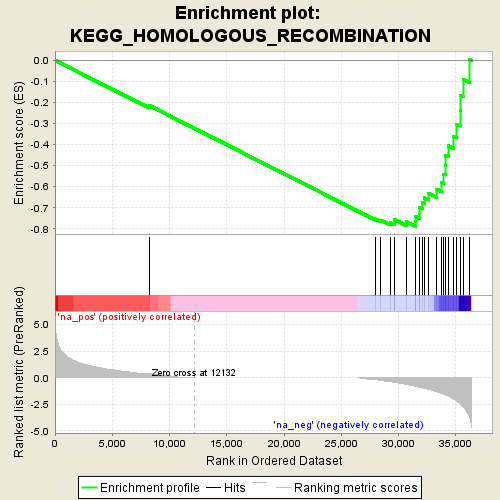


|  | PROBE | RANK IN GENE LIST | RANK METRIC SCORE | RUNNING ES | CORE ENRICHMENT |
| --- | --- | --- | --- | --- | --- |
| 1 | [POLD4](https://www.affymetrix.com/LinkServlet?probeset=POLD4) | 8208 | 0.412 | -0.2139 | No |
| 2 | [POLD2](https://www.affymetrix.com/LinkServlet?probeset=POLD2) | 27971 | -0.167 | -0.7531 | No |
| 3 | [TOP3B](https://www.affymetrix.com/LinkServlet?probeset=TOP3B) | 28422 | -0.242 | -0.7584 | No |
| 4 | [RAD52](https://www.affymetrix.com/LinkServlet?probeset=RAD52) | 29266 | -0.371 | -0.7707 | Yes |
| 5 | [XRCC3](https://www.affymetrix.com/LinkServlet?probeset=XRCC3) | 29623 | -0.426 | -0.7679 | Yes |
| 6 | [RPA3](https://www.affymetrix.com/LinkServlet?probeset=RPA3) | 29639 | -0.429 | -0.7556 | Yes |
| 7 | [RAD51C](https://www.affymetrix.com/LinkServlet?probeset=RAD51C) | 30676 | -0.620 | -0.7659 | Yes |
| 8 | [EME1](https://www.affymetrix.com/LinkServlet?probeset=EME1) | 31474 | -0.793 | -0.7644 | Yes |
| 9 | [SSBP1](https://www.affymetrix.com/LinkServlet?probeset=SSBP1) | 31495 | -0.797 | -0.7415 | Yes |
| 10 | [MUS81](https://www.affymetrix.com/LinkServlet?probeset=MUS81) | 31843 | -0.886 | -0.7249 | Yes |
| 11 | [RPA2](https://www.affymetrix.com/LinkServlet?probeset=RPA2) | 31846 | -0.887 | -0.6988 | Yes |
| 12 | [TOP3A](https://www.affymetrix.com/LinkServlet?probeset=TOP3A) | 32072 | -0.947 | -0.6770 | Yes |
| 13 | [NBN](https://www.affymetrix.com/LinkServlet?probeset=NBN) | 32241 | -0.986 | -0.6526 | Yes |
| 14 | [RPA1](https://www.affymetrix.com/LinkServlet?probeset=RPA1) | 32602 | -1.081 | -0.6306 | Yes |
| 15 | [XRCC2](https://www.affymetrix.com/LinkServlet?probeset=XRCC2) | 33334 | -1.302 | -0.6123 | Yes |
| 16 | [RAD54L](https://www.affymetrix.com/LinkServlet?probeset=RAD54L) | 33746 | -1.453 | -0.5808 | Yes |
| 17 | [POLD1](https://www.affymetrix.com/LinkServlet?probeset=POLD1) | 33953 | -1.545 | -0.5408 | Yes |
| 18 | [RPA4](https://www.affymetrix.com/LinkServlet?probeset=RPA4) | 34069 | -1.594 | -0.4970 | Yes |
| 19 | [POLD3](https://www.affymetrix.com/LinkServlet?probeset=POLD3) | 34110 | -1.614 | -0.4505 | Yes |
| 20 | [RAD51D](https://www.affymetrix.com/LinkServlet?probeset=RAD51D) | 34333 | -1.719 | -0.4059 | Yes |
| 21 | [RAD50](https://www.affymetrix.com/LinkServlet?probeset=RAD50) | 34806 | -2.009 | -0.3596 | Yes |
| 22 | [BRCA2](https://www.affymetrix.com/LinkServlet?probeset=BRCA2) | 35074 | -2.193 | -0.3023 | Yes |
| 23 | [RAD51](https://www.affymetrix.com/LinkServlet?probeset=RAD51) | 35384 | -2.457 | -0.2383 | Yes |
| 24 | [RAD54B](https://www.affymetrix.com/LinkServlet?probeset=RAD54B) | 35415 | -2.490 | -0.1657 | Yes |
| 25 | [BLM](https://www.affymetrix.com/LinkServlet?probeset=BLM) | 35644 | -2.742 | -0.0911 | Yes |
| 26 | [RAD51B](https://www.affymetrix.com/LinkServlet?probeset=RAD51B) | 36205 | -3.735 | 0.0037 | Yes |

4. KEGG_CELL_CYCLE

|  |  |
| --- | --- |
| Dataset | GSEA_Human_AQ1 2 µMvsDMSO |
| Phenotype | NoPhenotypeAvailable |
| Upregulated in class | na_neg |
| GeneSet | KEGG_CELL_CYCLE |
| Enrichment Score (ES) | -0.5822364 |
| Normalized Enrichment Score (NES) | -1.6288642 |
| Nominal p-value | 0.0 |
| FDR q-value | 0.014391109 |
| FWER p-Value | 0.205 |


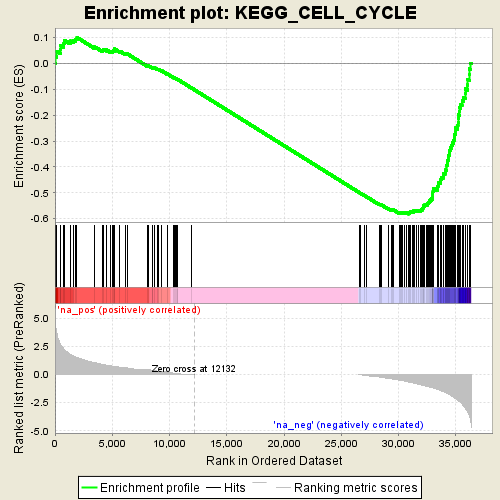


|  | PROBE | RANK IN GENE LIST | RANK METRIC SCORE | RUNNING ES | CORE ENRICHMENT |
| --- | --- | --- | --- | --- | --- |
| 1 | [GADD45A](https://www.affymetrix.com/LinkServlet?probeset=GADD45A) | 46 | 4.423 | 0.0264 | No |
| 2 | [CDKN1A](https://www.affymetrix.com/LinkServlet?probeset=CDKN1A) | 147 | 3.680 | 0.0467 | No |
| 3 | [GADD45G](https://www.affymetrix.com/LinkServlet?probeset=GADD45G) | 477 | 2.683 | 0.0544 | No |
| 4 | [SMAD3](https://www.affymetrix.com/LinkServlet?probeset=SMAD3) | 500 | 2.638 | 0.0703 | No |
| 5 | [MDM2](https://www.affymetrix.com/LinkServlet?probeset=MDM2) | 770 | 2.278 | 0.0771 | No |
| 6 | [CDKN1C](https://www.affymetrix.com/LinkServlet?probeset=CDKN1C) | 789 | 2.247 | 0.0907 | No |
| 7 | [CDKN1B](https://www.affymetrix.com/LinkServlet?probeset=CDKN1B) | 1330 | 1.806 | 0.0870 | No |
| 8 | [GADD45B](https://www.affymetrix.com/LinkServlet?probeset=GADD45B) | 1608 | 1.649 | 0.0897 | No |
| 9 | [TP53](https://www.affymetrix.com/LinkServlet?probeset=TP53) | 1797 | 1.556 | 0.0943 | No |
| 10 | [CDKN2B](https://www.affymetrix.com/LinkServlet?probeset=CDKN2B) | 1912 | 1.516 | 0.1006 | No |
| 11 | [PCNA](https://www.affymetrix.com/LinkServlet?probeset=PCNA) | 3477 | 1.044 | 0.0639 | No |
| 12 | [TGFB3](https://www.affymetrix.com/LinkServlet?probeset=TGFB3) | 4125 | 0.905 | 0.0517 | No |
| 13 | [WEE1](https://www.affymetrix.com/LinkServlet?probeset=WEE1) | 4190 | 0.892 | 0.0555 | No |
| 14 | [SFN](https://www.affymetrix.com/LinkServlet?probeset=SFN) | 4460 | 0.839 | 0.0534 | No |
| 15 | [PTTG2](https://www.affymetrix.com/LinkServlet?probeset=PTTG2) | 4879 | 0.768 | 0.0466 | No |
| 16 | [CDC23](https://www.affymetrix.com/LinkServlet?probeset=CDC23) | 5020 | 0.741 | 0.0474 | No |
| 17 | [CCNA1](https://www.affymetrix.com/LinkServlet?probeset=CCNA1) | 5071 | 0.738 | 0.0506 | No |
| 18 | [YWHAZ](https://www.affymetrix.com/LinkServlet?probeset=YWHAZ) | 5197 | 0.725 | 0.0517 | No |
| 19 | [PTTG1](https://www.affymetrix.com/LinkServlet?probeset=PTTG1) | 5198 | 0.724 | 0.0563 | No |
| 20 | [CDC16](https://www.affymetrix.com/LinkServlet?probeset=CDC16) | 5653 | 0.650 | 0.0478 | No |
| 21 | [SMAD4](https://www.affymetrix.com/LinkServlet?probeset=SMAD4) | 6132 | 0.604 | 0.0384 | No |
| 22 | [CCND1](https://www.affymetrix.com/LinkServlet?probeset=CCND1) | 6286 | 0.582 | 0.0378 | No |
| 23 | [ANAPC5](https://www.affymetrix.com/LinkServlet?probeset=ANAPC5) | 8091 | 0.426 | -0.0094 | No |
| 24 | [CCND2](https://www.affymetrix.com/LinkServlet?probeset=CCND2) | 8161 | 0.418 | -0.0087 | No |
| 25 | [RAD21](https://www.affymetrix.com/LinkServlet?probeset=RAD21) | 8519 | 0.366 | -0.0162 | No |
| 26 | [ANAPC4](https://www.affymetrix.com/LinkServlet?probeset=ANAPC4) | 8521 | 0.366 | -0.0140 | No |
| 27 | [YWHAH](https://www.affymetrix.com/LinkServlet?probeset=YWHAH) | 8708 | 0.332 | -0.0170 | No |
| 28 | [ANAPC2](https://www.affymetrix.com/LinkServlet?probeset=ANAPC2) | 8936 | 0.303 | -0.0214 | No |
| 29 | [SKP1](https://www.affymetrix.com/LinkServlet?probeset=SKP1) | 9022 | 0.291 | -0.0219 | No |
| 30 | [ZBTB17](https://www.affymetrix.com/LinkServlet?probeset=ZBTB17) | 9314 | 0.261 | -0.0283 | No |
| 31 | [RBX1](https://www.affymetrix.com/LinkServlet?probeset=RBX1) | 9797 | 0.208 | -0.0403 | No |
| 32 | [ANAPC7](https://www.affymetrix.com/LinkServlet?probeset=ANAPC7) | 10373 | 0.118 | -0.0555 | No |
| 33 | [ANAPC11](https://www.affymetrix.com/LinkServlet?probeset=ANAPC11) | 10389 | 0.115 | -0.0552 | No |
| 34 | [ANAPC13](https://www.affymetrix.com/LinkServlet?probeset=ANAPC13) | 10492 | 0.100 | -0.0573 | No |
| 35 | [HDAC1](https://www.affymetrix.com/LinkServlet?probeset=HDAC1) | 10626 | 0.086 | -0.0605 | No |
| 36 | [WEE2](https://www.affymetrix.com/LinkServlet?probeset=WEE2) | 10651 | 0.083 | -0.0606 | No |
| 37 | [CDC26](https://www.affymetrix.com/LinkServlet?probeset=CDC26) | 10687 | 0.077 | -0.0611 | No |
| 38 | [YWHAG](https://www.affymetrix.com/LinkServlet?probeset=YWHAG) | 11875 | 0.032 | -0.0937 | No |
| 39 | [TGFB2](https://www.affymetrix.com/LinkServlet?probeset=TGFB2) | 26545 | -0.004 | -0.4987 | No |
| 40 | [CDKN2A](https://www.affymetrix.com/LinkServlet?probeset=CDKN2A) | 26635 | -0.019 | -0.5010 | No |
| 41 | [CDKN2C](https://www.affymetrix.com/LinkServlet?probeset=CDKN2C) | 26994 | -0.071 | -0.5105 | No |
| 42 | [FZR1](https://www.affymetrix.com/LinkServlet?probeset=FZR1) | 27161 | -0.099 | -0.5144 | No |
| 43 | [TGFB1](https://www.affymetrix.com/LinkServlet?probeset=TGFB1) | 28345 | -0.228 | -0.5457 | No |
| 44 | [EP300](https://www.affymetrix.com/LinkServlet?probeset=EP300) | 28408 | -0.240 | -0.5459 | No |
| 45 | [DBF4](https://www.affymetrix.com/LinkServlet?probeset=DBF4) | 28502 | -0.255 | -0.5469 | No |
| 46 | [ORC6](https://www.affymetrix.com/LinkServlet?probeset=ORC6) | 29137 | -0.351 | -0.5622 | No |
| 47 | [RBL2](https://www.affymetrix.com/LinkServlet?probeset=RBL2) | 29368 | -0.387 | -0.5661 | No |
| 48 | [CDK4](https://www.affymetrix.com/LinkServlet?probeset=CDK4) | 29439 | -0.398 | -0.5655 | No |
| 49 | [CDC25B](https://www.affymetrix.com/LinkServlet?probeset=CDC25B) | 29512 | -0.410 | -0.5650 | No |
| 50 | [CDK7](https://www.affymetrix.com/LinkServlet?probeset=CDK7) | 30113 | -0.505 | -0.5784 | Yes |
| 51 | [E2F4](https://www.affymetrix.com/LinkServlet?probeset=E2F4) | 30183 | -0.517 | -0.5771 | Yes |
| 52 | [CDKN2D](https://www.affymetrix.com/LinkServlet?probeset=CDKN2D) | 30246 | -0.527 | -0.5755 | Yes |
| 53 | [SMAD2](https://www.affymetrix.com/LinkServlet?probeset=SMAD2) | 30364 | -0.554 | -0.5752 | Yes |
| 54 | [YWHAB](https://www.affymetrix.com/LinkServlet?probeset=YWHAB) | 30546 | -0.591 | -0.5765 | Yes |
| 55 | [CDK1](https://www.affymetrix.com/LinkServlet?probeset=CDK1) | 30724 | -0.629 | -0.5775 | Yes |
| 56 | [MAD2L2](https://www.affymetrix.com/LinkServlet?probeset=MAD2L2) | 30897 | -0.666 | -0.5781 | Yes |
| 57 | [ORC4](https://www.affymetrix.com/LinkServlet?probeset=ORC4) | 30923 | -0.674 | -0.5745 | Yes |
| 58 | [E2F5](https://www.affymetrix.com/LinkServlet?probeset=E2F5) | 31041 | -0.701 | -0.5734 | Yes |
| 59 | [HDAC2](https://www.affymetrix.com/LinkServlet?probeset=HDAC2) | 31184 | -0.734 | -0.5727 | Yes |
| 60 | [CDK2](https://www.affymetrix.com/LinkServlet?probeset=CDK2) | 31270 | -0.754 | -0.5703 | Yes |
| 61 | [YWHAQ](https://www.affymetrix.com/LinkServlet?probeset=YWHAQ) | 31351 | -0.772 | -0.5677 | Yes |
| 62 | [CDC25C](https://www.affymetrix.com/LinkServlet?probeset=CDC25C) | 31531 | -0.808 | -0.5676 | Yes |
| 63 | [ABL1](https://www.affymetrix.com/LinkServlet?probeset=ABL1) | 31727 | -0.858 | -0.5676 | Yes |
| 64 | [TFDP2](https://www.affymetrix.com/LinkServlet?probeset=TFDP2) | 31902 | -0.903 | -0.5668 | Yes |
| 65 | [ATM](https://www.affymetrix.com/LinkServlet?probeset=ATM) | 32022 | -0.933 | -0.5642 | Yes |
| 66 | [YWHAE](https://www.affymetrix.com/LinkServlet?probeset=YWHAE) | 32117 | -0.955 | -0.5608 | Yes |
| 67 | [MCM7](https://www.affymetrix.com/LinkServlet?probeset=MCM7) | 32135 | -0.959 | -0.5553 | Yes |
| 68 | [BUB3](https://www.affymetrix.com/LinkServlet?probeset=BUB3) | 32150 | -0.962 | -0.5497 | Yes |
| 69 | [MYC](https://www.affymetrix.com/LinkServlet?probeset=MYC) | 32289 | -0.997 | -0.5473 | Yes |
| 70 | [CCNH](https://www.affymetrix.com/LinkServlet?probeset=CCNH) | 32403 | -1.029 | -0.5439 | Yes |
| 71 | [CDC7](https://www.affymetrix.com/LinkServlet?probeset=CDC7) | 32535 | -1.064 | -0.5409 | Yes |
| 72 | [MCM2](https://www.affymetrix.com/LinkServlet?probeset=MCM2) | 32623 | -1.087 | -0.5365 | Yes |
| 73 | [MCM5](https://www.affymetrix.com/LinkServlet?probeset=MCM5) | 32670 | -1.099 | -0.5309 | Yes |
| 74 | [SMC3](https://www.affymetrix.com/LinkServlet?probeset=SMC3) | 32784 | -1.131 | -0.5269 | Yes |
| 75 | [CCNB2](https://www.affymetrix.com/LinkServlet?probeset=CCNB2) | 32880 | -1.158 | -0.5223 | Yes |
| 76 | [TFDP1](https://www.affymetrix.com/LinkServlet?probeset=TFDP1) | 32966 | -1.182 | -0.5173 | Yes |
| 77 | [E2F3](https://www.affymetrix.com/LinkServlet?probeset=E2F3) | 32980 | -1.187 | -0.5102 | Yes |
| 78 | [ATR](https://www.affymetrix.com/LinkServlet?probeset=ATR) | 32984 | -1.189 | -0.5028 | Yes |
| 79 | [CDC27](https://www.affymetrix.com/LinkServlet?probeset=CDC27) | 33002 | -1.196 | -0.4958 | Yes |
| 80 | [CUL1](https://www.affymetrix.com/LinkServlet?probeset=CUL1) | 33021 | -1.202 | -0.4888 | Yes |
| 81 | [SMC1A](https://www.affymetrix.com/LinkServlet?probeset=SMC1A) | 33069 | -1.219 | -0.4825 | Yes |
| 82 | [CCNB1](https://www.affymetrix.com/LinkServlet?probeset=CCNB1) | 33374 | -1.316 | -0.4826 | Yes |
| 83 | [CHEK1](https://www.affymetrix.com/LinkServlet?probeset=CHEK1) | 33418 | -1.331 | -0.4755 | Yes |
| 84 | [CCNA2](https://www.affymetrix.com/LinkServlet?probeset=CCNA2) | 33447 | -1.341 | -0.4679 | Yes |
| 85 | [CDC20](https://www.affymetrix.com/LinkServlet?probeset=CDC20) | 33483 | -1.356 | -0.4604 | Yes |
| 86 | [PKMYT1](https://www.affymetrix.com/LinkServlet?probeset=PKMYT1) | 33651 | -1.417 | -0.4561 | Yes |
| 87 | [CDC14B](https://www.affymetrix.com/LinkServlet?probeset=CDC14B) | 33692 | -1.432 | -0.4482 | Yes |
| 88 | [PLK1](https://www.affymetrix.com/LinkServlet?probeset=PLK1) | 33742 | -1.451 | -0.4405 | Yes |
| 89 | [MAD2L1](https://www.affymetrix.com/LinkServlet?probeset=MAD2L1) | 33882 | -1.512 | -0.4349 | Yes |
| 90 | [CCNE1](https://www.affymetrix.com/LinkServlet?probeset=CCNE1) | 33941 | -1.536 | -0.4269 | Yes |
| 91 | [CCNE2](https://www.affymetrix.com/LinkServlet?probeset=CCNE2) | 34055 | -1.587 | -0.4201 | Yes |
| 92 | [MCM4](https://www.affymetrix.com/LinkServlet?probeset=MCM4) | 34123 | -1.620 | -0.4118 | Yes |
| 93 | [BUB1](https://www.affymetrix.com/LinkServlet?probeset=BUB1) | 34179 | -1.650 | -0.4030 | Yes |
| 94 | [ANAPC10](https://www.affymetrix.com/LinkServlet?probeset=ANAPC10) | 34180 | -1.650 | -0.3927 | Yes |
| 95 | [STAG2](https://www.affymetrix.com/LinkServlet?probeset=STAG2) | 34267 | -1.689 | -0.3845 | Yes |
| 96 | [TTK](https://www.affymetrix.com/LinkServlet?probeset=TTK) | 34285 | -1.696 | -0.3743 | Yes |
| 97 | [CREBBP](https://www.affymetrix.com/LinkServlet?probeset=CREBBP) | 34326 | -1.715 | -0.3647 | Yes |
| 98 | [CHEK2](https://www.affymetrix.com/LinkServlet?probeset=CHEK2) | 34335 | -1.719 | -0.3542 | Yes |
| 99 | [SKP2](https://www.affymetrix.com/LinkServlet?probeset=SKP2) | 34412 | -1.755 | -0.3453 | Yes |
| 100 | [ORC1](https://www.affymetrix.com/LinkServlet?probeset=ORC1) | 34436 | -1.772 | -0.3348 | Yes |
| 101 | [ORC2](https://www.affymetrix.com/LinkServlet?probeset=ORC2) | 34550 | -1.854 | -0.3264 | Yes |
| 102 | [ANAPC1](https://www.affymetrix.com/LinkServlet?probeset=ANAPC1) | 34601 | -1.881 | -0.3160 | Yes |
| 103 | [E2F2](https://www.affymetrix.com/LinkServlet?probeset=E2F2) | 34715 | -1.951 | -0.3069 | Yes |
| 104 | [PRKDC](https://www.affymetrix.com/LinkServlet?probeset=PRKDC) | 34773 | -1.985 | -0.2960 | Yes |
| 105 | [ORC3](https://www.affymetrix.com/LinkServlet?probeset=ORC3) | 34875 | -2.055 | -0.2860 | Yes |
| 106 | [RB1](https://www.affymetrix.com/LinkServlet?probeset=RB1) | 34902 | -2.075 | -0.2737 | Yes |
| 107 | [ESPL1](https://www.affymetrix.com/LinkServlet?probeset=ESPL1) | 34979 | -2.127 | -0.2625 | Yes |
| 108 | [E2F1](https://www.affymetrix.com/LinkServlet?probeset=E2F1) | 34987 | -2.134 | -0.2493 | Yes |
| 109 | [CCND3](https://www.affymetrix.com/LinkServlet?probeset=CCND3) | 35153 | -2.264 | -0.2397 | Yes |
| 110 | [CDC6](https://www.affymetrix.com/LinkServlet?probeset=CDC6) | 35190 | -2.291 | -0.2264 | Yes |
| 111 | [CDC14A](https://www.affymetrix.com/LinkServlet?probeset=CDC14A) | 35208 | -2.306 | -0.2124 | Yes |
| 112 | [CDC45](https://www.affymetrix.com/LinkServlet?probeset=CDC45) | 35246 | -2.348 | -0.1988 | Yes |
| 113 | [SMC1B](https://www.affymetrix.com/LinkServlet?probeset=SMC1B) | 35305 | -2.389 | -0.1854 | Yes |
| 114 | [ORC5](https://www.affymetrix.com/LinkServlet?probeset=ORC5) | 35345 | -2.422 | -0.1713 | Yes |
| 115 | [MCM6](https://www.affymetrix.com/LinkServlet?probeset=MCM6) | 35398 | -2.467 | -0.1573 | Yes |
| 116 | [CDC25A](https://www.affymetrix.com/LinkServlet?probeset=CDC25A) | 35541 | -2.623 | -0.1448 | Yes |
| 117 | [MAD1L1](https://www.affymetrix.com/LinkServlet?probeset=MAD1L1) | 35660 | -2.753 | -0.1309 | Yes |
| 118 | [MCM3](https://www.affymetrix.com/LinkServlet?probeset=MCM3) | 35809 | -2.959 | -0.1164 | Yes |
| 119 | [GSK3B](https://www.affymetrix.com/LinkServlet?probeset=GSK3B) | 35831 | -2.988 | -0.0983 | Yes |
| 120 | [CCNB3](https://www.affymetrix.com/LinkServlet?probeset=CCNB3) | 36034 | -3.326 | -0.0831 | Yes |
| 121 | [BUB1B](https://www.affymetrix.com/LinkServlet?probeset=BUB1B) | 36048 | -3.356 | -0.0625 | Yes |
| 122 | [CDK6](https://www.affymetrix.com/LinkServlet?probeset=CDK6) | 36162 | -3.629 | -0.0429 | Yes |
| 123 | [RBL1](https://www.affymetrix.com/LinkServlet?probeset=RBL1) | 36171 | -3.650 | -0.0203 | Yes |
| 124 | [STAG1](https://www.affymetrix.com/LinkServlet?probeset=STAG1) | 36275 | -3.975 | 0.0018 | Yes |

5. KEGG_COMPLEMENT_AND_COAGULATION_CASCADES

|  |  |
| --- | --- |
| Dataset | GSEA_Human_AQ1 2 µMvsDMSO |
| Phenotype | NoPhenotypeAvailable |
| Upregulated in class | na_pos |
| GeneSet | KEGG_COMPLEMENT_AND_COAGULATION_CASCADES |
| Enrichment Score (ES) | 0.68904805 |
| Normalized Enrichment Score (NES) | 1.8417476 |
| Nominal p-value | 0.0 |
| FDR q-value | 6.401953E-4 |
| FWER p-Value | 0.004 |


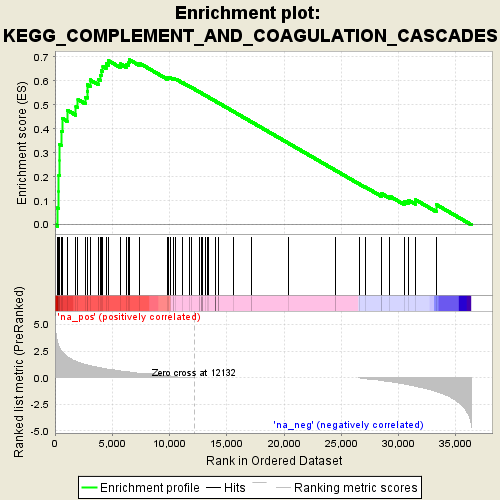


|  | PROBE | RANK IN GENE LIST | RANK METRIC SCORE | RUNNING ES | CORE ENRICHMENT |
| --- | --- | --- | --- | --- | --- |
| 1 | [C4B](https://www.affymetrix.com/LinkServlet?probeset=C4B) | 239 | 3.299 | 0.0699 | Yes |
| 2 | [C7](https://www.affymetrix.com/LinkServlet?probeset=C7) | 332 | 3.016 | 0.1373 | Yes |
| 3 | [SERPINC1](https://www.affymetrix.com/LinkServlet?probeset=SERPINC1) | 336 | 3.011 | 0.2070 | Yes |
| 4 | [SERPINF2](https://www.affymetrix.com/LinkServlet?probeset=SERPINF2) | 417 | 2.797 | 0.2696 | Yes |
| 5 | [F2R](https://www.affymetrix.com/LinkServlet?probeset=F2R) | 419 | 2.797 | 0.3344 | Yes |
| 6 | [PROC](https://www.affymetrix.com/LinkServlet?probeset=PROC) | 585 | 2.505 | 0.3880 | Yes |
| 7 | [C4BPB](https://www.affymetrix.com/LinkServlet?probeset=C4BPB) | 621 | 2.452 | 0.4439 | Yes |
| 8 | [C1R](https://www.affymetrix.com/LinkServlet?probeset=C1R) | 1047 | 1.999 | 0.4785 | Yes |
| 9 | [KLKB1](https://www.affymetrix.com/LinkServlet?probeset=KLKB1) | 1823 | 1.546 | 0.4929 | Yes |
| 10 | [C4A](https://www.affymetrix.com/LinkServlet?probeset=C4A) | 1999 | 1.474 | 0.5223 | Yes |
| 11 | [C1QA](https://www.affymetrix.com/LinkServlet?probeset=C1QA) | 2668 | 1.247 | 0.5328 | Yes |
| 12 | [A2M](https://www.affymetrix.com/LinkServlet?probeset=A2M) | 2815 | 1.205 | 0.5567 | Yes |
| 13 | [MASP2](https://www.affymetrix.com/LinkServlet?probeset=MASP2) | 2850 | 1.198 | 0.5835 | Yes |
| 14 | [C3](https://www.affymetrix.com/LinkServlet?probeset=C3) | 3061 | 1.145 | 0.6043 | Yes |
| 15 | [CD59](https://www.affymetrix.com/LinkServlet?probeset=CD59) | 3819 | 0.972 | 0.6059 | Yes |
| 16 | [C1S](https://www.affymetrix.com/LinkServlet?probeset=C1S) | 3974 | 0.935 | 0.6234 | Yes |
| 17 | [SERPING1](https://www.affymetrix.com/LinkServlet?probeset=SERPING1) | 4063 | 0.918 | 0.6422 | Yes |
| 18 | [C2](https://www.affymetrix.com/LinkServlet?probeset=C2) | 4181 | 0.894 | 0.6597 | Yes |
| 19 | [PLAUR](https://www.affymetrix.com/LinkServlet?probeset=PLAUR) | 4454 | 0.841 | 0.6717 | Yes |
| 20 | [CD46](https://www.affymetrix.com/LinkServlet?probeset=CD46) | 4687 | 0.801 | 0.6839 | Yes |
| 21 | [C6](https://www.affymetrix.com/LinkServlet?probeset=C6) | 5695 | 0.643 | 0.6710 | Yes |
| 22 | [SERPINE1](https://www.affymetrix.com/LinkServlet?probeset=SERPINE1) | 6243 | 0.588 | 0.6696 | Yes |
| 23 | [F8](https://www.affymetrix.com/LinkServlet?probeset=F8) | 6448 | 0.554 | 0.6768 | Yes |
| 24 | [CD55](https://www.affymetrix.com/LinkServlet?probeset=CD55) | 6467 | 0.551 | 0.6890 | Yes |
| 25 | [CFI](https://www.affymetrix.com/LinkServlet?probeset=CFI) | 7358 | 0.427 | 0.6744 | No |
| 26 | [CFH](https://www.affymetrix.com/LinkServlet?probeset=CFH) | 9826 | 0.204 | 0.6111 | No |
| 27 | [SERPIND1](https://www.affymetrix.com/LinkServlet?probeset=SERPIND1) | 9882 | 0.196 | 0.6141 | No |
| 28 | [F12](https://www.affymetrix.com/LinkServlet?probeset=F12) | 10035 | 0.169 | 0.6139 | No |
| 29 | [VWF](https://www.affymetrix.com/LinkServlet?probeset=VWF) | 10299 | 0.131 | 0.6096 | No |
| 30 | [C3AR1](https://www.affymetrix.com/LinkServlet?probeset=C3AR1) | 10359 | 0.119 | 0.6108 | No |
| 31 | [CR1](https://www.affymetrix.com/LinkServlet?probeset=CR1) | 10545 | 0.095 | 0.6079 | No |
| 32 | [THBD](https://www.affymetrix.com/LinkServlet?probeset=THBD) | 11161 | 0.042 | 0.5919 | No |
| 33 | [TFPI](https://www.affymetrix.com/LinkServlet?probeset=TFPI) | 11738 | 0.042 | 0.5770 | No |
| 34 | [F3](https://www.affymetrix.com/LinkServlet?probeset=F3) | 11942 | 0.024 | 0.5720 | No |
| 35 | [BDKRB1](https://www.affymetrix.com/LinkServlet?probeset=BDKRB1) | 12578 | 0.000 | 0.5545 | No |
| 36 | [BDKRB2](https://www.affymetrix.com/LinkServlet?probeset=BDKRB2) | 12579 | 0.000 | 0.5545 | No |
| 37 | [C1QB](https://www.affymetrix.com/LinkServlet?probeset=C1QB) | 12770 | 0.000 | 0.5492 | No |
| 38 | [C1QC](https://www.affymetrix.com/LinkServlet?probeset=C1QC) | 12772 | 0.000 | 0.5492 | No |
| 39 | [C4BPA](https://www.affymetrix.com/LinkServlet?probeset=C4BPA) | 12819 | 0.000 | 0.5479 | No |
| 40 | [C8A](https://www.affymetrix.com/LinkServlet?probeset=C8A) | 12851 | 0.000 | 0.5471 | No |
| 41 | [C8B](https://www.affymetrix.com/LinkServlet?probeset=C8B) | 12852 | 0.000 | 0.5471 | No |
| 42 | [C9](https://www.affymetrix.com/LinkServlet?probeset=C9) | 12861 | 0.000 | 0.5469 | No |
| 43 | [CFB](https://www.affymetrix.com/LinkServlet?probeset=CFB) | 13128 | 0.000 | 0.5395 | No |
| 44 | [CPB2](https://www.affymetrix.com/LinkServlet?probeset=CPB2) | 13347 | 0.000 | 0.5335 | No |
| 45 | [CR2](https://www.affymetrix.com/LinkServlet?probeset=CR2) | 13354 | 0.000 | 0.5333 | No |
| 46 | [F10](https://www.affymetrix.com/LinkServlet?probeset=F10) | 14031 | 0.000 | 0.5147 | No |
| 47 | [F11](https://www.affymetrix.com/LinkServlet?probeset=F11) | 14032 | 0.000 | 0.5147 | No |
| 48 | [F13A1](https://www.affymetrix.com/LinkServlet?probeset=F13A1) | 14034 | 0.000 | 0.5147 | No |
| 49 | [F13B](https://www.affymetrix.com/LinkServlet?probeset=F13B) | 14035 | 0.000 | 0.5147 | No |
| 50 | [F5](https://www.affymetrix.com/LinkServlet?probeset=F5) | 14036 | 0.000 | 0.5147 | No |
| 51 | [F9](https://www.affymetrix.com/LinkServlet?probeset=F9) | 14038 | 0.000 | 0.5147 | No |
| 52 | [FGA](https://www.affymetrix.com/LinkServlet?probeset=FGA) | 14238 | 0.000 | 0.5092 | No |
| 53 | [FGB](https://www.affymetrix.com/LinkServlet?probeset=FGB) | 14239 | 0.000 | 0.5092 | No |
| 54 | [FGG](https://www.affymetrix.com/LinkServlet?probeset=FGG) | 14267 | 0.000 | 0.5084 | No |
| 55 | [KNG1](https://www.affymetrix.com/LinkServlet?probeset=KNG1) | 15576 | 0.000 | 0.4724 | No |
| 56 | [MASP1](https://www.affymetrix.com/LinkServlet?probeset=MASP1) | 17128 | 0.000 | 0.4296 | No |
| 57 | [MBL2](https://www.affymetrix.com/LinkServlet?probeset=MBL2) | 17135 | 0.000 | 0.4294 | No |
| 58 | [PLG](https://www.affymetrix.com/LinkServlet?probeset=PLG) | 20399 | 0.000 | 0.3395 | No |
| 59 | [SERPINA1](https://www.affymetrix.com/LinkServlet?probeset=SERPINA1) | 24472 | 0.000 | 0.2272 | No |
| 60 | [SERPINA5](https://www.affymetrix.com/LinkServlet?probeset=SERPINA5) | 24481 | 0.000 | 0.2270 | No |
| 61 | [PROS1](https://www.affymetrix.com/LinkServlet?probeset=PROS1) | 26569 | -0.008 | 0.1696 | No |
| 62 | [C8G](https://www.affymetrix.com/LinkServlet?probeset=C8G) | 27084 | -0.086 | 0.1574 | No |
| 63 | [F2](https://www.affymetrix.com/LinkServlet?probeset=F2) | 28500 | -0.255 | 0.1243 | No |
| 64 | [PLAT](https://www.affymetrix.com/LinkServlet?probeset=PLAT) | 28510 | -0.257 | 0.1300 | No |
| 65 | [PLAU](https://www.affymetrix.com/LinkServlet?probeset=PLAU) | 29241 | -0.367 | 0.1184 | No |
| 66 | [C5AR1](https://www.affymetrix.com/LinkServlet?probeset=C5AR1) | 30486 | -0.576 | 0.0975 | No |
| 67 | [F7](https://www.affymetrix.com/LinkServlet?probeset=F7) | 30865 | -0.661 | 0.1024 | No |
| 68 | [CFD](https://www.affymetrix.com/LinkServlet?probeset=CFD) | 31491 | -0.796 | 0.1036 | No |
| 69 | [C5](https://www.affymetrix.com/LinkServlet?probeset=C5) | 33311 | -1.295 | 0.0835 | No |

6. KEGG_METABOLISM_OF_XENOBIOTICS_BY_CYTOCHROME_P450

| Dataset | GSEA_Human_AQ1 2 µMvsDMSO |
| --- | --- |
| Phenotype | NoPhenotypeAvailable |
| Upregulated in class | na_pos |
| GeneSet | KEGG_METABOLISM_OF_XENOBIOTICS_BY_CYTOCHROME_P450 |
| Enrichment Score (ES) | 0.5789783 |
| Normalized Enrichment Score (NES) | 1.5651768 |
| Nominal p-value | 0.0 |
| FDR q-value | 0.033017956 |
| FWER p-Value | 0.444 |


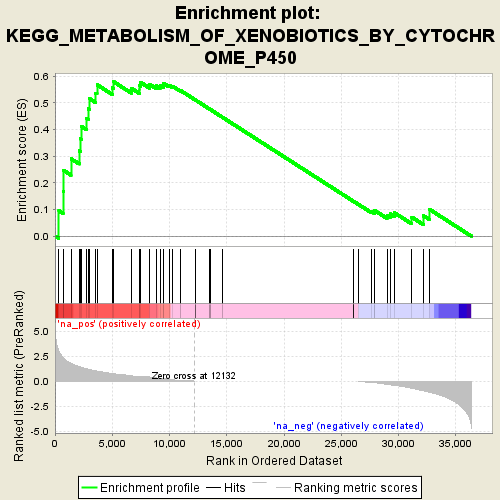


|  | PROBE | RANK IN GENE LIST | RANK METRIC SCORE | RUNNING ES | CORE ENRICHMENT |
| --- | --- | --- | --- | --- | --- |
| 1 | [CYP1A1](https://www.affymetrix.com/LinkServlet?probeset=CYP1A1) | 337 | 3.006 | 0.0950 | Yes |
| 2 | [ALDH3A1](https://www.affymetrix.com/LinkServlet?probeset=ALDH3A1) | 696 | 2.363 | 0.1670 | Yes |
| 3 | [DHDH](https://www.affymetrix.com/LinkServlet?probeset=DHDH) | 736 | 2.331 | 0.2468 | Yes |
| 4 | [GSTM2](https://www.affymetrix.com/LinkServlet?probeset=GSTM2) | 1392 | 1.772 | 0.2902 | Yes |
| 5 | [CYP2E1](https://www.affymetrix.com/LinkServlet?probeset=CYP2E1) | 2109 | 1.438 | 0.3203 | Yes |
| 6 | [ALDH1A3](https://www.affymetrix.com/LinkServlet?probeset=ALDH1A3) | 2226 | 1.388 | 0.3652 | Yes |
| 7 | [GSTA1](https://www.affymetrix.com/LinkServlet?probeset=GSTA1) | 2274 | 1.377 | 0.4117 | Yes |
| 8 | [GSTM1](https://www.affymetrix.com/LinkServlet?probeset=GSTM1) | 2771 | 1.218 | 0.4402 | Yes |
| 9 | [GSTO2](https://www.affymetrix.com/LinkServlet?probeset=GSTO2) | 2889 | 1.190 | 0.4783 | Yes |
| 10 | [EPHX1](https://www.affymetrix.com/LinkServlet?probeset=EPHX1) | 2981 | 1.169 | 0.5163 | Yes |
| 11 | [GSTM3](https://www.affymetrix.com/LinkServlet?probeset=GSTM3) | 3560 | 1.028 | 0.5360 | Yes |
| 12 | [GSTT2](https://www.affymetrix.com/LinkServlet?probeset=GSTT2) | 3662 | 1.001 | 0.5680 | Yes |
| 13 | [ADH6](https://www.affymetrix.com/LinkServlet?probeset=ADH6) | 5033 | 0.739 | 0.5559 | Yes |
| 14 | [AKR1C1](https://www.affymetrix.com/LinkServlet?probeset=AKR1C1) | 5118 | 0.733 | 0.5790 | Yes |
| 15 | [GSTK1](https://www.affymetrix.com/LinkServlet?probeset=GSTK1) | 6658 | 0.521 | 0.5546 | No |
| 16 | [CYP2C18](https://www.affymetrix.com/LinkServlet?probeset=CYP2C18) | 7384 | 0.427 | 0.5494 | No |
| 17 | [CYP3A4](https://www.affymetrix.com/LinkServlet?probeset=CYP3A4) | 7386 | 0.427 | 0.5642 | No |
| 18 | [GSTA3](https://www.affymetrix.com/LinkServlet?probeset=GSTA3) | 7483 | 0.427 | 0.5764 | No |
| 19 | [MGST3](https://www.affymetrix.com/LinkServlet?probeset=MGST3) | 8279 | 0.405 | 0.5685 | No |
| 20 | [CYP2F1](https://www.affymetrix.com/LinkServlet?probeset=CYP2F1) | 8836 | 0.316 | 0.5642 | No |
| 21 | [GSTM5](https://www.affymetrix.com/LinkServlet?probeset=GSTM5) | 9166 | 0.278 | 0.5647 | No |
| 22 | [GSTA4](https://www.affymetrix.com/LinkServlet?probeset=GSTA4) | 9444 | 0.244 | 0.5656 | No |
| 23 | [AKR1C3](https://www.affymetrix.com/LinkServlet?probeset=AKR1C3) | 9492 | 0.237 | 0.5725 | No |
| 24 | [ADH5](https://www.affymetrix.com/LinkServlet?probeset=ADH5) | 9960 | 0.182 | 0.5659 | No |
| 25 | [GSTM4](https://www.affymetrix.com/LinkServlet?probeset=GSTM4) | 10263 | 0.137 | 0.5623 | No |
| 26 | [CYP1A2](https://www.affymetrix.com/LinkServlet?probeset=CYP1A2) | 10994 | 0.042 | 0.5437 | No |
| 27 | [CYP2C8](https://www.affymetrix.com/LinkServlet?probeset=CYP2C8) | 10995 | 0.042 | 0.5451 | No |
| 28 | [ADH1A](https://www.affymetrix.com/LinkServlet?probeset=ADH1A) | 12238 | 0.000 | 0.5109 | No |
| 29 | [ADH1B](https://www.affymetrix.com/LinkServlet?probeset=ADH1B) | 12239 | 0.000 | 0.5109 | No |
| 30 | [ADH1C](https://www.affymetrix.com/LinkServlet?probeset=ADH1C) | 12240 | 0.000 | 0.5109 | No |
| 31 | [ADH4](https://www.affymetrix.com/LinkServlet?probeset=ADH4) | 12241 | 0.000 | 0.5109 | No |
| 32 | [ADH7](https://www.affymetrix.com/LinkServlet?probeset=ADH7) | 12242 | 0.000 | 0.5109 | No |
| 33 | [AKR1C2](https://www.affymetrix.com/LinkServlet?probeset=AKR1C2) | 12300 | 0.000 | 0.5093 | No |
| 34 | [AKR1C4](https://www.affymetrix.com/LinkServlet?probeset=AKR1C4) | 12301 | 0.000 | 0.5093 | No |
| 35 | [CYP1B1](https://www.affymetrix.com/LinkServlet?probeset=CYP1B1) | 13514 | 0.000 | 0.4759 | No |
| 36 | [CYP2B6](https://www.affymetrix.com/LinkServlet?probeset=CYP2B6) | 13523 | 0.000 | 0.4757 | No |
| 37 | [CYP2C19](https://www.affymetrix.com/LinkServlet?probeset=CYP2C19) | 13524 | 0.000 | 0.4757 | No |
| 38 | [CYP2C9](https://www.affymetrix.com/LinkServlet?probeset=CYP2C9) | 13530 | 0.000 | 0.4756 | No |
| 39 | [CYP3A5](https://www.affymetrix.com/LinkServlet?probeset=CYP3A5) | 13533 | 0.000 | 0.4755 | No |
| 40 | [CYP3A7](https://www.affymetrix.com/LinkServlet?probeset=CYP3A7) | 13537 | 0.000 | 0.4754 | No |
| 41 | [GSTA2](https://www.affymetrix.com/LinkServlet?probeset=GSTA2) | 14633 | 0.000 | 0.4452 | No |
| 42 | [GSTA5](https://www.affymetrix.com/LinkServlet?probeset=GSTA5) | 14634 | 0.000 | 0.4452 | No |
| 43 | [UGT1A1](https://www.affymetrix.com/LinkServlet?probeset=UGT1A1) | 26033 | 0.000 | 0.1310 | No |
| 44 | [UGT1A10](https://www.affymetrix.com/LinkServlet?probeset=UGT1A10) | 26034 | 0.000 | 0.1310 | No |
| 45 | [UGT1A3](https://www.affymetrix.com/LinkServlet?probeset=UGT1A3) | 26039 | 0.000 | 0.1309 | No |
| 46 | [UGT1A4](https://www.affymetrix.com/LinkServlet?probeset=UGT1A4) | 26040 | 0.000 | 0.1309 | No |
| 47 | [UGT1A5](https://www.affymetrix.com/LinkServlet?probeset=UGT1A5) | 26041 | 0.000 | 0.1309 | No |
| 48 | [UGT1A6](https://www.affymetrix.com/LinkServlet?probeset=UGT1A6) | 26042 | 0.000 | 0.1309 | No |
| 49 | [UGT1A7](https://www.affymetrix.com/LinkServlet?probeset=UGT1A7) | 26043 | 0.000 | 0.1309 | No |
| 50 | [UGT1A8](https://www.affymetrix.com/LinkServlet?probeset=UGT1A8) | 26044 | 0.000 | 0.1309 | No |
| 51 | [UGT1A9](https://www.affymetrix.com/LinkServlet?probeset=UGT1A9) | 26045 | 0.000 | 0.1309 | No |
| 52 | [UGT2A1](https://www.affymetrix.com/LinkServlet?probeset=UGT2A1) | 26046 | 0.000 | 0.1309 | No |
| 53 | [UGT2A3](https://www.affymetrix.com/LinkServlet?probeset=UGT2A3) | 26048 | 0.000 | 0.1308 | No |
| 54 | [UGT2B10](https://www.affymetrix.com/LinkServlet?probeset=UGT2B10) | 26050 | 0.000 | 0.1308 | No |
| 55 | [UGT2B11](https://www.affymetrix.com/LinkServlet?probeset=UGT2B11) | 26051 | 0.000 | 0.1308 | No |
| 56 | [UGT2B17](https://www.affymetrix.com/LinkServlet?probeset=UGT2B17) | 26052 | 0.000 | 0.1308 | No |
| 57 | [UGT2B28](https://www.affymetrix.com/LinkServlet?probeset=UGT2B28) | 26057 | 0.000 | 0.1307 | No |
| 58 | [UGT2B4](https://www.affymetrix.com/LinkServlet?probeset=UGT2B4) | 26059 | 0.000 | 0.1307 | No |
| 59 | [UGT2B7](https://www.affymetrix.com/LinkServlet?probeset=UGT2B7) | 26060 | 0.000 | 0.1307 | No |
| 60 | [GSTP1](https://www.affymetrix.com/LinkServlet?probeset=GSTP1) | 26533 | -0.003 | 0.1178 | No |
| 61 | [MGST1](https://www.affymetrix.com/LinkServlet?probeset=MGST1) | 27602 | -0.143 | 0.0933 | No |
| 62 | [CYP3A43](https://www.affymetrix.com/LinkServlet?probeset=CYP3A43) | 27894 | -0.160 | 0.0908 | No |
| 63 | [CYP2S1](https://www.affymetrix.com/LinkServlet?probeset=CYP2S1) | 27923 | -0.161 | 0.0956 | No |
| 64 | [UGT2B15](https://www.affymetrix.com/LinkServlet?probeset=UGT2B15) | 28990 | -0.326 | 0.0775 | No |
| 65 | [ALDH3B2](https://www.affymetrix.com/LinkServlet?probeset=ALDH3B2) | 29256 | -0.370 | 0.0831 | No |
| 66 | [ALDH3B1](https://www.affymetrix.com/LinkServlet?probeset=ALDH3B1) | 29626 | -0.427 | 0.0877 | No |
| 67 | [GSTZ1](https://www.affymetrix.com/LinkServlet?probeset=GSTZ1) | 31161 | -0.728 | 0.0706 | No |
| 68 | [GSTO1](https://www.affymetrix.com/LinkServlet?probeset=GSTO1) | 32180 | -0.969 | 0.0762 | No |
| 69 | [MGST2](https://www.affymetrix.com/LinkServlet?probeset=MGST2) | 32709 | -1.109 | 0.1001 | No |

7. KEGG_P53_SIGNALING_PATHWAY

|  |  |
| --- | --- |
| Dataset | GSEA_Human_BAL2vsDMSO |
| Phenotype | NoPhenotypeAvailable |
| Upregulated in class | na_pos |
| GeneSet | KEGG_P53_SIGNALING_PATHWAY |
| Enrichment Score (ES) | 0.51501703 |
| Normalized Enrichment Score (NES) | 1.3792183 |
| Nominal p-value | 0.03504673 |
| FDR q-value | 0.1218395 |
| FWER p-Value | 0.955 |


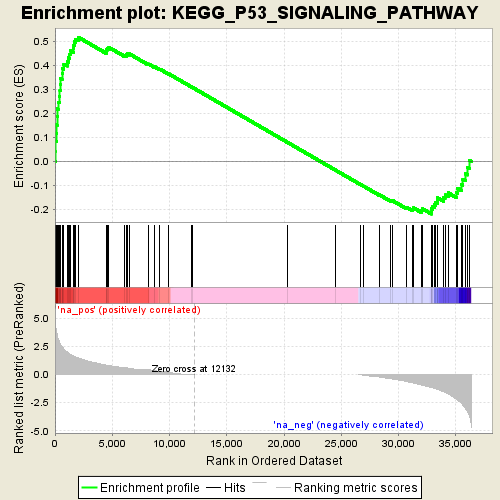


|  | PROBE | RANK IN GENE LIST | RANK METRIC SCORE | RUNNING ES | CORE ENRICHMENT |
| --- | --- | --- | --- | --- | --- |
| 1 | [GADD45A](https://www.affymetrix.com/LinkServlet?probeset=GADD45A) | 46 | 4.423 | 0.0407 | Yes |
| 2 | [TP53I3](https://www.affymetrix.com/LinkServlet?probeset=TP53I3) | 52 | 4.336 | 0.0817 | Yes |
| 3 | [SESN1](https://www.affymetrix.com/LinkServlet?probeset=SESN1) | 115 | 3.893 | 0.1169 | Yes |
| 4 | [CDKN1A](https://www.affymetrix.com/LinkServlet?probeset=CDKN1A) | 147 | 3.680 | 0.1509 | Yes |
| 5 | [TNFRSF10B](https://www.affymetrix.com/LinkServlet?probeset=TNFRSF10B) | 174 | 3.590 | 0.1842 | Yes |
| 6 | [PMAIP1](https://www.affymetrix.com/LinkServlet?probeset=PMAIP1) | 185 | 3.543 | 0.2176 | Yes |
| 7 | [SESN2](https://www.affymetrix.com/LinkServlet?probeset=SESN2) | 264 | 3.212 | 0.2459 | Yes |
| 8 | [BBC3](https://www.affymetrix.com/LinkServlet?probeset=BBC3) | 386 | 2.877 | 0.2698 | Yes |
| 9 | [CCNG2](https://www.affymetrix.com/LinkServlet?probeset=CCNG2) | 426 | 2.783 | 0.2951 | Yes |
| 10 | [GADD45G](https://www.affymetrix.com/LinkServlet?probeset=GADD45G) | 477 | 2.683 | 0.3192 | Yes |
| 11 | [FAS](https://www.affymetrix.com/LinkServlet?probeset=FAS) | 491 | 2.659 | 0.3441 | Yes |
| 12 | [ZMAT3](https://www.affymetrix.com/LinkServlet?probeset=ZMAT3) | 610 | 2.473 | 0.3643 | Yes |
| 13 | [DDB2](https://www.affymetrix.com/LinkServlet?probeset=DDB2) | 652 | 2.412 | 0.3860 | Yes |
| 14 | [MDM2](https://www.affymetrix.com/LinkServlet?probeset=MDM2) | 770 | 2.278 | 0.4044 | Yes |
| 15 | [CCNG1](https://www.affymetrix.com/LinkServlet?probeset=CCNG1) | 1069 | 1.981 | 0.4150 | Yes |
| 16 | [PPM1D](https://www.affymetrix.com/LinkServlet?probeset=PPM1D) | 1162 | 1.916 | 0.4306 | Yes |
| 17 | [SESN3](https://www.affymetrix.com/LinkServlet?probeset=SESN3) | 1281 | 1.832 | 0.4447 | Yes |
| 18 | [RRM2B](https://www.affymetrix.com/LinkServlet?probeset=RRM2B) | 1367 | 1.784 | 0.4593 | Yes |
| 19 | [GADD45B](https://www.affymetrix.com/LinkServlet?probeset=GADD45B) | 1608 | 1.649 | 0.4683 | Yes |
| 20 | [CASP3](https://www.affymetrix.com/LinkServlet?probeset=CASP3) | 1640 | 1.631 | 0.4829 | Yes |
| 21 | [EI24](https://www.affymetrix.com/LinkServlet?probeset=EI24) | 1685 | 1.603 | 0.4969 | Yes |
| 22 | [TP53](https://www.affymetrix.com/LinkServlet?probeset=TP53) | 1797 | 1.556 | 0.5086 | Yes |
| 23 | [RPRM](https://www.affymetrix.com/LinkServlet?probeset=RPRM) | 2065 | 1.452 | 0.5150 | Yes |
| 24 | [SFN](https://www.affymetrix.com/LinkServlet?probeset=SFN) | 4460 | 0.839 | 0.4570 | No |
| 25 | [BAX](https://www.affymetrix.com/LinkServlet?probeset=BAX) | 4470 | 0.837 | 0.4647 | No |
| 26 | [CASP8](https://www.affymetrix.com/LinkServlet?probeset=CASP8) | 4541 | 0.826 | 0.4706 | No |
| 27 | [SIAH1](https://www.affymetrix.com/LinkServlet?probeset=SIAH1) | 4700 | 0.798 | 0.4738 | No |
| 28 | [TP53AIP1](https://www.affymetrix.com/LinkServlet?probeset=TP53AIP1) | 6066 | 0.615 | 0.4420 | No |
| 29 | [SERPINE1](https://www.affymetrix.com/LinkServlet?probeset=SERPINE1) | 6243 | 0.588 | 0.4427 | No |
| 30 | [CCND1](https://www.affymetrix.com/LinkServlet?probeset=CCND1) | 6286 | 0.582 | 0.4471 | No |
| 31 | [RCHY1](https://www.affymetrix.com/LinkServlet?probeset=RCHY1) | 6472 | 0.550 | 0.4472 | No |
| 32 | [CCND2](https://www.affymetrix.com/LinkServlet?probeset=CCND2) | 8161 | 0.418 | 0.4046 | No |
| 33 | [IGFBP3](https://www.affymetrix.com/LinkServlet?probeset=IGFBP3) | 8717 | 0.331 | 0.3924 | No |
| 34 | [SHISA5](https://www.affymetrix.com/LinkServlet?probeset=SHISA5) | 9142 | 0.280 | 0.3834 | No |
| 35 | [THBS1](https://www.affymetrix.com/LinkServlet?probeset=THBS1) | 9913 | 0.190 | 0.3640 | No |
| 36 | [CYCS](https://www.affymetrix.com/LinkServlet?probeset=CYCS) | 11931 | 0.025 | 0.3086 | No |
| 37 | [IGF1](https://www.affymetrix.com/LinkServlet?probeset=IGF1) | 12007 | 0.018 | 0.3067 | No |
| 38 | [PERP](https://www.affymetrix.com/LinkServlet?probeset=PERP) | 20294 | 0.000 | 0.0783 | No |
| 39 | [SERPINB5](https://www.affymetrix.com/LinkServlet?probeset=SERPINB5) | 24492 | 0.000 | -0.0374 | No |
| 40 | [CDKN2A](https://www.affymetrix.com/LinkServlet?probeset=CDKN2A) | 26635 | -0.019 | -0.0963 | No |
| 41 | [PTEN](https://www.affymetrix.com/LinkServlet?probeset=PTEN) | 26947 | -0.062 | -0.1043 | No |
| 42 | [MDM4](https://www.affymetrix.com/LinkServlet?probeset=MDM4) | 28318 | -0.223 | -0.1399 | No |
| 43 | [TSC2](https://www.affymetrix.com/LinkServlet?probeset=TSC2) | 29305 | -0.376 | -0.1635 | No |
| 44 | [CDK4](https://www.affymetrix.com/LinkServlet?probeset=CDK4) | 29439 | -0.398 | -0.1634 | No |
| 45 | [CDK1](https://www.affymetrix.com/LinkServlet?probeset=CDK1) | 30724 | -0.629 | -0.1929 | No |
| 46 | [BID](https://www.affymetrix.com/LinkServlet?probeset=BID) | 31221 | -0.743 | -0.1995 | No |
| 47 | [CDK2](https://www.affymetrix.com/LinkServlet?probeset=CDK2) | 31270 | -0.754 | -0.1937 | No |
| 48 | [ATM](https://www.affymetrix.com/LinkServlet?probeset=ATM) | 32022 | -0.933 | -0.2055 | No |
| 49 | [APAF1](https://www.affymetrix.com/LinkServlet?probeset=APAF1) | 32044 | -0.940 | -0.1972 | No |
| 50 | [CD82](https://www.affymetrix.com/LinkServlet?probeset=CD82) | 32873 | -1.157 | -0.2090 | No |
| 51 | [CCNB2](https://www.affymetrix.com/LinkServlet?probeset=CCNB2) | 32880 | -1.158 | -0.1982 | No |
| 52 | [ATR](https://www.affymetrix.com/LinkServlet?probeset=ATR) | 32984 | -1.189 | -0.1898 | No |
| 53 | [CASP9](https://www.affymetrix.com/LinkServlet?probeset=CASP9) | 33131 | -1.240 | -0.1820 | No |
| 54 | [GTSE1](https://www.affymetrix.com/LinkServlet?probeset=GTSE1) | 33236 | -1.268 | -0.1729 | No |
| 55 | [CCNB1](https://www.affymetrix.com/LinkServlet?probeset=CCNB1) | 33374 | -1.316 | -0.1642 | No |
| 56 | [CHEK1](https://www.affymetrix.com/LinkServlet?probeset=CHEK1) | 33418 | -1.331 | -0.1528 | No |
| 57 | [CCNE1](https://www.affymetrix.com/LinkServlet?probeset=CCNE1) | 33941 | -1.536 | -0.1526 | No |
| 58 | [CCNE2](https://www.affymetrix.com/LinkServlet?probeset=CCNE2) | 34055 | -1.587 | -0.1407 | No |
| 59 | [CHEK2](https://www.affymetrix.com/LinkServlet?probeset=CHEK2) | 34335 | -1.719 | -0.1320 | No |
| 60 | [STEAP3](https://www.affymetrix.com/LinkServlet?probeset=STEAP3) | 35061 | -2.187 | -0.1313 | No |
| 61 | [CCND3](https://www.affymetrix.com/LinkServlet?probeset=CCND3) | 35153 | -2.264 | -0.1123 | No |
| 62 | [RRM2](https://www.affymetrix.com/LinkServlet?probeset=RRM2) | 35490 | -2.580 | -0.0971 | No |
| 63 | [TP73](https://www.affymetrix.com/LinkServlet?probeset=TP73) | 35617 | -2.715 | -0.0748 | No |
| 64 | [RFWD2](https://www.affymetrix.com/LinkServlet?probeset=RFWD2) | 35863 | -3.028 | -0.0529 | No |
| 65 | [CCNB3](https://www.affymetrix.com/LinkServlet?probeset=CCNB3) | 36034 | -3.326 | -0.0260 | No |
| 66 | [CDK6](https://www.affymetrix.com/LinkServlet?probeset=CDK6) | 36162 | -3.629 | 0.0049 | No |
